# Supplementary material for: Safety and efficacy of an iBTA-induced autologous Biotube vascular graft and its preparation device BTM1 in below-the-knee bypass surgery for chronic limb threatening ischemia: A protocol for an open-label, single-arm, multicenter clinical trial
Source: PLoS One. 2025 Nov 6;20(11):e0335900. doi: 10.1371/journal.pone.0335900 (PMC12591420; doi:10.1371/journal.pone.0335900)
Supplement: S2 File — (PDF) [file pone.0335900.s002.pdf]

重症下肢虚血患者に対する **BTM1** の皮下埋植及び

BTM1 で得られたバイオチューブを用いた

下肢への動脈バイパス術の安全性及び有効性を評価する

多施設共同単一群探索的試験

（医師主導治験）

治験実施計画書

治験調整医師

大分大学医学部附属病院　心臓血管外科

宮本　伸二

治験実施計画書番号：B-80

版番号：第 2.0 版

作成年月日：2022 年 9 月 5 日

本文書に含まれる情報は大分大学が所有しております。大分大学は、本文書に含まれる情報を本治験にご参加いただける治験責任医師（治験スタッフも含む）、治験機器管理者、治験審査委員会及び実施医療機関の長に提供しております。このため、参加される患者様から同意をいただく場合及び適用される法律により開示を要求される場合を除き、大分大学の事前の文書による同意なしに、本治験に関係のない第三者にこの情報を開示することはできません。開示する場合には、事前に治験調整医師及び治験機器提供者の承認を得るようお願いいたします。

目次

1. 開発の経緯 1
1.1 治験実施計画の背景 **1**
1.2 本治験の位置づけ・妥当性 **1**
1.3 非臨床試験の要約 **1**
1.4 被験機器 **2**
1.5 被験者に対する既知及び可能性のあるリスクとベネフィットの要約 **7**
1.5.1 本被験機器使用により予想されるリスク 7
1.5.2 本被験機器使用により予想されるベネフィット 7
2. 治験の目的 8
3. 治験のデザイン 8
3.1 治験の種類及びデザイン **8**
3.1.1 治験の種類 8
3.1.2 治験のデザイン 8
3.2 評価項目 **8**
3.3 被験者の治験参加期間 **9**
3.4 症例登録の制限 **9**
3.5 治験実施予定期間 **11**
4. 被験者の選択・除外、中止基準 11
4.1 対象疾患 **11**
4.2 選択基準 **11**
4.3 除外基準 **11**
4.4 中止基準 **12**
5. 併用薬、併用療法及び検査に関する規定 13
5.1 併用薬 **13**
5.2 併用療法 **13**
5.3 検査の制限 **13**
6. 治験の手順 13
6.1 同意取得 **13**
6.2 適格性確認 **14**
6.3 被験者登録 **14**
6.4 被験機器の使用 **14**
6.4.1 治験の手順 14
6.4.2 被験機器 **BTM1** の使用手順 14
6.4.3 バイオチューブ移植（バイパス術）に関する事項 17
6.4.4 被験機器の埋植期間及び後観察期間 18
6.5 観察・検査スケジュール **20**
6.5.1 観察・検査・調査内容 22

6.5.2 中止後の検査・観察 26
7. 安全性評価 27
7.1 臨床検査値 27
7.2 有害事象 27
7.2.1 有害事象の定義 27
7.2.2 有害事象の記載 28
7.2.3 重篤な有害事象 28
7.2.4 有害事象の重篤性の判定 28
7.2.5 有害事象の治療のためにとられた処置 28
7.2.6 被験機器に対してとられた処置 28
7.2.7 有害事象の転帰 29
7.3 不具合 29
7.3.1 不具合 29
7.3.2 不具合情報の記録 29
7.4 有害事象及び不具合発生時の対応 30
7.5 重篤な有害事象及び重篤な有害事象につながる恐れがある不具合が発生した場合の報告 30
7.6 新たな情報の提供 31
8. 有効性評価 31
8.1 主要評価項目 31
8.2 副次評価項目 31
9. 統計解析 33
9.1 解析対象集団 33
9.2 有効性の解析 33
9.3 安全性の解析 33
9.4 中間解析 34
9.5 症例数の設定 34
10. 治験の品質管理及び品質保証 34
10.1 本治験における品質マネジメント 34
10.2 原資料等の直接閲覧 34
10.3 モニタリング 35
10.4 監査 35
10.5 モニタリング及び監査への協力 35
11. 倫理及び GCP 遵守 35
11.1 治験の倫理的実施 35
11.2 被験者への説明と同意の取得 35
11.3 同意取得の方法 36
11.4 説明文書の改訂 36
11.5 治験審査委員会 37
11.6 被験者の人権保護に関する事項 37

12. 症例報告書 37
12.1 記載方法 **37**
12.2 原資料 **37**
12.3 症例報告書中の記載内容を原資料とすべき項目の特定 **38**
13. 治験の費用負担及び補償 38
13.1 利益相反について **38**
13.2 健康被害補償及び保険 **38**
13.3 予定される治験費用及び被験者への支払い **39**
14. 治験実施計画書の逸脱または変更及び改訂 39
14.1 治験実施計画書の逸脱または変更 **39**
14.2 治験実施計画書の改訂 **39**
15. 治験の中止・中断・終了 39
15.1 治験の中止・中断 **39**
15.2 治験の終了 **40**
16. 効果安全性評価委員会について 40
17. 記録等の保存 40
18. 公表に関する取決め 40
19. 治験実施体制 40
20. 参考文献 41

略語・略号及び用語の定義・内容一覧表

ABI	Ankle Brachial Index	足関節上腕血圧比
AE	Adverse Event	有害事象
ALT（GPT）	Alanine Aminotransferase<br>（Glutamic Pyruvic Transaminase）	アラニンアミノトランスフェラーゼ<br>（グルタミン酸ピルビン酸トランスアミナーゼ）
APTT	Activated Partial Thromboplastin Time	活性化部分トロンボプラスチン時間
AST（GOT）	Aspartate Aminotransferase<br>（Glutamic Oxaloacetic Transaminase）	アスパラギン酸アミノトランスフェラーゼ<br>（グルタミン酸オキサロ酢酸トランスアミナーゼ）
BUN	Blood Urea Nitrogen	血清尿素窒素
CLTI	Chronic Limb Threatening Ischemia	包括的高度慢性下肢虚血
CONUT	Controlling Nutritional Status	血液検査値から得られる栄養指標
CRF	Case Report Form	症例報告書
CRP	C-Reactive Protein	C 反応性タンパク質
CT	Computed Tomography Scan	コンピュータ断層診断装置
GLASS	Global Limb Anatomic Staging System	GVG 委員会が提唱する四肢閉塞部位の解剖学的分類システム
GVG	Global Vascular Guideline	世界的な血管ガイドライン
IRB	Institutional Review Board	治験審査委員会
LDH	Lactate Dehydrogenase	乳酸脱水素酵素
LDL	Low-density Lipoprotein	低比重リポタンパク質
PT	Prothrombin Time	プロトロンビン時間
PT-INR	International Normalized Ratio of Prothrombin Time	プロトロンビン時間国際標準比
QOL	Quality of Life	生活の質
SPP	Skin Perfusion Pressure	皮膚灌流圧
WIfI	Wound, Ischemia, and foot Infection	足病変の状態、虚血の重症度、足部感染の程度

治験実施計画の概要

治験名	重症下肢虚血患者に対するBTM1の皮下埋植及びBTM1で得られたバイオチューブを用いた下肢への動脈バイパス術の安全性及び有効性を評価する多施設共同単一群探索的試験（医師主導治験）
目的	本治験は、バイパス術に使用可能な至適な自家静脈が存在しない重症下肢虚血患者を対象とし、被験機器「BTM1」を用いて患者皮下で作製したバイオチューブを用いて膝下の下腿動脈あるいは足部動脈へのバイパス術を施行することで、その安全性と有効性を評価することを目的とする。
被験機器	BTM1
治験デザイン	多施設共同、非盲検、非対照単一群、探索的治験
対象患者	膝下の下腿動脈あるいは足部動脈へ末梢吻合するバイパス術を必要とするが、至適な自家静脈が存在しない重症下肢虚血患者
選択基準<br>（登録時）	1. 治験参加に関して、本人から文書による同意が得られた患者<br>2. 同意取得時の年齢が18歳以上である患者<br>3. 包括的高度慢性下肢虚血（Chronic Limb Threatening Ischemia; CLTI）の重症度WIfI分類において虚血（Ischemia）grade 2または3（ABI <0.6, SPP <40 mmHg）を満たす重症下肢虚血患者<br>4. 下肢動脈バイパス術が推奨される患者のうち、以下のいずれかに該当する患者<br>a. Global Vascular Guideline（GVG）のGLASS（Global Anatomic Staging System）分類においてstage IIIの虚血肢で、WIfI分類clinical stage 2、3または4に該当する<br>b. GLASS stage IIの虚血肢で、WIfI分類clinical stage 3または4に該当する<br>c. GLASS stage IまたはIIの虚血肢で、血管内治療を行っても十分な血流を得られない（創傷の治癒につながらず臨床症状の改善が得られない臨床的不成功を含む）<br>5. バイパス術に必要な長さ及び径（3 mm以上）を満たし、瘤化等の異常所見がない至適な上肢または下肢静脈（透析患者の場合、下肢静脈）が存在しない患者<br>6. 膝下の下腿動脈あるいは足部動脈へのバイパスの末梢吻合を必要とする患者<br>7. 同意取得時点において12ヶ月以上の生存及びバイパス術後12週間の経過観察が可能であると判断される患者
除外基準<br>（登録時）	1. 直ちに血行再建術が必要な状態である等の理由により、バイオチューブ形成に必要な被験機器の埋植期間の確保が困難な全身状態である患者<br>2. 重度の低栄養（CONUTスコア8～12）や重症の合併症等により、手術認容が困難と判断される全身状態である患者

	3. 皮膚の状態が不良である、過去に皮下埋植物の露出歴がある等の理由により、被験機器の埋植部位を 2 ヶ所以上確保できない可能性がある患者<br>4. 登録前 30 日以内に侵襲を伴う外科手術を受けている患者（足の創傷に対する処置を行った場合は除く）<br>5. バイパス術に必要な末梢側の標的動脈が存在しない、あるいは末梢側の吻合予定部位に血管内治療施行歴がある患者<br>6. バイパス術の中枢側の吻合予定部位より近位に動脈の閉塞が認められる患者<br>7. バイパス術の末梢側の吻合予定部位より遠位の血行が確認できない患者（側副血行が確認できる場合を除く）<br>8. 中足部より近位での下肢切断が施行されている患者<br>9. 悪性腫瘍の既往や合併のある患者（治療後 5 年間以上再発を認めない、もしくは新規の発症がないものを除く）<br>10. 自己免疫疾患の合併や移植後等のため免疫抑制剤を使用中の患者<br>11. ステンレス鋼またはポリオレフィン樹脂にアレルギーの既往がある患者<br>12. 妊娠している、または妊娠の可能性がある患者<br>13. 他の治験または介入のある臨床研究に参加中もしくは参加予定である患者<br>14. その他、病状等や安全上の理由により、治験責任医師または治験分担医師が本治験の対象として不適切であると判断した患者
中止基準	以下に該当する被験者については、治験を中止する。<br>1. 治験責任医師または治験分担医師が治験継続困難と判断する有害事象の発現や原疾患の増悪がみられた場合<br>2. バイパス術に使用できるバイオチューブが、断片状のものも含めて全く形成されず、それ以降の治験継続が不可能となった場合<br>3. 被験者が治験参加の同意を撤回した場合<br>4. 登録後に被験者が適格性を満たしていないことが判明した場合<br>5. 被験機器埋植後かつバイオチューブ移植前に被験者の妊娠が判明した場合<br>6. 治験実施計画書からの重大な逸脱が判明した場合<br>7. BTM1 植込み後 24 週経過後もバイパス術が不要だった場合、BTM1 は全て取り出し、治験は中止とするが、バイオチューブの形成能は評価する。<br>8. その他、治験責任医師または治験分担医師が本治験を中止すべきと判断した場合
検査・観察項目	別表（観察・検査スケジュール）のとおり
有効性評価項目	【主要評価項目】<br>被験機器によるバイオチューブの形成能<br>【副次評価項目】<br>1) 被験機器の埋植・摘出に関する手技的成功<br>2) 被験機器によって形成されたバイオチューブを用いた末梢側吻合を含むバイパス術の手技的成功

	3) 移植後のバイオチューブの 12 週時点の開存<br>4) 症状の改善（虚血性疼痛の改善、創傷の改善）<br>5) 大切断の回避<br>6) 被験機器埋植中及びバイオチューブ移植後の追加治療の実施率<br>7) 被験機器埋植中及びバイオチューブ移植後の QOL<br>8) バイパス術後のバイパスグラフト径
安全性評価項目	1) 被験機器の皮下埋植期間中の被験機器との関連を否定できない炎症、腫瘍形成、死亡の発生<br>2) 移植したバイオチューブの生体適合性（炎症、腫瘍形成等）<br>3) 移植したバイオチューブの破裂<br>4) 移植したバイオチューブとの関連を否定できない死亡の発生<br>5) バイオチューブの移植後 12 週までの死亡の発生<br>6) その他、有害事象及び不具合の発生
目標症例数	12 例
治験実施期間	2022 年 8 月～2026 年 5月（登録期間：2022 年 8 月～2025 年 11 月）

【表】観察・検査スケジュール

実施時期 / 観察項目	前観察期			被験機器埋植期						後観察期							中止時	
	Visit（V） 1			V2		V3	V4	V5 ※4	V6 ※5	V7	V8	V9	V10	V11	V12	V13		
	同意取得	適格性確認	被験者登録	被験機器埋植		ブレイクドレーン抜去	ドレーン抜去後1週	被験機器埋植後6週	治験機器摘出	バイオチューブ移植	移植後1日	移植後3日	移植後1週	移植後4週	移植後8週	移植後12週	被験機器埋植期間中	後観察期間中
許容範囲	V2 −8日					V2＋2〜7日	V3＋5〜8日	V2＋5〜7週	V2＋4〜24週	V6＋0日〜4週			±2日	±7日	±7日	±7日	+7日	+7日
同意取得	●																	
被験者背景（身長・体重以外）		●																
被験者登録			●															
身長・体重		●※2																
自他覚所見		●		●※3				●	●	●※6	●	●	●	●	●	●	●	●
バイタルサイン		●※2		●				●	●	●※6	●	●	●	●	●	●	●	●
血液検査		●※2		●※3				●	●	●※6	●	●	●	●	●	●	●	●
下肢動脈エコー検査		●※2						●※4					●	●	●	●	●	●
上肢・下肢静脈エコー検査		●※2																
下肢 CT 血管造影検査 ※1		▲※2											▲			△		
下肢動脈造影検査 ※1		▲※2											▲			△		
下肢 X 線検査		●※2											●	●	●	●	●	●
ABI 検査		●※2											●	●	●	●	●	●
SPP 検査		●※2											●	●	●	●	●	●
虚血状態評価		●											●	●	●	●	●	●
下肢 MRI 検査		○※2																
第三者による適格性確認		●																
被験機器埋植					●													
被験機器埋植部位エコー検査						●	●											
ブレイクドレーン抜去						●												
アンケート調査								●※4								●	●	●
被験機器摘出									●								●	
バイオチューブ品質評価 ※7									●	●※7								
バイオチューブ試料保存									●	○※8							●	
バイオチューブ移植										●								
併用薬・併用療法		←	―	―	―	―	―	―	―	―	―	―	―	―	―	→	●	●
有害事象					←	―	―	―	―	―	―	―	―	―	―	→	●	●
不具合 ※9					←	―	―	―	―	―	―	―	―	―	―	→	●	

●：必須　○：任意で実施または可能な場合に実施　▲：両方またはいずれかを実施　△：両方またはいずれかを可能な場合に実施

※1：両方またはいずれか一方の検査を実施する。
※2：同一の検査（調査）項目の結果が複数存在する場合は、直近のものを採用する。許容された期間内に治験実施医療機関で実施した検査（調査）の結果がある場合は、同意取得前に得られた結果であっても使用可能とする。なお、第三者による適格性確認の前に実施すること。
※3：適格性確認時と状態が変わっていない等の理由で治験責任医師または治験分担医師が検査（調査）不要と判断する場合は必須としない。
※4：被験機器埋植期間を短縮せざるを得ず、Visit 5 をスキップする（規定された Visit 5 の実施時期より前に被験機器摘出（Visit 6）を実施する）ことになった場合は、下肢動脈エコー検査及びアンケート調査を被験機器摘出より前に実施する。
※5：埋植後に著しい虚血の進行がみられた等、被験者の安全性確保のためにやむを得ない理由がない限り、原則として、Visit 2 から 8 週間以上経過後に実施する。
※6：Visit 6（被験機器摘出）と同日～7 日以内に Visit 7 を実施する場合は、必須としない。Visit 6 と Visit 7 の間が 8 日以上空く場合、Visit 7 のバイオチューブ移植前に実施する。ただし、Visit 6 と Visit 7 を同日中に実施しない理由が被験者の病状悪化であった場合は、Visit 6 と Visit 7 の間の日数によらず、可能な限り実施すること。
※7：バイオチューブの外観、耐圧性、強度を評価する。Visit 6（被験機器摘出）と同日に Visit 7 を実施する場合は、被験機器摘出からバイオチューブ移植前までの間に一度実施することで差し支えないが、Visit 6 と同日に Visit 7 を実施せずバイオチューブを一時保存する場合は、移植術の実施前に、一時保存後のバイオチューブについて再度品質評価を実施すること。
※8：バイオチューブの病理組織評価を行うための試料を保存する。Visit 6（被験機器摘出）と同日に Visit 7 を実施せずバイオチューブを一時保存する場合で、かつ試料の量が十分な場合に、一時保存後のバイオチューブも病理組織評価用に保存する。
※9：バイオチューブは被験機器ではないが、本治験の特性に鑑み、被験機器摘出後もバイオチューブの不具合について調査を行う。

1. 開発の経緯

1.1 治験実施計画の背景

重症下肢虚血症は、虚血性安静時疼痛、足潰瘍・壊疽等を呈し、本邦でも年間約 1 万人もの患者において足切断が行われており、その切断後の予後も 1 年以内の死亡率が 25%、対側の足の切断率が 25%と極めて悪く、生命に直結する病態である（参考文献 1, 2)。血行再建ができなければ下肢の切断に至る。一般的に下肢の血行再建は、カテーテル治療である血管内治療と、自家静脈あるいは人工血管を用いてのバイパス術がある（参考文献 3)。膝下膝窩動脈から下腿動脈に対する血管内治療は再狭窄、閉塞が多く、細い人工血管によるバイパスも成績不良で（参考文献 4 – 6)、自家静脈を使用してのバイパスが最も成績が良い（参考文献 7, 8)。しかしながら、静脈瘤である、静脈が細い、すでに冠動脈や末梢動脈のバイパスに使用済みである等で自家静脈が使えない場合は救肢困難となる。

下肢膝下のバイパスに使用できる細く長い人工血管が待ち望まれているが、ePTFE（延伸ポリテトラフルオロエチレン）や PET（ポリエチレンテレフタレート）等の人工素材を用いた人工血管は大口径（10 mm 以上）では開存性、耐久性等においてほぼ満足のいく結果が得られているが、中口径（6～8 mm）では不十分であり、下肢末梢バイパスや冠動脈バイパスに用いるような小口径（5 mm 以下）では満足できる製品が存在しない。生体材料由来や従来の組織工学を用いた人工血管も未だ実用的なものは完成していない。中山が開発した生体内組織形成術（in-Body Tissue Architecture; iBTA）を用いると、鋳型を 1～2 ヶ月間皮下に埋め込むことで、下肢の膝下でのバイパスに使用可能な細く、長い自己管状組織（バイオチューブ）ができあがる（参考文献 9 - 13)。バイオチューブは、従来血行再建不能であった重症下肢虚血患者に対する、外科的な血行再建を可能とし、膝下血行不全病態の改善及び自己血管再生による長期開存が期待される。本被験機器は平成 31 年度の厚生労働省の先駆け審査指定制度によって医療機器として指定を受け、クラス III の新医療機器に該当する。令和元年度の国立研究開発法人日本医療研究開発機構（AMED）医工連携事業の支援によって鋳型の製品仕様の決定と非臨床試験法の準備を終えた。

1.2 本治験の位置づけ・妥当性

本治験は、被験機器 BTM1 の生体内組織形成術に基づくバイオチューブ形成能の評価を主たる目的とした探索的医師主導治験である。

本研究では、令和元年 11 月に実施した独立行政法人医薬品医療機器総合機構（PMDA）との医療機器開発前相談にて助言された概念的要求事項に基づき、令和 2 年度 AMED 橋渡し研究戦略的推進プログラムのもと、非臨床試験（有効性試験、性能試験、安全性試験）を実施した。また、ヤギを用いた埋植試験においては、BTM1 によるバイオチューブの形成能を確認した。さらにヤギへのバイオチューブの移植試験を行い（参考文献 14)、約 3 ヶ月でバイオチューブが血管組織に置き換わっていること及び 12 ヶ月の開存を確認している。

検証的治験による本治療の有効性及び安全性評価に先立ち、本治験ではまずヒトにおける BTM1 のバイオチューブ形成能を検証する。

1.3 非臨床試験の要約

本被験機器は実際には治療も診断も行わない。本被験機器を用いて出来上がるバイオチューブが移植物として治療に使用される。そのため、本被験機器とバイオチューブに関してそれぞれ以下の要求事項が

求められた。本被験機器に関しては、1) 皮下埋込が可能であり、2) 埋植後もバイオチューブ形成に必要な形状（中芯と外殻の隙間を含む）及び 3) 強度を維持していること、4) 一定期間の埋植後、外科的バイパス術に使用可能なバイオチューブが形成され、5) 本品及び 6) バイオチューブを損傷なく摘出可能であること、7) 医療機器としての安全性を有していることである。また、バイオチューブに関しては、8) 臨床上必要となる形状及び品質（厚み、均一性を含む）を有し、9) 機械的強度・柔軟性を有すること、10) バイオチューブが移植できること、11) 移植後に、バイオチューブを足場として、自己細胞が浸潤し、自己血管に類似した組織が形成されること、12) 臨床上必要とされる期間、バイオチューブが分解・吸収されないこと、13) 使用部位において、吻合不全が起こらず、長期的な開存が得られること、さらに、14) バイオチューブが連結可能であること、15) バイオチューブが保存できることである。本被験機器とバイオチューブへの各要求事項に対して、安全性と有効性、安定性の検証に必要と考えられる試験項目を選択して実施した。また、関連する付属品であるサイザー、直線化棒、浸漬皿に関しても必要と考えられる試験を行った。全ての試験に適合し、本被験機器がヒトに使用するにあたり、妥当な性能、安全性及び有効性を有することを確認した。

1.4　被験機器

1.4.1 被験機器の名称及び構成

本被験機器の名称は「BTM1」で、「中芯」、「外殻」（左右対称形 2 種）、「内枠」及び「外枠」よりなり、生成されるバイオチューブの長さによって、40 cm 用と 55 cm 用の 2 種類が存在する。

また、「サイザー」、「直線化棒」及び「浸漬皿」を付属品として含む。

【被験機器：BTM1 の写真（左：55 cm 用、右：40 cm 用）】

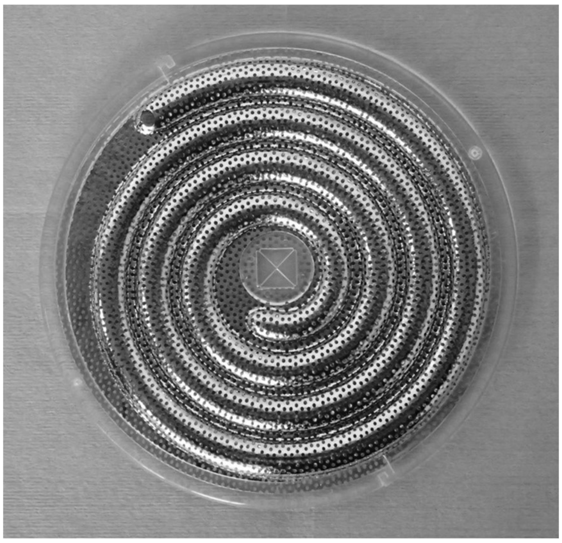

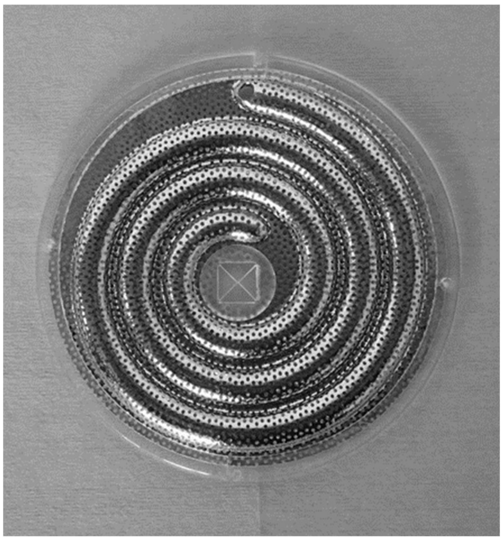

【被験機器：BTM1 の構造及びサイズ（上：55 cm 用、下：40 cm 用）】

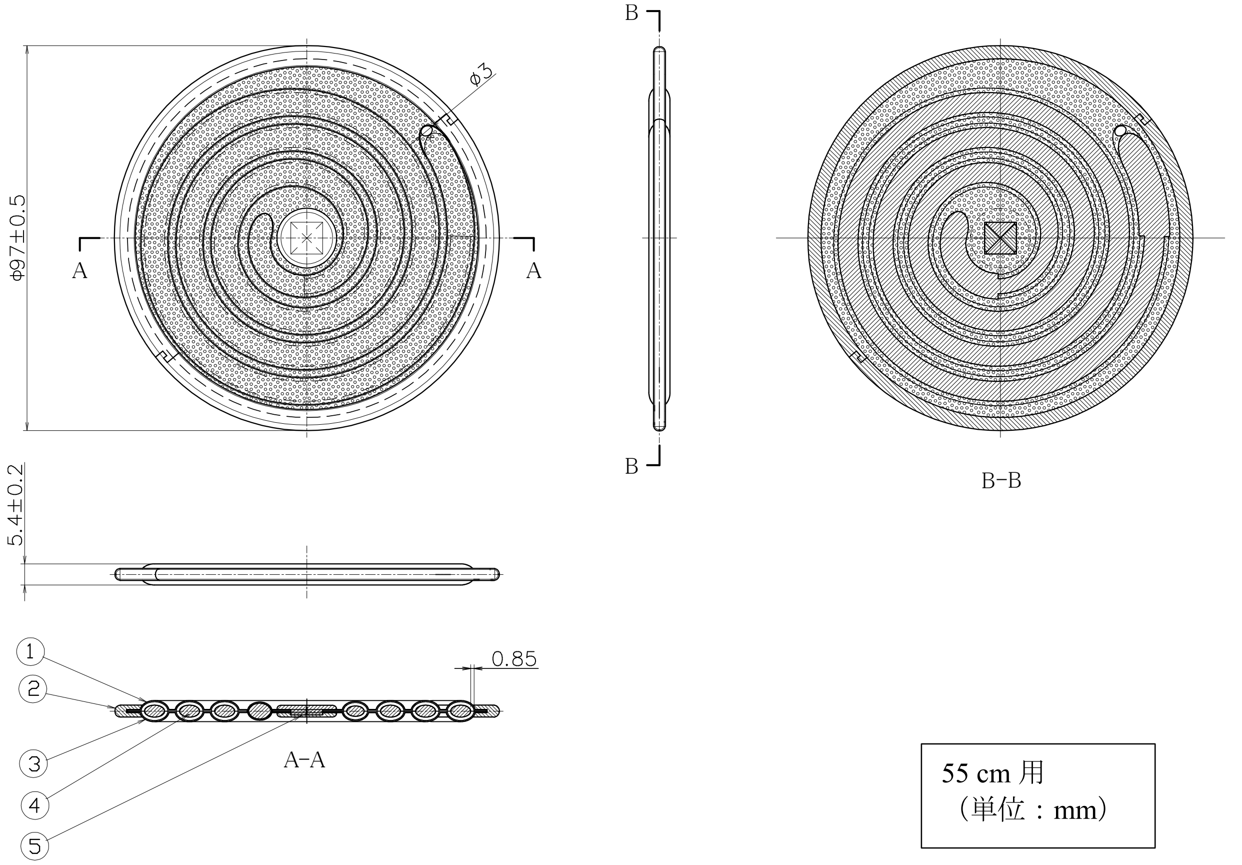

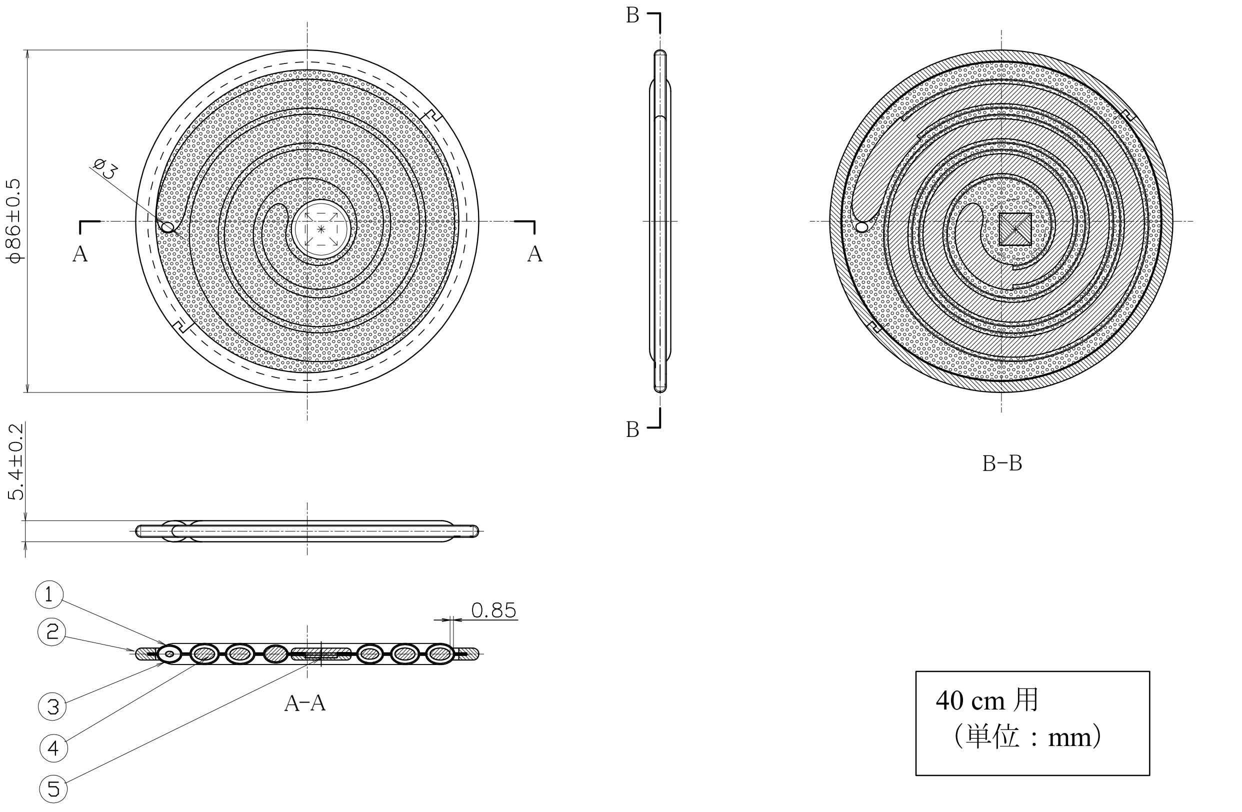

表 1.4.1.3　各部の名称

番号	名称
①	外殻 A
②	外枠
③	中芯
④	外殻 B
⑤	内枠

【付属品：サイザーの写真（左：55 cm 用、右：40 cm 用）】

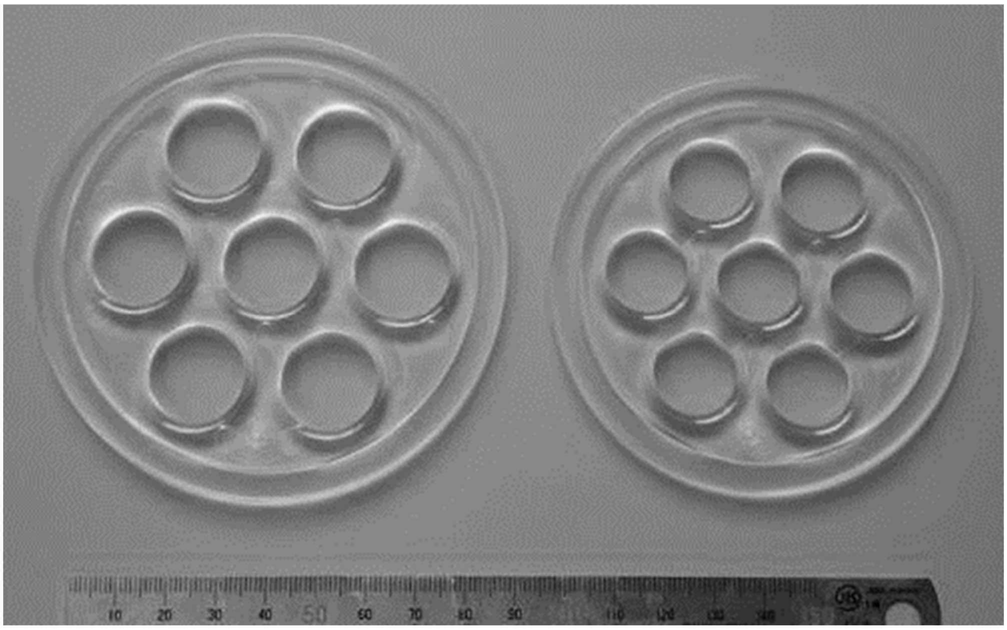

【付属品：サイザーの構造及びサイズ（左：55 cm 用、右：40 cm 用）】

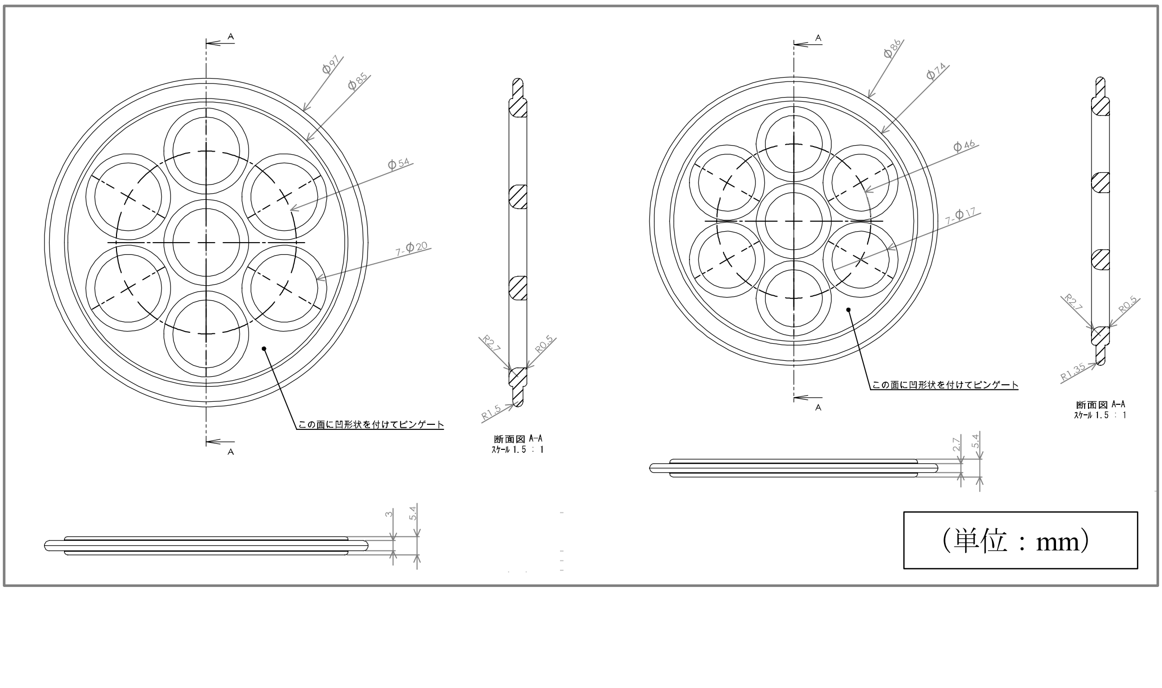

【付属品：直線化棒の写真】

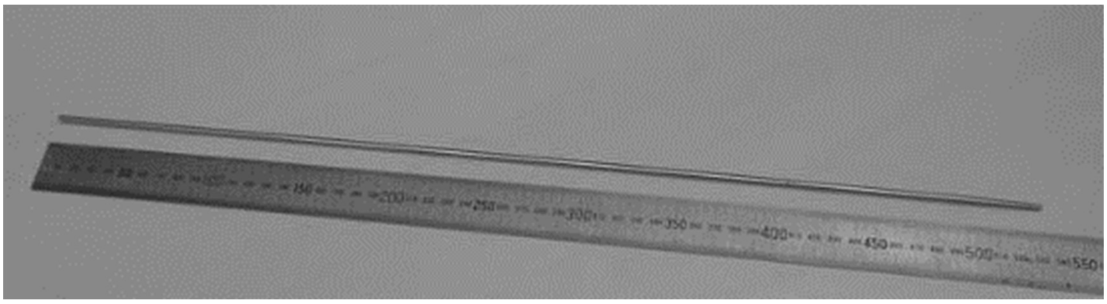

【付属品：直線化棒の構造及びサイズ】

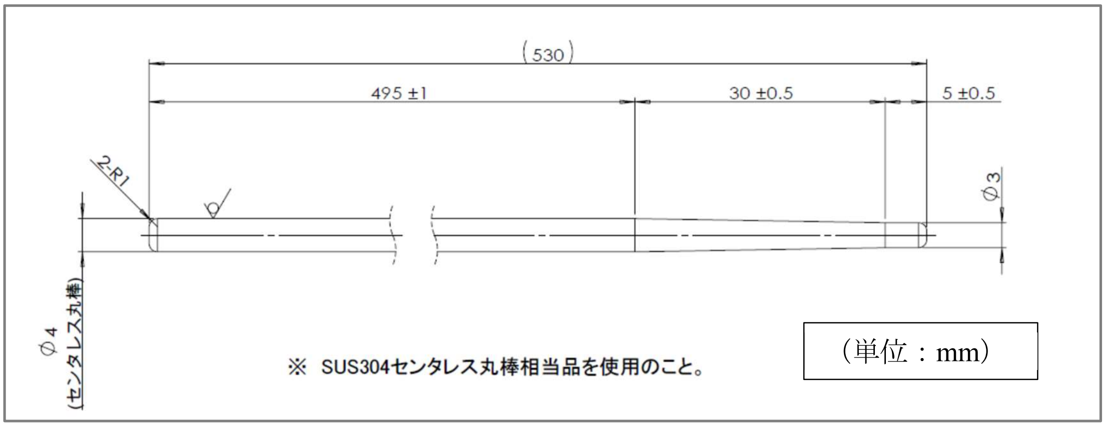

【付属品：浸漬皿の写真】

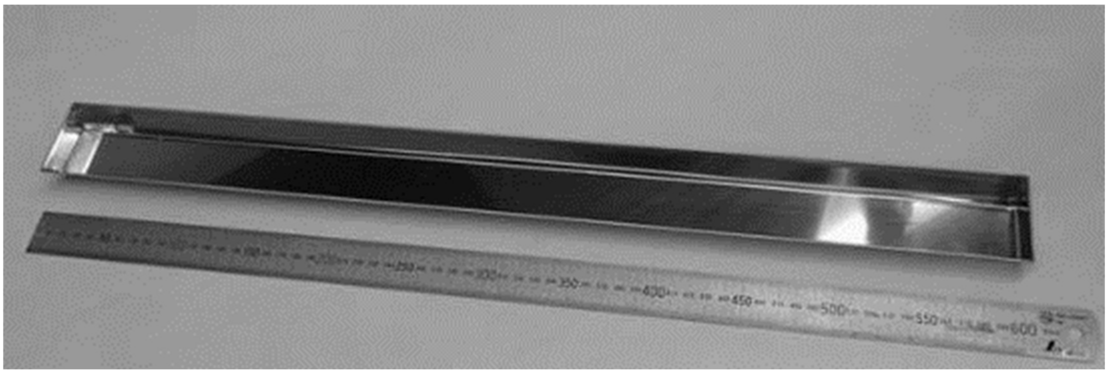

【付属品：浸漬皿の構造及びサイズ】

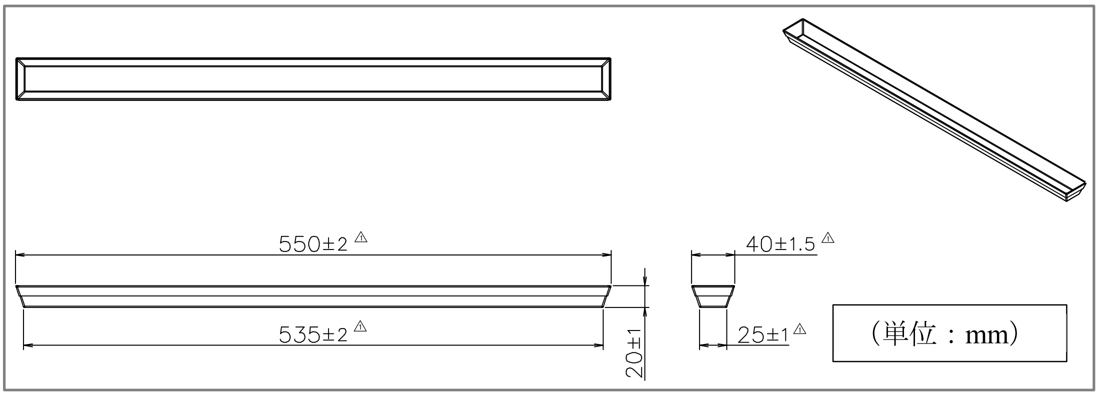

1.4.2 表示

(1) 包装形態

1 箱あたり、滅菌袋内に密封された BTM1 を 1 個収める。

(2) 外装表示

被験機器外装には、治験用であること、治験実施計画書番号、治験識別記号、被験機器のサイズ、製造番号、保管方法、使用期限、治験調整医師の氏名、所属、職名及び住所を以下のとおりに表示する。

治験用　治験実施計画書番号：B-80

BTM1

（●● cm）

治験識別記号：BTM1
製造番号　：XXXXXXX
保管方法　：室温保存
使用期限　：20XX年XX月XX日

治験調整医師
氏名　：宮本　伸二
所属・職名：大分大学医学部附属病院　心臓血管外科・医師
住所　：大分県由布市挟間町医大ヶ丘1丁目1番地

1.4.3 保管及び管理手順

本被験機器は各実施医療機関にて高温多湿を避けて室温で保管する。被験機器の適切な保管及び管理のため、治験機器管理者を置くものとする。治験機器管理者は「治験機器の管理に関する手順書」に準じ、被験機器を適切に保管するとともに、被験機器の使用状況及び治験の進行状況を把握するために被験機器管理表を作成する。

1.5　被験者に対する既知及び可能性のあるリスクとベネフィットの要約

1.5.1　本被験機器使用により予想されるリスク

BTM1 の埋植及び BTM1 により作製されたバイオチューブの使用により、被験者に以下の不具合や有害事象が生じる可能性がある。なお、被験者の安全性に直接的に影響しない不具合については治験機器概要書に記載する。

(1) 不具合及び有害事象（BTM1）

- 皮下埋植中の BTM1 の変形、破損、分解等
- 皮下埋植中の BTM1 に起因する感染またはアレルギー／アナフィラキシー反応、及びそれらに関連する症状（ショック、発作、発熱、嘔気／嘔吐、痙攣等）
- 皮下埋植時、埋植中または摘出時の BTM1 の物理的損壊による負傷（出血、皮膚傷害、皮下損傷、穿孔、疼痛及び圧痛（頭部、頸部）、骨折等）

(2) 不具合及び有害事象（バイオチューブ）

- 形成不良（長さ、強度の不足等）:
  作製されたバイオチューブが、形成不良により単独でバイパス術を行うには不十分な長さであった場合、複数のバイオチューブの断片をつなぎ合わせる、または人工血管や自家静脈と吻合する等により血行再建を試みることになる可能性がある。また、使用できる断片が全く形成されなかった場合は、バイパス術自体を実施できない可能性がある。
- 移植時または移植後の変形、破損、破裂、損傷等
- 血栓形成または狭窄、閉塞による血行再建不良

(3) その他のリスク

原疾患の急速な増悪等、何らかの理由により十分な埋植期間を確保できなかった場合や治験を中止する必要が生じた場合は、BTM1 を埋植してもバイパス術を実施できないことがあると予想される。

1.5.2　本被験機器使用により予想されるベネフィット

BTM1 で作製したバイオチューブをバイパス術に用いることで、既存の下肢血行再建術では治療困難な患者の治療を行うことができる可能性がある。バイオチューブを用いたバイパス術を実施した場合に考えられるベネフィットを以下に示す。

(1) バイオチューブにより血行再建できることによるベネフィット

大切断の回避、潰瘍の創傷治癒、疼痛の緩和及び解消、歩行回復、日常生活動作の改善、認知機能の低下抑制、生命予後の改善（死亡率の低減）、対側肢切断の回避、医療コストの軽減

(2) バイオチューブが自己組織であることによるベネフィット

- 感染の原因になりにくく、万が一感染が起こっても人工物に比較して管理が容易である。

・移植後に閉塞した場合であっても、自然吸収されるため摘出する必要がない。

2. 治験の目的

本治験は、至適な伏在静脈が存在しない重症下肢虚血患者を対象とし、被験機器「BTM1」を用いて患者の皮下で作成したバイオチューブを用いて膝下の下腿動脈あるいは足部動脈へのバイパス術を施行することで、その安全性と有効性を評価することを目的とする。

3. 治験のデザイン

3.1　治験の種類及びデザイン

3.1.1　治験の種類

探索的医師主導治験

3.1.2　治験のデザイン

非盲検、非対照、多施設共同治験

＜設定根拠＞

本治験は、至適な伏在静脈が存在しない重症下肢虚血患者に対して、比較対象となる医療機器は存在せず、自家静脈の代わりに BTM1 により被験者皮下で作製されたバイオチューブを下腿動脈あるいは足部動脈へのバイパス術に用いることの有効性及び安全性を探索的に検討することを目的としているため、非盲検、非対照試験として実施することとした。

3.2　評価項目

(1) 有効性評価項目

1) 主要評価項目

被験機器によるバイオチューブの形成能

2) 副次評価項目

①被験機器の埋植・摘出に関する手技的成功
②被験機器によって形成されたバイオチューブを用いた末梢側吻合を含むバイパス術の手技的成功
③移植後のバイオチューブの 12 週時点の開存
④症状の改善（虚血性疼痛の改善、創傷の改善）
⑤大切断の回避
⑥被験機器埋植中及びバイオチューブ移植後の追加治療の実施率
⑦被験機器埋植中及びバイオチューブ移植後の QOL
⑧バイパス術後のバイパスグラフト径

＜設定根拠＞

まず被験機器を用いてバイオチューブが形成されて、それがバイパス術に使用可能であり、さらに代替血管として機能することを確認する必要がある。次に実際に治療効果があって足切断を回避できるのか、症状の改善を調べる必要がある。静脈を用いたバイパス術では通常 12 週で症状の改善が得られているため、12 週までの経過観察が必要である。

(2) 安全性評価項目

①被験機器の皮下埋植期間中の被験機器との関連を否定できない炎症、腫瘍形成、死亡の発生
②移植したバイオチューブの生体適合性（炎症、拒絶、腫瘍形成等）
③移植したバイオチューブの破裂
④移植したバイオチューブとの関連を否定できない死亡の発生
⑤バイオチューブの移植後 12 週までの死亡の発生
⑥その他、有害事象及び不具合の発生

＜設定根拠＞

治療機器は体内に埋め込まれるため、体に悪影響を及ぼさないか慎重に調べる必要がある。また、形成されるバイオチューブは自己組織であるが、皮下で形成された組織を血管として別の部位で使用するため、不具合を起こさず安全に生着し、血管として機能することを確認する必要がある。

3.3　被験者の治験参加期間

各被験者の治験参加期間は、同意取得日から後観察期間終了時（バイオチューブ移植後 12 週）までとする。

ただし、有害事象に伴う追跡期間を除く。

3.4　症例登録の制限

本治験は BTM1 をヒトに使用する最初の臨床試験であることに鑑み、被験者の安全性確保の観点から、以下の方策により症例登録を制限する（「図 3.4 被験者の組み入れ規定」参照）。

① 移植 1 カ月経過した時点において、効果安全性評価委員会によって治験継続を判断する「症例登録の制限」を設ける。
② 全ての実施医療機関において、1 例成功するまで症例登録の制限は解除できない。
③ 最初の実施医療機関で 3 例目までに 1 例成功するまでは、他の実施医療機関での症例登録はできない。
④ 各実施医療機関において、または全実施医療機関を通じて、3 例連続して不成功であれば、治験は終了する。
⑤ 全症例において、移植後 1 カ月時に効果安全性評価委員会を開催する。
⑥ 何らかの問題が発生した場合には、その都度効果安全性評価委員会を開催する。

図 3.4 被験者の組み入れ規定

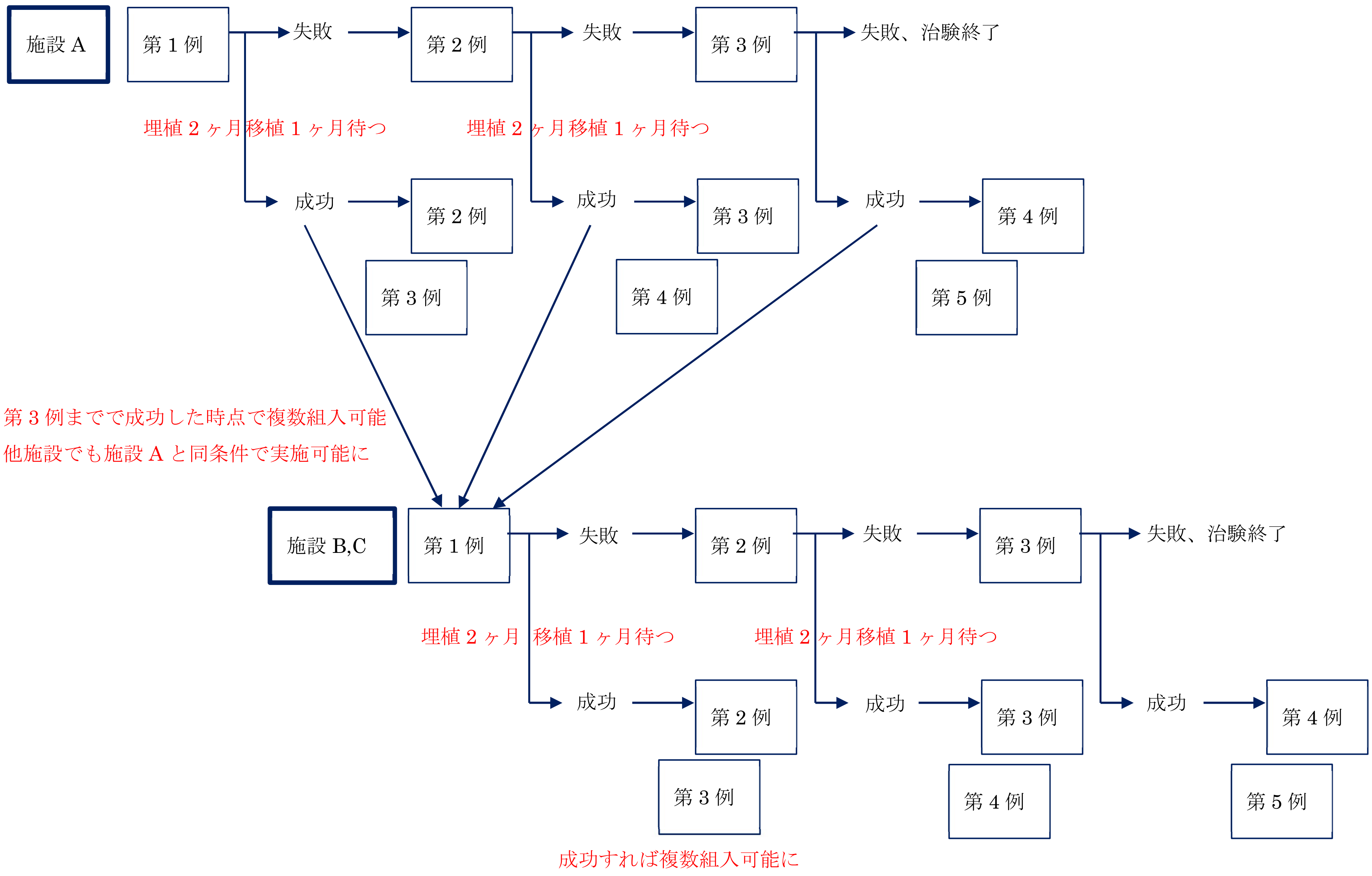

3.5 治験実施予定期間

2022 年 8 月～2026 年 5 月（登録期間：2022 年 8 月～2025 年 11 月）

4. 被験者の選択・除外、中止基準

治験参加の同意が本人から文書で得られ、登録時に「4.2 選択基準」すべてを満たし、「4.3 除外基準」のいずれにも抵触しない患者を本治験の被験者とする。

4.1 対象疾患

膝下の下腿動脈あるいは足部動脈へ末梢吻合するバイパス術を必要とするが、至適な自家静脈が存在しない重症下肢虚血の患者

4.2 選択基準

(1) 治験参加に関して、本人から文書による同意が得られた患者
(2) 同意取得時の年齢が 18 歳以上である患者
(3) 包括的高度慢性下肢虚血（Chronic Limb Threatening Ischemia; CLTI）の重症度 WIfI 分類（参考文献 15）において虚血（Ischemia）grade 2 または 3（ABI <0.6, SPP <40 mmHg）を満たす重症下肢虚血患者
(4) 下肢動脈バイパス術が推奨される患者のうち、以下のいずれかに該当する患者
  a. Global Vascular Guideline（GVG）（参考文献 8）の GLASS（Global Anatomic Staging System）分類において stage III の虚血肢で、WIfI 分類 clinical stage 2、3 または 4 に該当する
  b. GLASS stage II の虚血肢で、WIfI 分類 clinical stage 3 または 4 に該当する
  c. GLASS stage I または II の虚血肢で、血管内治療を行っても十分な血流を得られない（創傷の治癒につながらず臨床症状の改善が得られない臨床的不成功を含む）
(5) バイパス術に必要な長さ及び径（3 mm 以上）（参考文献 16 - 18）を満たし、瘤化等の異常所見がない至適な上肢または下肢静脈（透析患者の場合、下肢静脈）が存在しない患者
(6) 膝下の下腿動脈あるいは足部動脈へのバイパスの末梢吻合を必要とする患者
(7) 同意取得時点において 12 ヶ月以上の生存及びバイパス術後 12 週間の経過観察が可能であると判断される患者

＜設定根拠＞
(1)、(2)、(7) 倫理的及び安全性の観点から適切な対象患者を選択するため
(3) 血行再建が必要とされる患者を選択するため
(4) 治療法選択において、バイパス術が最も良い適応となる患者を選択するため
(5) バイパスグラフトとして適切な自家静脈が存在しない患者を選択するため
(6) 既存の人工血管ではバイパスグラフトとして代用できない領域であるため

4.3 除外基準

(1) 直ちに血行再建術が必要な状態である等の理由により、バイオチューブ形成に必要な被験機器の埋植期間の確保が困難な全身状態である患者

(2) 重度の低栄養（CONUT スコア 8～12）（参考文献 19）や重症の合併症等により、手術認容が困難と判断される全身状態である患者
(3) 皮膚の状態が不良である、過去に皮下埋植物の露出歴がある等の理由により、被験機器の埋植部位を2ヶ所以上確保できない可能性がある患者
(4) 登録前 30 日以内に侵襲を伴う外科手術を受けている患者（足の創傷に対する処置を行った場合は除く）
(5) バイパス術に必要な末梢側の標的動脈が存在しない、あるいは末梢側の吻合予定部位に血管内治療施行歴がある患者
(6) バイパス術の中枢側の吻合予定部位より近位に動脈の閉塞が認められる患者
(7) バイパス術の末梢側の吻合予定部位より遠位の血行が確認できない患者（側副血行が確認できる場合を除く）
(8) 中足部より近位での下肢切断が施行されている患者
(9) 悪性腫瘍の既往や合併のある患者（治療後 5 年間以上再発を認めない、もしくは新規の発症がないものを除く）
(10) 自己免疫疾患の合併や移植後等のため免疫抑制剤を使用中の患者
(11) ステンレス鋼またはポリオレフィン樹脂にアレルギーの既往がある患者
(12) 妊娠している、または妊娠の可能性がある患者
(13) 他の治験または介入のある臨床研究に参加中もしくは参加予定である患者
(14) その他、病状等や安全上の理由により、治験責任医師または治験分担医師が本治験の対象として不適切であると判断した患者

＜設定根拠＞
(1)、(2)、(4)、(9)～(12) 被験者の安全性確保のため
(3)、(5)～(8) 被験機器の有効性及び安全性を適切に評価するため
(13)、(14) 倫理的及び安全に治験を実施するため

4.4 中止基準

(1) 治験責任医師または治験分担医師が治験継続困難と判断する有害事象の発現や原疾患の増悪がみられた場合
(2) バイパス術に使用できるバイオチューブが、断片状のものも含めて全く形成されず、それ以降の治験継続が不可能となった場合
(3) 被験者が治験参加の同意を撤回した場合
(4) 登録後に被験者が適格性を満たしていないことが判明した場合
(5) 被験機器埋植後かつバイオチューブ移植前に被験者の妊娠が判明した場合
(6) 治験実施計画書からの重大な逸脱が判明した場合
(7) BTM1 植込み後 24 週経過後もバイパス術が不要だった場合、BTM1 は全て取り出し、治験は中止とするが、バイオチューブの形成能は評価する。
(8) その他、治験責任医師または治験分担医師が本治験を中止すべきと判断した場合

＜設定根拠＞
いずれも倫理的観点、被験者の安全性担保の観点から設定した。

5. 併用薬、併用療法及び検査に関する規定

5.1 併用薬

以下の薬剤については、規定する期間内における併用を禁止する。

【同意取得後から治験期間を通して使用を禁止する薬剤】
・悪性腫瘍に対する化学療法を目的とした薬剤
・免疫抑制作用を持つ薬剤（全身作用を目的としないものを除く）

【バイオチューブ移植の前に使用を禁止する薬剤】
ヘパリンを除く抗凝固薬について、以下に規定する期間の使用を禁止する。
・ワルファリン：移植 3 日前から移植前日まで
・直接作用型経口抗凝固薬：移植前日
（ダビガトラン、リバーロキサバン、アピキサバン、エドキサバン）

5.2 併用療法

バイオチューブ移植後は、血栓の形成を防止するため、適切な抗血栓療法を実施する。

原則として、バイオチューブ移植後 7 日間はヘパリンを投与し、その後、経口の抗凝固薬に切り替え、移植 12 週後まで投与を継続する。ヘパリン投与中は、APTT が術前（Visit 7）の 1.5～2 倍の範囲内となるようコントロールすること。抗血小板薬は、クロピトグレルまたはシロスタゾールを単独投与し、アスピリンとの抗血小板薬 2 剤併用療法は実施しない。

その他の併用療法については特に規定及び制限を行わない。

5.3 検査の制限

BTM1 の埋植期間（Visit2～Visit6）は、MRI 検査の実施は不可とする。なお、BTM1 の摘出後 MRI 検査の実施は、制限を行わない。

6. 治験の手順

6.1 同意取得

(1) 治験責任医師または治験分担医師は被験者の選定にあたり、当該患者の健康状態、症状、年齢、性別、同意能力、他の治験等への参加の有無等を考慮し、当該患者を治験の対象とすることの適否を慎重に検討する。
(2) 治験責任医師または治験分担医師は、本治験の対象として適切と判断した患者に対し、本治験の説明を十分に行い、患者本人より文書による同意を取得する（詳細は「11.2 被験者への説明と同意の取得」及び「11.3 同意取得の方法」参照）。

6.2　適格性確認

(1)　治験責任医師または治験分担医師は、本治験参加への同意を取得した被験者について被験者スクリーニング名簿を作成し、被験者識別コードを付与する。

(2)　治験責任医師または治験分担医師は、同意取得後から登録時までに必要な検査及び調査を実施し、選択基準及び除外基準に基づき、本治験参加の適格性を判定する（「7.5 観察・検査スケジュール」参照）。

なお、適格性確認においては、治験責任医師または治験分担医師による判断の他に、適切な専門性を持つ第三者（血管治療、創傷管理の専門スタッフ等）の意見を聴くことを必須とする。治験責任医師は、各実施医療機関内において、そのための体制を構築すること。

6.3　被験者登録

(1)　治験責任医師または治験分担医師は、「6.1 同意取得」の手順により同意を取得した被験者に対し、「6.2 適格性確認」の手順により適格性確認を実施する。

(2)　治験責任医師または治験分担医師は、本治験に適格であることが確認された被験者について、別途定める手順に従い、本治験に登録する。治験責任医師または治験分担医師は登録が行われたことを確認後、被験機器を埋植する。

6.4　被験機器の使用

6.4.1　治験の手順

本治験に登録された被験者に対し、治験責任医師または治験分担医師が治験治療を行う。

「6.5 観察・検査スケジュール」及び「6.5.1 観察・検査・調査内容」で定めた調査及び検査等を行い、被験機器の安全性の確認と有効性の評価を行う。

6.4.2　被験機器 BTM1 の使用手順

(1) バイオチューブの作製方法

1)　胸部、腹部、臀部または大腿部の皮膚を切開し、真皮層下を剥離することで皮下ポケットを作製する。被験者毎の BTM1 の埋植個数及び部位については、以下の①～③に従い決定する。

① 埋植個数は最小で 2 個、最大で 4 個とする。

② 腹部に 1 個埋植することを必須とする。

③ ②を除く残りの埋植個数（1～3 個）及びその埋植部位（胸部、腹部、臀部または大腿部）については、被験者の体格及び希望ならびに治験責任医師または治験分担医師の医学的判断（埋植のための十分なスペースを確保できる、当該部位の皮膚異常等がない等）により決定する。

2)　作製した皮下ポケットにサイザーを挿入し、埋植に十分な大きさのスペースが確保されていることを確認する。

3) BTM1 を包装袋から無菌的に取り出し、外観上、変形・異常等がないことを確認する。使用するBTM1 のサイズは、被験者の体格等に応じて治験責任医師または治験分担医師の判断により選択する。
4) BTM1 を皮下ポケットに挿入し、皮下に留置する。
5) 皮下出血が生じた場合には、電気メス等で完全に止血する。
6) 皮下にブレイクドレーンを挿入し固定する。
7) 皮内縫合し、BTM1 を皮下内で固定し、閉創する。
8) ブレイクドレーンを通して BTM1 内の空気の吸引を開始する。
9) 埋植 2～7 日後、埋植部位のエコー検査及び目視により出血や滲出液が治まったことを確認しブレイクドレーンを抜去する。なお、ブレイクドレーンの抜去から約 1 週間後にも埋植部位をエコー検査により確認する。エコー検査により BTM1 の周囲で血液や滲出液の貯留が認められた場合は穿刺により除去する。
10) 埋植後 4～24 週間（原則として 8 週間以上）、BTM1 を静置する。
11) 再度、皮膚を切開し、止血しながら皮下組織を剥離して BTM1 を取り出す。
12) BTM1 の外周の結合組織を除去した後、外殻を取り外し、形成されたバイオチューブを中芯とともに取り出す。
13) 中芯からバイオチューブを抜き取り、生理食塩液で洗浄する。
14) 直線化棒をバイオチューブ内腔に挿入し、浸漬皿内でアルコール（70%）に 30 分間浸漬する。
15) 生理食塩液にて再度バイオチューブを洗浄し、外観上、破れ・欠損・肉厚の顕著な偏り等がないことを目視で確認する。
16) ヘパリン加生理食塩水中で移植に使用するまで保管する。
17) 自家静脈グラフトでのバイパス術の場合と同様に、バイオチューブの内部にシリンジで生理食塩水を負荷し、漏れがないことを確認するとともに、圧モニターを使用して耐圧性（200 mmHg の圧負荷条件下における膨化及び破裂の有無）を確認する。また、引張試験機を使用して最大強度を測定し、基準強度（5 N）を満たしていることを確認する。

上記 1)～17)の手順により作製されたバイオチューブを、自家静脈グラフトと同様に代替血管として被験者の下肢に移植する。

被験者の全身状態等の理由により、被験機器摘出とバイオチューブ移植を同日中に実施できない場合、作製されたバイオチューブの一時的な保存を許容する。一時保存する場合、無菌状態を保ったまま 10%アルコールに浸して密封の上、室温で保存する（作製されたバイオチューブが断片状である場合はそのまま保存）。この場合、一時保存期間は最大 4 週間とし、被験機器摘出から 4 週以内にバイオチューブ移植を実施すること。また、治験責任医師は、一時保存中に紛失や取違え等が起こらないよう、保存場所の確保及び保存手順の作成等、治験開始前にあらかじめ適切に準備しておくこと。

＜設定根拠＞

非臨床試験の結果により、バイオチューブは室温で 6 ヶ月間形状や強度に大きな変化はなかったことが確認できている。以上より、一時的に保存可能であることは確認できているものの、病状の悪化を防

ぐため、できるだけ早く、遅くとも被験機器摘出から 4 週間以内にバイオチューブを移植するのが望ましいと考える。したがって、一時保存の期間を最大 4 週間（室温）とした。

(2) バイオチューブの強度試験及び病理組織評価

1) 移植に使用するバイオチューブについて、強度試験及び病理組織評価用の試料を採取する。採取する試料の長さの目安は 0.5～2 cm とするが、作製されたバイオチューブの長さ等に応じて、別途定める手順により適宜調整することを許容する。

2) 1)で保存した試料を用いて、別途定める手順により強度試験及び病理組織評価を実施する（作製されたバイオチューブが断片状である場合は、各断片について実施）。なお、強度試験については、(1)の 16)に定めるとおり、原則としてバイオチューブ移植前に実施する。

＜設定根拠＞

バイオチューブが移植に使用できるかの評価判断として、強度を調べる必要がある。また、形成されたバイオチューブの組織の成分を調べ、部位や被験者による違いを把握する必要がある。以上より、強度試験と病理組織評価を実施することとした。

(3) バイオチューブの品質評価の実施時期

(1)及び(2)の手順によりバイオチューブの品質評価を実施する場合は、以下に示す時期に実施する。なお、作製されたバイオチューブが断片状である場合は、原則として各断片について実施する。

1) バイオチューブの一時保存を行わない場合（BTM1 摘出とバイオチューブ移植を同日に実施する）

実施時期		実施項目	バイオチューブの状態		
			つなぎ合わせずに使用可能	断片状	
				つなぐ前（断片毎）	つないだ後
Visit 6	BTM1 摘出直後	外観	○	○	
		耐圧性	○	○	
		強度	○	○	
		病理組織評価用試料保存	○	○	
Visit 7	バイオチューブ移植前	外観			
		耐圧性			○※1
		強度			
		病理組織評価用試料保存			

2) バイオチューブを一時保存する場合（BTM1 摘出とバイオチューブ移植を別日に実施する）

実施時期		実施項目	バイオチューブの状態		
			つなぎ合わせずに使用可能	断片状	
				つなぐ前（断片毎）	つないだ後
Visit 6	BTM1 摘出直後	外観	○	○	
		耐圧性	○	○	
		強度	○	○	
		病理組織評価用試料保存	○	○	
一時保存（必要時のみ、最大 4 週）			○	○	
Visit 7	バイオチューブ移植前	外観	○	○	
		耐圧性	○	○	○[※1]
		強度	○	○	
		病理組織評価用試料保存	△[※2]	△[※2]	

※1：バイオチューブ自体の品質評価を目的とするものではないが、接合部分からの漏れの有無等を確認するため、耐圧性検査と同様の方法で実施する。

※2：可能な場合に実施する。試料の量が不足する場合は実施しない。

(4) 移植に至らなかったバイオチューブの評価

バイオチューブ移植前に何らかの理由で治験中止となり、埋植した被験機器を摘出した場合は、(1)及び(2)の手順に準じ、その時点におけるバイオチューブの外観、強度及び病理組織の評価を行う。

＜設定根拠＞

本治験の目的は、バイオチューブ形成能の評価を主としており、移植に至らなかった場合でも被験機器が適切に摘出された場合には、バイオチューブ形成能の評価が可能であるため。

(5) 移植に用いられることなく余ったバイオチューブの取り扱い

移植後余剰となったもしくは何らかの理由で移植に至らず使用されなかったバイオチューブは他の研究目的で使用することを想定し 10%アルコールに浸して保存する。

(6) 被験機器埋植の中止

1) 被験機器の埋植部位に局所的な有害事象等（感染、炎症、被験機器の露出等）が発現した場合、当該部位への埋植は中止とし、適切に摘出及び処置を行う。なお、他の埋植部位に異常が認められない場合は、残りの被験機器の埋植を継続する。
2) 全身状態の悪化を伴う有害事象等により、治験実施計画書に定めるスケジュールを遵守した被験機器の埋植の継続またはバイオチューブ移植の実施が困難な状態となった場合は、原則として被験機器を全て摘出した上で治験中止とする。

6.4.3 バイオチューブ移植（バイパス術）に関する事項

(1) バイオチューブ移植（バイパス術）の実施

1) バイオチューブのみの使用

被験機器により形成されたバイオチューブが、長さ、厚さ及び強度等の観点からバイパス術に使用できる品質を満たしていた場合、バイオチューブのみをバイパスグラフトとしたバイパス術を実施する。なお、断片状のバイオチューブをつなぎ合わせることにより、バイオチューブのみで必要な長さを確保できる場合を含む。

2) バイオチューブと自家静脈または人工血管との併用

被験機器により形成されたバイオチューブが、長さ、厚さ及び強度等の観点から、バイオチューブのみではバイパス術に使用できる品質を満たさない場合（断片状のバイオチューブをつなぎ合わせても必要な長さを確保できない場合を含む)、自家静脈または人工血管と併用してバイパス術を実施する。

(2) バイオチューブ移植（バイパス術）の延期または中止

以下の場合は、バイオチューブ移植（バイパス術）を中止とし、治験を中止する。なお、中止する場合であっても、「6.4.2 (4) 移植に至らなかったバイオチューブの評価」で定める手順に従い、バイオチューブの評価を実施する。

・使用可能な品質のバイオチューブが全く形成されなかった場合。
・創部感染で壊死範囲が拡大した等の理由により標的下肢の大切断が必要となった場合。
・有害事象の発現等により、バイオチューブ移植の実施が困難な全身状態となった場合。ただし、治験実施計画書で規定した許容期間内にバイオチューブ移植の実施が検討できると治験責任医師または治験分担医師が判断する場合に限り、中止ではなく一時的に延期をすることを許容する。

6.4.4 被験機器の埋植期間及び後観察期間

被験機器埋植期間：4～24 週間（原則として 8 週間以上）

後観察期間：バイオチューブ移植後 12 週まで

なお、この他に同意取得から登録までの前観察期間（最大 8 日）と、有害事象に伴う追跡期間（追跡が必要な有害事象が発現した場合）を含む。

＜設定根拠＞

埋植期間が長いほどバイオチューブの組織形成は進むが、下肢虚血の過度の症状悪化を避けるため、できるだけ短期間でバイパス術を行うことが望まれる。そのため、症状との兼ね合いにはなるが、バイオチューブ形成の確実性を期すために埋植期間は原則 2 ヶ月程度（8 週間）と設定した。なお、非臨床試験においては、バイオチューブは 1 ヶ月の埋植期間で形成できることを確認していることから、埋植後に著しい虚血の進行がみられた場合には、4 週以上 8 週未満の埋植期間であっても摘出及びバイパス術の実施を許容できると考え、最短の埋植期間を 4 週間と設定した。

　また、自家静脈を用いたバイパス術を行う場合、通常、移植後 3 ヶ月程度で症状が改善していること、非臨床試験の結果から、概ね移植後 3 ヶ月程度経過する頃には移植したバイオチューブは自己組織に置き換わっていると考えられることから、バイオチューブ移植後の後観察期間を 12 週と設定した。

6.5　観察・検査スケジュール

下表 6.5 に従い、観察・検査を行う。

表 6.5　観察・検査スケジュール

実施時期 ／ 観察項目	前観察期			被験機器埋植期						後観察期								中止時	
	Visit（V）1			V2		V3	V4	V5 ※4	V6 ※5	V7	V8	V9	V10	V11	V12	V13		被験機器埋植期間中	後観察期間中
	同意取得	適格性確認	被験者登録	被験機器埋植		ブレイクドレーン抜去	ドレーン抜去後1週	被験機器埋植後6週	治験機器摘出	バイオチューブ移植	移植後1日	移植後3日	移植後1週	移植後4週	移植後8週	移植後12週			
許容範囲	V2 −8日					V2＋ 2〜7日	V3＋ 5〜8日	V2＋ 5〜7週	V2＋ 4〜24週	V6＋ 0日〜4週			±2日	±7日	±7日	±7日		+7日	+7日
同意取得	●																		
被験者背景（身長・体重以外）		●																	
被験者登録			●																
身長・体重		●※2																	
自他覚所見		●		●※3				●	●	●※6	●	●	●	●	●	●		●	●
バイタルサイン		●※2		●				●	●	●※6	●	●	●	●	●	●		●	●
血液検査		●※2		●※3				●	●	●※6	●	●	●	●	●	●		●	●
下肢動脈エコー検査		●※2						●※4					●	●	●	●		●	●
上肢・下肢静脈エコー検査		●※2																	
下肢 CT 血管造影検査 ※1		▲※2											▲			△			
下肢動脈造影検査 ※1		▲※2											▲			△			
下肢 X 線検査		●※2											●	●	●	●		●	●
ABI 検査		●※2											●	●	●	●		●	●
SPP 検査		●※2											●	●	●	●		●	●
虚血状態評価		●											●	●	●	●		●	●
下肢 MRI 検査		○※2																	
第三者による適格性確認		●																	
被験機器埋植					●														
被験機器埋植部位エコー検査						●	●												
ブレイクドレーン抜去						●													
アンケート調査								●※4								●		●	●
被験機器摘出									●									●	
バイオチューブ品質評価 ※7									●	●※7									
バイオチューブ試料保存									●	○※8								●	
バイオチューブ移植										●									
併用薬・併用療法		←―	―	―	―	―	―	―	―	―	―	―	―	―	―	―→		●	●
有害事象					←―	―	―	―	―	―	―	―	―	―	―	―→		●	●
不具合 ※9					←―	―	―	―	―	―	―	―	―	―	―	―→		●	

●：必須　　○：任意で実施または可能な場合に実施　　▲：両方またはいずれかを実施　　△：両方またはいずれかを可能な場合に実施

※1：両方またはいずれか一方の検査を実施する。
※2：同一の検査（調査）項目の結果が複数存在する場合は、直近のものを採用する。許容された期間内に治験実施医療機関で実施した検査（調査）の結果がある場合は、同意取得前に得られた結果であっても使用可能とする。なお、第三者による適格性確認の前に実施すること。
※3：適格性確認時と状態が変わっていない等の理由で治験責任医師または治験分担医師が検査（調査）不要と判断する場合は必須としない。
※4：被験機器埋植期間を短縮せざるを得ず、Visit 5 をスキップする（規定された Visit 5 の実施時期より前に被験機器摘出（Visit 6）を実施する）ことになった場合は、下肢動脈エコー検査及びアンケート調査を被験機器摘出より前に実施する。
※5：埋植後に著しい虚血の進行がみられた等、被験者の安全性確保のためにやむを得ない理由がない限り、原則として、Visit 2 から 8 週間以上経過後に実施する。
※6：Visit 6（被験機器摘出）と同日～7 日以内に Visit 7 を実施する場合は、必須としない。Visit 6 と Visit 7 の間が 8 日以上空く場合、Visit 7 のバイオチューブ移植前に実施する。ただし、Visit 6 と Visit 7 を同日中に実施しない理由が被験者の病状悪化であった場合は、Visit 6 と Visit 7 の間の日数によらず、可能な限り実施すること。
※7：バイオチューブの外観、耐圧性、強度を評価する。Visit 6（被験機器摘出）と同日に Visit 7 を実施する場合は、被験機器摘出からバイオチューブ移植前までの間に一度実施することで差し支えないが、Visit 6 と同日に Visit 7 を実施せずバイオチューブを一時保存する場合は、移植術の実施前に、一時保存後のバイオチューブについて再度品質評価を実施すること。
※8：バイオチューブの病理組織評価を行うための試料を保存する。Visit 6（被験機器摘出）と同日に Visit 7 を実施せずバイオチューブを一時保存する場合で、かつ試料の量が十分な場合に、一時保存後のバイオチューブも病理組織評価用に保存する。
※9：バイオチューブは被験機器ではないが、本治験の特性に鑑み、被験機器摘出後もバイオチューブの不具合について調査を行う。

6.5.1　観察・検査・調査内容

治験責任医師または治験分担医師は、以下の項目を調査し、記録する。

(1) 被験者背景

【調査項目】被験者識別コード、同意取得日、性別、生年月日、年齢、身長、体重、原疾患（下肢虚血の原因となった疾患）、バイオチューブ移植の標的となる下肢（以下、「標的下肢」）、標的下肢の評価、既往歴（適格性確認時点で完治しているもの）・合併症（適格性確認時点で完治していないもの）の有無及びその内容、妊娠検査、栄養状態、被験機器埋植予定部位の状況

【調査時期】適格性確認時

【調査方法】問診及び検査により調査を行う。

身長については、被験者が立位姿勢を保持できない等の理由により身長計を使用した測定が困難な場合、メジャー等を利用した簡易的な測定を許容する。

被験者が妊娠可能な女性であった場合は妊娠検査を実施し、妊娠していないことを確認する（検査方法は不問）。

【CRF 記載内容】被験者識別コード、同意取得日、性別、生年月日、年齢（同意取得時）、身長の測定日、身長 [cm]（整数：小数点以下第 1 位を四捨五入）、体重の測定日、体重 [kg]（小数点以下第 1 位まで：小数点以下第 2 位を四捨五入）、原疾患、標的下肢（右または左）、標的下肢の評価（血管内治療を含む血行再建術施行歴の有無及びその内容、切断の有無及びその内容、バイパス術に必要なバイオチューブの長さ）、既往歴・合併症の有無及び疾患名、妊娠検査（実施の有無、検査日、判定結果、実施なしの理由）、栄養状態（CONUT スコア）、被験機器埋植予定部位の状況（予定部位、埋植予定部位、皮膚異常を含む、異常の内容）

(2) 自他覚所見

【調査項目】自他覚所見（重症下肢虚血に伴う所見：疼痛、しびれ、熱感、圧痛、腫脹、創傷、滲出液、潰瘍・びらん形成、発熱、及びその他特筆すべき所見）

【調査時期】適格性確認時、Visit 2（被験機器埋植前）、Visit 5、Visit 6（被験機器摘出前）、Visit 7（バイオチューブ移植前）、Visit 8、Visit 9、Visit 10、Visit 11、Visit 12、Visit 13、中止時

【調査方法】診察を行い確認する。また、原疾患に起因する創傷がある場合、写真を撮影する。

【CRF 記載内容】重症下肢虚血に伴う自他覚所見及び異常の有無：疼痛、しびれ、熱感、圧痛、腫脹、創傷、滲出液、潰瘍・びらん形成、発熱（24 時間に 37.5℃以上）、及びその他特筆すべき所見）の有無及びその内容【創傷については大きさ（縦・横）を含む)】、創傷部位写真の有無、撮影日、創傷部位写真（創傷がある場合）

(3) バイタルサイン

【調査項目】体温、血圧、脈拍数

【調査時期】適格性確認時、Visit 2（被験機器埋植前）、Visit 5、Visit 6（被験機器摘出前）、Visit 7（バイオチューブ移植前）、Visit 8、Visit 9、Visit 10、Visit 11、Visit 12、Visit 13、中止時

【CRF 記載内容】体温 [ºC]（小数点以下第 1 位まで）、血圧（収縮期及び拡張期）[mm Hg]（整数）、脈拍数［回／分］

(4) 血液検査（血液学的検査、血液生化学検査、血液凝固検査）

【調査項目】①血液学的検査：白血球数（分画）、赤血球数、血色素量、ヘマトクリット値、血小板数
総リンパ球数（適格性確認時のみ）
②血液生化学検査：総タンパク、アルブミン、空腹時血糖、AST（GOT）、ALT（GPT）、中性脂肪、LDL コレステロール、LDH、BUN、クレアチニン、Na、K、Cl、CRP
総コレステロール（適格性確認時のみ）
③血液凝固検査：PT、PT-INR、APTT

【調査時期】適格性確認時、Visit 2（被験機器埋植前）、Visit 5、Visit 6（被験機器摘出前）、Visit 7（バイオチューブ移植前）、Visit 8、Visit 9、Visit 10、Visit 11、Visit 12、Visit 13、中止時

【CRF 記載内容】各検査値

(5) 下肢動脈エコー検査

【調査項目】下肢動脈エコー検査

【調査時期】適格性確認時、Visit 5、Visit 10、Visit 11、Visit 12、Visit 13、中止時

【CRF 記載内容】標的下肢の動脈エコー検査所見【標的下肢の動脈の梗塞・狭窄の有無、標的下肢の動脈の新規梗塞・狭窄の有無（所見、異常所見の有無）】、移植後のバイパスグラフトの径・狭窄の有無（所見、異常所見の有無）

(6) 上肢・下肢静脈エコー検査

【調査項目】上肢及び下肢静脈エコー検査

【調査時期】適格性確認時（透析患者の場合、上肢の検査は不要）

【CRF 記載内容】上肢及び下肢の静脈エコー検査所見（静脈の部位、長さ及び静脈瘤の有無、動脈バイパス術に使用できる自家静脈の有無）

(7) 下肢 CT 血管造影検査、下肢動脈造影検査

【調査項目】下肢 CT 血管造影検査、下肢動脈造影検査

【調査時期】適格性確認時、Visit 10、Visit 13（可能な場合に実施）

【調査方法】標的下肢に対し、下肢 CT 血管造影検査、下肢動脈造影検査の両方またはいずれか一方を実施する。

【CRF 記載内容】検査方法、標的下肢の虚血に関する所見の有無及び所見

(8) 下肢 X 線検査

【調査項目】下肢 X 線検査

【調査時期】適格性確認時、Visit 10、Visit 11、Visit 12、Visit 13、中止時
【CRF 記載内容】標的下肢の X 線検査所見の有無及び所見、骨髄炎の有無

(9) ABI（足関節上腕血圧比）検査
【調査項目】ABI 検査
【調査時期】適格性確認時、Visit 10、Visit 11、Visit 12、Visit 13、中止時
【CRF 記載内容】ABI 測定値（右、左）

(10) SPP（皮膚灌流圧）検査
【調査項目】SPP 検査
【調査時期】適格性確認時、Visit 10、Visit 11、Visit 12、Visit 13、中止時
【CRF 記載内容】標的下肢の SPP [mm Hg]（整数）

(11) 虚血状態評価
【調査項目】WIfI 分類及び GLASS 分類の各分類におけるスコア
【調査時期】WIfI 分類：適格性確認時、Visit 10、Visit 11、Visit 12、Visit 13、中止時
GLASS 分類：適格性確認時
【CRF 記載内容】標的下肢の WIfI 分類及び GLASS 分類の各分類におけるスコア

(12) 下肢 MRI 検査
【調査項目】下肢 MRI 検査
【調査時期】適格性確認時（標的下肢に骨髄炎の合併が疑われ、かつ検査可能な場合に実施）
【CRF 記載内容】標的下肢の MRI 検査の有無及び所見、骨髄炎の有無

(13) 第三者による適格性確認
【調査項目】第三者による適格性確認の実施状況
【調査時期】適格性確認時
【CRF 記載内容】第三者による適格性確認の実施の有無及びその内容（確認日及び確認者を含む）

(14) 被験機器埋植
【調査項目】被験機器の埋植手順の遵守状況
【調査時期】Visit 2
【調査方法】「6.4.2　被験機器 BTM1 の使用手順」の遵守状況を調査する。
【CRF 記載内容】被験機器の埋植手順の遵守状況（埋植個数及び部位（左右も含む）、サイズ）

(15) 被験機器埋植部位エコー検査（ブレイクドレーン抜去を含む）
【調査項目】被験機器埋植部位エコー検査
【調査時期】Visit 3（ブレイクドレーン抜去前）、Visit 4

【調査方法】ブレイクドレーンの抜去前及び抜去後約 1 週後にエコー検査を行い、被験機器埋植部位の血液または滲出液の貯留の有無を確認する。

【CRF 記載内容】被験機器埋植部位のエコー検査所見（血液または滲出液の貯留の有無、所見のあった BTM1 の番号、ブレイクドレーン抜去日を含む）

(16) アンケート調査

【調査項目】被験機器埋植に関する内容及びバイオチューブ移植後の QOL

【調査時期】Visit 5、Visit 13、中止時

【調査方法】被験機器摘出後及びバイオチューブ移植 12 週後に被験者に対しアンケート形式で調査する。

【CRF 記載内容】被験機器埋植前（被験機器を埋め込む治療に対する不安感及び期待感、自分の体で自分の血管を作ることについて、その他意見）、被験機器埋植後（埋め込み時の違和感や不安感、痛みやかゆみなどの苦痛の有無、日常生活への影響及び不自由、埋め込み前の想像との違い、埋め込み期間中の機器に対する気持ちの変化、取り出し後の違和感や不安感、その他意見）、移植後（移植部の状態、移植部の痛みやかゆみ、創傷の痛み及び歩行の改善状況、日常生活への影響、その他意見）

(17) 被験機器摘出

【調査項目】被験機器の摘出手順の遵守状況

【調査時期】Visit 6

【調査方法】「6.4.2　被験機器 BTM1 の使用手順」の遵守状況を調査する。

【CRF 記載内容】被験機器の摘出手順の遵守状況（皮下血腫の形成、皮下滲出液の貯留、過度の癒着、BTM1 の感染・サビつき・分解・破損・内部の中芯の変質及び破損）

(18) バイオチューブの品質評価（バイオチューブ病理組織評価用試料保存を含む）

【調査項目】バイオチューブの品質評価及び病理組織評価用試料保存に関する情報

【調査時期】Visit 6、Visit 7（必要な場合に実施）（「6.4.2　被験機器 BTM1 の使用手順」参照）

Visit 7 については、Visit 6 と同日中にバイオチューブ移植を実施せずバイオチューブを一時保存した場合に、バイオチューブ移植直前に実施する。（病理組織評価用試料保存に関しては、試料の量が十分である場合に実施）

【CRF 記載内容】合計の長さ、長さ、破れや穴の有無（ありの場合、長さ）、厚さ・極端に薄い部分の有無（ありの場合、長さ）、不均一部の有無（ありの場合、長さ）、漏れの有無、引張強度、耐圧性、病理組織評価用試料保存の有無、使用可能な長さ、バイオチューブの保存の有無、評価日、外観（分解、腐敗、異臭、変色など）の異常の有無、細菌検査用試料保存の有無

(19) バイオチューブ移植

【調査項目】バイオチューブ移植に関する情報

【調査時期】Visit 7
【CRF 記載内容】バイオチューブ移植実施の有無、移植日、移植延期の確認日、移植延期の移植日、使用した移植物、組み合わせ方法、移植物（バイオチューブ番号)、使用したバイオチューブの長さ、自家静脈（部位、長さ)、人口血管（製品名、長さ)、耐圧性、移植中止の理由、バイオチューブの接続の有無、接続日、接続後のバイオチューブの長さ、耐圧性の有無（なしの場合、その理由)、バイオチューブの修復の有無

(20) 併用薬・併用療法

併用薬・併用療法は、同意取得以後に使用したすべての医薬品または実施したすべての治療・処置を指す。ただし、被験機器埋植及びバイオチューブ移植は除外する。

【調査項目】対象となる医薬品、治療・処置
【調査時期】適格性確認時より Visit 13 または中止時まで。
【CRF 記載内容】併用薬の有無（併用薬がある場合はその薬剤名（商品名または一般名)、投与経路、1 回投与量、投与頻度、投与期間、投与理由)、併用療法の有無（併用療法がある場合はその療法名、施行開始日、施行終了日、治療目的)

(21) 有害事象

【調査項目】有害事象発生の有無、及び発生した場合には当該有害事象に関連する情報
【調査時期】被験機器埋植後より Visit 13 または中止時まで。
【CRF 記載内容】「7. 安全性評価」参照

(22) 不具合

【調査項目】不具合発生の有無、及び発生した場合には当該不具合に関連する情報
【調査時期】Visit 2（被験機器埋植時）より Visit 13 または中止時まで。
なお、バイオチューブは被験機器ではないが、本治験の特性に鑑み、バイオチューブの不具合についても調査を行う。バイオチューブに関する調査時期は Visit 6（被験機器摘出時）から Visit 13 または中止時までとする。
【CRF 記載内容】「7. 安全性評価」参照

6.5.2 中止後の検査・観察

登録後、何らかの理由により本治験を中止した場合はすべて中止症例として取り扱う。被験機器埋植後に中止となった場合は、「6.5 観察・検査スケジュール」及び「6.5.1 観察・調査・検査内容」に従って「中止時」の検査・観察項目を実施する。また、有害事象のため追跡が必要な場合は、原則として回復または軽快するまで経過を追跡する。

7. 安全性評価

7.1　臨床検査値

治験責任医師または治験分担医師は、被験機器埋植後に測定した検査値及び測定値を、それぞれ同意取得後に検査した結果と比較し、異常変動の有無の判定を行う。臨床検査値の異常変動を判定する際は「National Cancer Institute Common Terminology Criteria for Adverse Events（NCI CTCAE v5.0）」を参考とし、該当する項目がない場合は、表 7.1 を参考に Grade（重症度）を評価する。

臨床検査値異常が認められた場合、治験責任医師または治験分担医師は以下の項目を参考として当該異常を有害事象とするか判断する。

(1) 臨床検査値異常によって治験治療が中止された場合
(2) 臨床検査値異常を改善するため、薬物による介入や外科的介入が行われた場合
(3) 臨床症状との関連性が認められた場合
(4) 重篤な有害事象との関連が認められた場合
(5) 上記(1)～(4)以外で、治験責任医師または治験分担医師が有害事象とすべきと判断した場合

表 7.1　有害事象の Grade 分類（重症度）基準

Grade	重症度
1	軽度。症状がない、または軽度の症状がある。臨床所見または検査所見のみ。治療を要さない。
2	中等度。最小限／局所的／非侵襲的治療を要する。年齢相応の身の回り以外の日常生活動作の制限*。
3	重症または医学的に重要であるが、ただちに生命を脅かすものではない。入院または入院期間の延長を要する。活動不能／動作不能。身の回りの日常生活動作の制限**。
4	生命を脅かす。緊急の処置を要する。
5	有害事象（AE）による死亡。

*　「身の周り以外の日常生活動作」とは食事の準備、日用品や衣類の買い物、電話の使用、金銭の管理等をさす。

**　「身の周りの日常生活動作」とは、入浴、着衣・脱衣、食事の摂取、トイレの使用、薬の服薬が可能で、寝たきりではない状態をさす。

7.2　有害事象

7.2.1　有害事象の定義

有害事象とは、本被験機器による治療を実施した被験者に生じたすべての好ましくない、または意図しない疾病または障害ならびにその徴候（臨床検査値の異常を含む）をいい、本被験機器との因果関係の有無は問わない。有害事象の収集は、本被験機器による治療以降、個々の被験者の観察期間終了または中止時までの期間（追跡調査期間は除く）とし、重症度は「National Cancer Institute Common Terminology Criteria for Adverse Events（NCI CTCAE v5.0）」に従って判定する。また、該当する項目がない場合は、表 7.1 を参考に Grade を判定する。

7.2.2　有害事象の記載

有害事象名は、原則として診断名・疾患名（病名）で症例報告書に記載する。診断名・疾患名が特定できない場合や治験責任医師または治験分担医師より診断名・疾患名としないことが妥当と判断された場合には、臨床症状または徴候（臨床検査値異常を含む）を有害事象名として症例報告書に記載する。

治験責任医師または治験分担医師は、発現したすべての有害事象に関し、有害事象名、発現日、転帰日、重症度、重篤性、重篤と判断した理由（7.2.3 重篤な有害事象(1)～(7)参照）、本被験機器との因果関係、バイオチューブとの因果関係、転帰（回復、軽快、後遺症あり、未回復、死亡）、被験機器に対してとられた処置、バイオチューブに対してとられた処置、有害事象の治療のためにとられた処置、コメントを症例報告書の有害事象欄に記載する。

7.2.3　重篤な有害事象

有害事象が下記のいずれかに該当する場合、重篤な有害事象として取り扱う。

(1) 死亡した場合
(2) 死亡につながる恐れのある場合
(3) 永続的または顕著な障害・機能不全に陥った場合
(4) 障害につながる恐れのある場合
(5) 治療のため入院または入院期間の延長が必要とされる場合
(6) 先天異常をきたすもの
(7) その他医学的に重篤な状態

なお、治験参加前（同意取得前）より予定していた療法または検査を治験実施中に実施することのみを目的とした入院（予定手術や検査等）、有害事象に伴う治療・検査の目的以外の入院（健康診断等）、好ましくない医療上のできごとが生じていない場合は重篤な有害事象として取り扱わない。

7.2.4　有害事象の重篤性の判定

有害事象の重篤性は、以下に分類される。

(1) 重篤
(2) 非重篤

7.2.5　有害事象の治療のためにとられた処置

有害事象を治療するために被験者に対してとられた処置は以下に分類される。

(1) あり（記載内容：薬物療法、その他（詳細））
　　※ありの場合は、症例報告書の併用薬・併用療法記載箇所に詳細情報を記載する。
(2) なし

7.2.6　被験機器に対してとられた処置

有害事象が発現した場合に、本被験機器に対してとられた処置は以下に分類される。

(1) なし

被験機器の使用を中止しなかった場合

(2) 使用中止

被験機器の使用を中止（被験機器を摘出）した場合

7.2.7　有害事象の転帰

有害事象の転帰は以下に分類される。

(1) 回復：有害事象が消失、または元の状態まで戻っている

(2) 軽快：有害事象は完全に回復していないものの、ほぼ消失、またはほぼ元の状態に戻っている

(3) 後遺症あり：有害事象は元の状態まで回復したものの、後遺症が残っている

(4) 未回復：有害事象は継続中である

(5) 死亡：有害事象の結果、死亡した

個々の被験者の観察期間終了日の時点で未回復の有害事象については、可能な限り回復または軽快となるまで追跡調査を行うこととするが、不可逆的な事象であった場合等、治験責任医師または治験分担医師が追跡不要と判断した場合は、被験者の観察期間終了日を以て追跡終了とすることができる。その場合は、症例報告書のコメント欄に追跡不要と判断した理由を記載する。

また、有害事象は下記の表 7.2.7 の判定基準に従って本被験機器との因果関係を 3 段階で判定し、症例報告書に記録する。表 7.2.7 に基づき、本被験機器との関連性で「関連性は否定できない」、「関連あり」のいずれかに該当したものを本被験機器との因果関係が否定できない事象とみなす。なお、「関連なし」に該当する場合は、判断した理由を症例報告書のコメント欄に記載する。

表 7.2.7　被験機器との因果関係判定基準

関連なし	被験機器との関連が明らかに否定できる場合
関連性は否定できない	被験機器との関連が疑われる場合
関連あり	被験機器との関連が明らかに認められる場合

7.3 不具合

7.3.1　不具合

不具合とは、本被験機器の仕様上の問題、本被験機器の不良・故障、または手技の問題等をいう。不具合については治験実施期間中に発生した情報を随時収集する。なお、本治験の特性に鑑み、被験機器摘出後もバイオチューブの不具合について調査を行う。

7.3.2　不具合情報の記録

治験責任医師または治験分担医師は本被験機器/バイオチューブに不具合が発生した場合、発生した不具合情報の内容を症例報告書へ記載する。不具合の安全度は表 7.3.2 に基づき判定する。

表 7.3.2　不具合の安全度

安全度 1	被験者及び術者等に影響を与える
安全度 2	被験者及び術者等に影響を与える可能性がある
安全度 3	被験者及び術者等に影響を与えない

7.4　有害事象及び不具合発生時の対応

(1) 治験責任医師または治験分担医師は、有害事象に対する治療が必要になったことを知った場合には、被験者にその旨を伝える。

(2) 治験責任医師または治験分担医師は、有害事象の発現に際しては適切な処置を施し、被験者の安全確保に留意し、その原因究明に努める。

(3) 治験責任医師または治験分担医師は、発現した症状あるいは臨床検査値の異常変動について、原則として当該事象が消失または治験開始前の状態に回復するまで、または臨床上問題とならないと判断されるまで、可能な限り経過観察を継続し、その転帰を確認する。

7.5　重篤な有害事象及び重篤な有害事象につながる恐れがある不具合が発生した場合の報告

治験中の被験者において有害事象が発生し、治験責任医師または治験分担医師が当該有害事象を重篤と判断した場合、以下の手順に従い当該有害事象を取り扱う。

(1) 治験責任医師から治験実施医療機関の長及び治験調整医師への報告

治験責任医師は、因果関係に関わらず当該有害事象情報を可能な限り速やかに実施医療機関の長に報告するとともに、治験調整医師に報告する。

(2) 治験調整医師による各実施医療機関の治験責任医師及び治験機器提供者への通知

治験調整医師は、治験責任医師から入手した当該有害事象報告の内容を確認し、他の各実施医療機関の治験責任医師及び治験機器提供者に当該有害事象情報を通知する。

(3) 治験責任医師と治験調整医師との協議

各実施医療機関の治験責任医師は、治験調整医師から入手した報告内容を確認し、必要に応じ治験責任医師と協議し、治験責任医師としての意見（厚生労働大臣への報告の必要性を含む）を治験調整医師に報告する。

(4) 効果安全性評価委員会の開催

治験調整医師が、当該有害事象が医薬品医療機器等法施行規則第 273 条に規定される報告対象であると判断した場合は、効果安全性評価委員会を開催し、治験の継続の可否について判断を求める。効果安全性評価委員会が治験の継続の可否について判断した場合は、治験調整医師に通知し、治験調整医師により治験責任医師へ通知する。

(5) 厚生労働大臣への報告

治験責任医師が「医薬品・医療機器等の品質、有効性及び安全性の確保等に関する法律」及び施行規則に定められた厚生労働大臣へ報告すべき不具合等に該当すると判断した場合には、治験調整医師は指定の様式で報告書を作成し、PMDA に報告する。

(6) 実施医療機関の長への報告

治験責任医師は、他の実施医療機関において発生した重篤な有害事象について厚生労働大臣への報告がなされた場合には、治験調整医師より入手した(5)の報告書の内容を、可能な限り速やかに実施医療機関の長に報告する。

(7) 追加情報の入手時の対応

当該有害事象が発生した実施医療機関の治験責任医師は、当該有害事象に関する追加情報が得られた場合には、可能な限り速やかに実施医療機関の長に追加報告を行うとともに、治験調整医師及び治験機器提供者に報告する。当該追加情報の取扱いは、上記(1)～(6)の手順に準ずることとし、必要に応じ PMDA への報告等を行う。

7.6　新たな情報の提供

治験責任医師は、被験者の安全性に悪影響を及ぼし、本治験の実施に影響を与える、または治験継続に関する IRB 等の承認を変更する可能性のある情報を得た場合には、速やかに実施医療機関の長に文書で報告し、説明文書を改訂する必要があると認めた時は速やかに改訂する。治験責任医師または治験分担医師は、被験者にその旨を通知し、適切な治療及び事後処理を保証する。

8. 有効性評価

8.1　主要評価項目

被験機器によるバイオチューブの形成能

中芯からバイオチューブを抜き取り直線化棒に挿入した時点で、炎症等の異常の有無を確認し形成状態を肉眼的に評価する。アルコールに浸漬させた後、直線化棒に挿入した状態でバイオチューブの総長を計測する。分断して形成されている場合は、断片毎と合計の長さを計測する。また、組織の厚さや均一性を肉眼的に調べる。直線化棒を抜去し、シリンジを用いて内腔を生理食塩水で加圧し、破損や漏れの有無を調べる。一部を採取して、引張試験機を用いて破断強度を計測する。後日、病理組織観察を行い、厚さ、炎症、コラーゲンの構造を調べる。

なお、本評価項目は、形成されたバイオチューブの品質を多面的に評価することを目的としているが、各症例においては、バイオチューブのみをバイパスグラフトとしてバイパス術を実施できた場合（バイオチューブの断片をつなぎ合わせて使用した場合を含む）について、本評価項目を「達成」したものとする。

8.2　副次評価項目

以下の 1)～8)の評価項目について副次的に評価する。

1) 被験機器の埋植・摘出に関する手技的成功

埋植時には、皮下ポケットが十分な大きさで作製でき、被験機器を破損や分解させることなく、皮下損傷なく挿入でき、皮下出血を制御して閉創できることを肉眼的に評価する。埋植期間中、血腫の形成や滲出液の貯留等の異常がないことを皮膚から肉眼的に評価する。摘出時には、被験機器と周囲組織との癒着の程度を確認し、出血を制御しながら、被験機器の破損や分解なく摘出できることを肉眼的に評価する。

2) 被験機器によって形成されたバイオチューブを用いた末梢側吻合を含むバイパス術の手技的成功

通常の静脈を用いたバイパス術と同様の手技にて、バイオチューブに破損なく、カッティングなく縫合可能であることを肉眼的に評価する。吻合部位からの出血が制御でき、バイオチューブ壁からの血液の漏れや破れ、膨化等がないことを肉眼的に評価する。血管クランプ解除後に拍動を伴う血流を確認し、流量を血流計で調べ、末梢血行再建を評価する。

なお、本評価項目における「手技的成功」とは、バイオチューブのみをバイパスグラフトとしてバイパス術を実施できた場合（バイオチューブの断片をつなぎ合わせて使用した場合を含む）をいい、自家静脈や人工血管とつなぎ合わせてバイパス術を実施した場合を含めない。

3) 移植後のバイオチューブの 12 週時点の開存

後観察期間中に下肢動脈エコー検査によってバイオチューブ内の血流を評価する。可能であれば下肢 CT 血管造影検査または下肢動脈造影検査によりバイオチューブの形状を観察し、狭窄や膨化等の異常の有無を評価する。

4) 症状の改善（虚血性疼痛の改善、創傷の改善）

適格性確認時、バイオチューブ移植時、バイオチューブ移植後 1 週・4 週・8 週・12 週時に疼痛の改善率、創傷改善率、創面積縮小率、肉芽形成率を評価する。なお、適格性確認時、バイオチューブ移植後 1 週・4 週・8 週・12 週時については WIfI スコアの評価も行う。

5) 大切断の回避

後観察期間中における標的下肢の大切断の回避率を調べる。

6) 被験機器埋植中及びバイオチューブ移植後の追加治療の実施率

被験機器埋植後から後観察期間が終了するまでの間に、バイオチューブを用いたバイパス術以外の血行再建術が実施された割合を評価する。

7) 被験機器埋植中及びバイオチューブ移植後の QOL

被験機器埋植から約 6 週間経過した時点を目安に実施したアンケートの結果を用いて、被験機器埋植部位の違和感等の有無、日常生活への影響の有無について評価する。また、後観察期間終了時に実施したアンケートの結果を用いて、バイオチューブ移植後の QOL について評価する。

8) バイパス術後のバイパスグラフト径

後観察期間中に、下肢動脈エコー検査によってバイパス術に使用したバイオチューブ（人工血管または自家静脈を使用した場合はそれらも含む）の径を経時的に調査し、評価する。

9. 統計解析

解析の主要な方針を以下の項目に示すが、より技術的で詳細な解析事項及び解析方法については、統計解析計画書に記載する。データ固定前に統計解析計画書を作成し、解析はデータ固定後に実施する。

個別の症例及びデータの取扱いについては、データ固定前の症例検討会において決定する。

9.1　解析対象集団

(1) 有効性解析対象

1) FAS（Full Analysis Set）

登録が行われた被験者のうち、以下の被験者を除いた被験者からなる解析対象集団を FAS とする。

- 本治験の対象となる重症下肢虚血患者ではなかった被験者
- 被験機器を一度も使用していない被験者
- 登録後の有効性データが全くない被験者

2) PPS（Per protocol set）

FAS から、以下の被験者を除いた被験者からなる解析対象集団を PPS とする。

- 選択基準に逸脱した被験者
- 除外基準に抵触した被験者
- その他、治験実施計画書で定める有効性評価に影響し得る規定に抵触した被験者

なお、中止症例の取扱いについても症例検討会において検討する。

(2) 安全性解析対象

登録が行われた被験者のうち、以下の被験者を除いた被験者からなる解析対象集団を安全性解析対象集団（Safety Analysis Set; SAS）とする。

- 被験機器を一度も使用していない被験者
- 登録後の安全性データが全くない被験者

9.2　有効性の解析

被験機器によるバイオチューブ形成能を主要評価項目とする。本治験は探索的治験であることから、被験機器の埋植・摘出に関連する手技や条件、被験機器により形成されたバイオチューブの性能を含め、多角的かつ総合的に評価する。

9.3　安全性の解析

安全性の評価のため、以下について評価し、有害事象や不具合の発生率等について解析する。

(1) 被験機器の皮下埋植期間中の被験機器との関連を否定できない炎症、腫瘍形成、死亡の発生

(2) 移植したバイオチューブの生体適合性（炎症、腫瘍形成等）

(3) 移植したバイオチューブの破裂

(4) 移植したバイオチューブとの関連を否定できない死亡の発生

(5) バイオチューブの移植後12週までの死亡の発生

(6) その他、有害事象及び不具合の発生

9.4　中間解析

バイオチューブ移植後12週が経過した症例が6例となった時点で、中間解析を実施する。

中間解析では、主要評価項目である被験機器によるバイオチューブ形成能及び副次評価項目のうち移植後のバイオチューブの12週時点の開存について評価を行う。

9.5　症例数の設定

目標登録症例数として、以下の設定根拠に基づき12例（対照群なし）と設定した。

＜設定根拠＞

被験機器は大小 2 種類があり、埋込場所は胸部、腹部、臀部または大腿部の 4 ヶ所から選択される。埋植部位と個数は、治験責任医師または治験分担医師の判断により、被験者に最も適していると思われる条件にて決定されるため、それぞれの埋植部位あたりの個数を均等に配分することはできないが、同一の部位、大きさ等の条件をそれぞれ複数の症例で検討するためには、最低 12 例が必要であると考える。本治験は探索的な位置付けの試験であり、実施可能性及び中間解析についても考慮し、登録症例数の目標を 12 例と設定した。

10. 治験の品質管理及び品質保証

本治験の品質を保証するために、治験責任医師及び実施医療機関は、標準業務手順書に従い治験の品質管理を行う。

10.1　本治験における品質マネジメント

治験において、品質管理及び品質保証は、治験の品質を確保するに当たって重要な役割を担っている。その品質管理及び品質保証をより効果的に活用して治験の品質を担保するため、本治験では、令和元年 7 月 5 日付薬生薬審発 0705 第 5 号厚生労働省医薬・生活衛生局医薬品審査管理課長通知「治験における品質マネジメントに関する基本的考え方について」に基づいて適切な体制を構築し、治験の品質についてのマネジメントを実施する。

10.2　原資料等の直接閲覧

治験責任医師及び実施医療機関の長は、モニタリング、監査ならびに IRB 及び国内外の規制当局による調査を受け入れ、原資料等のすべての治験関連記録を直接閲覧（複写含む）に供しなければならない。詳細は標準業務手順書に従う。なお、被験者は同意文書に署名することにより、直接閲覧を認めたことになる。

10.3　モニタリング

治験調整医師が指名するモニタリング担当者は、別に定める「モニタリングに関する手順書」に従い、本治験実施計画書に記載されているすべての内容ならびに「医療機器の臨床試験の実施の基準に関する省令（医療機器 GCP)」(平成 17 年厚生省令第 36 号 : 医療機器 GCP 省令）に従って治験が実施されているかを確認するため実施医療機関へのモニタリングを定期的に行う。

10.4　監査

治験調整医師が指名する監査担当者は、別に定める「監査の実施に関する手順書」に従い、実施医療機関及び開発業務受託機関において監査を実施する。

10.5　モニタリング及び監査への協力

治験責任医師及び実施医療機関の長ならびに開発業務受託機関の責任者は、モニタリング及び監査に協力しなければならない。

11. 倫理及び GCP 遵守

11.1　治験の倫理的実施

本治験は、ヘルシンキ宣言の精神に基づいて実施する。また、医薬品医療機器等法第 14 条第 3 項及び第 80 条の 2 に規定される基準、医療機器 GCP 省令等の関連法令通知及び本治験実施計画書を遵守して実施する。

11.2　被験者への説明と同意の取得

(1) 治験責任医師は、被験者から本治験への参加の同意を得るために用いる説明文書を作成し、必要な場合にはこれを改訂する。
(2) 作成または改訂された当該文書は、あらかじめ IRB の承認を得る。
(3) 被験者の権利を放棄させるかその懸念のある記載、あるいは治験責任医師または治験分担医師、実施医療機関、自ら治験を実施する者の法的責任を回避するかその懸念のある記載をしてはならない。
(4) 説明文書には、以下の事項を含むものとする。
    1) 本治験が研究を伴うこと。
    2) 本治験の目的。
    3) 治験責任医師の氏名及び連絡先。
    4) 本治験の方法。
    5) 予期される臨床上の利益及び危険性または不便。
    6) 対象患者に対する他の治療方法の有無、及び当該治療方法に関して予測される重要な利益及び危険性。
    7) 被験者の本治験への参加予定期間。
    8) 本治験への参加は被験者の自由意思によるものであり、被験者は、被験者の本治験への参加を随時拒否または撤回することができること。また拒否・撤回によって被験者が不利な扱いを受けることや、本治験に参加しない場合に受けるべき利益を失うことはないこと。

9) モニター、監査担当者、IRB 等及び規制当局が医療に係わる原資料を閲覧できること。その際、被験者の秘密は保全されること。同意文書に被験者が署名することによって閲覧を認めたことになること。
10) 本治験の結果が公表される場合であっても、被験者の秘密は保全されること。
11) 被験者が本治験及び被験者の権利に関してさらに情報の入手を希望する場合、または本治験に関連する健康被害が生じた場合に照会すべきまたは連絡を取るべき実施医療機関の相談窓口。
12) 本治験に関連する健康被害が発生した場合に被験者が受けることのできる補償及び治療。
13) 本治験に参加する予定の被験者数。
14) 本治験への参加の継続について被験者の意思に影響を与える可能性のある情報が得られた場合には、速やかに被験者に伝えられること。
15) 本治験への参加を中止させる場合の条件または理由。
16) 被験者が費用を負担する必要がある場合にはその内容。
17) 被験者に金銭等が支払われる場合にはその内容（支払額算定の取り決め等)。
18) 被験者が守るべき事項。
19) 本治験の適否等について調査審議を行う IRB の種類、IRB において調査審議を行う事項その他本治験に係る IRB に関する事項。
20) IRB の手順書、委員名簿、委員会の議事要旨等の情報が公開され閲覧できる旨。
21) その他、被験者の人権保護に関し必要な事項。

11.3 同意取得の方法

(1) 治験責任医師または治験分担医師は被験者に対し、本治験の参加に先立ち、IRB の承認を得た説明文書を使用して十分説明した後、自由意思による本治験の参加の同意を本人から文書で得る。
(2) 説明にあたっては、治験責任医師または治験分担医師は被験者から同意を得る前に、被験者が質問する機会と、本治験に参加するか否かを判断するのに必要な時間を与えなければならない。さらにその際、治験責任医師、治験分担医師または補足説明者としての治験協力者は、すべての質問に対して被験者が満足するように答えなければならない。
(3) 同意文書には、説明を行った治験責任医師または治験分担医師及び被験者が署名し、各自日付を記入する。なお、治験協力者が補足的に説明を行った場合には、当該治験協力者も署名し、日付を記入する。
(4) 治験責任医師または治験分担医師は、署名と日付が記入された同意文書の写し及び説明文書を、原則として被験者が本治験に参加する前に被験者に渡す。
(5) 同意文書の原本は実施医療機関で保存する。

11.4 説明文書の改訂

(1) 被験者または代諾者の同意の意思に関連し得る新たな重要な情報（通常、説明文書の改訂を必要とする情報）が得られた場合には、治験責任医師は速やかに当該情報に基づき説明文書を改訂し、IRB の承認を得る。

(2) 治験責任医師または治験分担医師は、改訂された説明文書を用いて改めて説明し、治験への参加の継続について被験者本人から自由意思による同意を文書により得る。
(3) 治験責任医師または治験分担医師は、新たに署名と日付を記入した同意文書の写し及び説明文書を被験者に渡す。同意文書の原本は実施医療機関で保存する。

11.5 治験審査委員会

本治験は、実施に先立ち、本治験を行うことの適否について倫理的、科学的及び医学的妥当性の観点から IRB の承認を得る。

11.6 被験者の人権保護に関する事項

治験責任医師または治験分担医師は、被験者の選定にあたって、人権保護の観点から選択基準及び除外基準に基づき、被験者の健康状態、症状、年齢、同意能力、治験責任医師または治験分担医師との依存関係、他の治験への参加の有無等を考慮の上、治験に参加を求めることの適否について慎重に検討する。

被験者の登録、検体の外部提出及び症例報告書作成における被験者の特定は被験者識別コードで行うとともに、治験実施医療機関においては、個人識別情報と被験者識別コードは対応表を作成し、対応表は各実施医療機関にて適切に管理する。治験の実施に係る原資料及び被験者の同意文書等の直接閲覧、ならびに治験成績の公表においては、被験者の氏名、疾患等のプライバシー保護に十分配慮する。

12. 症例報告書

12.1 記載方法

(1) 症例報告書の記載内容及び訂正に関しては治験責任医師または治験分担医師が責任を負う。各被験者の各観察・検査が終了後、2 週間以内を目安に症例報告書に記載する。症例報告書の記載や訂正の方法に関しては、症例報告書の記載の手引き等に従う。
(2) 治験協力者は、原資料が存在しその客観性が保証できる場合は、原資料から症例報告書に転記することができる。
(3) 治験責任医師は、作成された症例報告書についてその内容を点検し、確認した上で署名する。
(4) 治験責任医師は、作成した症例報告書を定められた手順にて提出する。
(5) 治験責任医師は、症例報告書と原資料に矛盾がある場合、その理由を説明する記録を作成の上定められた手順にて提出する。
(6) 症例報告書に記載されたデータのうち、「12.3 症例報告書中の記載内容を原資料とすべき項目の特定」にある項目については、治験責任医師または治験分担医師が症例報告書へ直接記載する場合は、原資料の有無は不問とする。

12.2 原資料

原資料とは、治験の結果として症例報告書に記載されるデータの基になる資料をいう。

(1) 被験者の同意及び情報提供に関する記録
(2) 診療録（検査伝票や画像検査データ等を含む)、看護記録等の症例報告書作成の基となった記録
(3) 被験機器の使用に関する記録

12.3　症例報告書中の記載内容を原資料とすべき項目の特定

症例報告書に記載されたデータのうち、以下に示す項目については、症例報告書の記載内容を以て原資料とする。ただし、診療録やワークシート等の一次資料が存在する場合は、当該一次資料を原資料とする。

・合併症の有無
・併用薬の使用理由
・有効性評価、有効性に関するコメント
・安全性に関するコメント
・併用薬・併用療法の有無
・中止の有無、理由、コメント
・有害事象の有無、重篤性、重症度、被験機器に対してとられた処置、有害事象の治療のためにとられた処置、バイオチューブに対してとられた処置、転帰日、転帰、被験機器との因果関係の有無、バイオチューブとの因果関係及びコメント
・不具合の有無、種類、安全度、不具合に伴う有害事象の有無、被験機器に対してとられた措置、バイオチューブに対してとられた処置、被験機器との因果関係の有無、バイオチューブとの因果関係、転帰日、転帰及びコメント

13. 治験の費用負担及び補償

13.1　利益相反について

本治験は、AMED の研究費で実施される。

本治験で使用する被験機器 BTM1 は、バイオチューブ株式会社より、共同研究契約に基づき無償提供される。本治験における利益相反については、世界医師会ヘルシンキ宣言において、被験者への資金提供、スポンサー、利益相反に関する十分な説明と研究計画書への記載が求められていることを踏まえ、治験実施計画書に記載するものとする。該当事項として、実施医療機関である横浜総合病院の治験責任医師は、バイオチューブ株式会社の役員であり株式を所有しているが、本治験の実施にあたり大分大学医学部臨床研究利益相反マネジメント委員会による審査を受け、適切に利益相反マネジメントを実施する。また、各実施医療機関においても、利益相反のマネジメントについては、適切に実施する。なお、利益相反マネジメント方法については、各実施医療機関の基準に委ねる。

13.2　健康被害補償及び保険

本治験の実施に起因して被験者に健康被害が発生した場合には、実施医療機関は治療その他必要な措置を講ずるものとし、実施医療機関あるいは被験者の故意もしくは重過失により生じた場合を除き、治験責任医師が適切な補償を行う。また、本治験に起因する健康被害であって、賠償責任が生じた場合には、その原因を生じせしめた当事者（治験責任医師、実施医療機関等）の按分に沿って賠償金額を分担する。治験調整医師及び治験責任医師は、賠償責任及び補償責任の履行措置として、保険その他の必要な措置をとるものとする。

13.3　予定される治験費用及び被験者への支払い

治験期間中の医療費は、被験機器及び治験責任医師が当該実施医療機関で負担すると取り決めた処置・検査費用等を除き、被験者が負担する。

14. 治験実施計画書の逸脱または変更及び改訂

本治験の実施に際しては、治験責任医師は、治験実施計画書を実施医療機関の長を経由して IRB に提出し、あらかじめ IRB の承認を経て実施医療機関の長の承認を得なければならない。本治験は、治験責任医師と実施医療機関の長の合意のもとに本治験実施計画書を遵守して実施する。

14.1　治験実施計画書の逸脱または変更

(1) 治験責任医師または治験分担医師は、IRB の事前の審査に基づく文書による承認を得ることなく、治験実施計画書からの逸脱または変更を行わない。

(2) 治験責任医師または治験分担医師は、被験者の緊急の危険を回避する等、医療上やむを得ない場合、IRB の事前の審査に基づく文書による承認を得ることなく、治験実施計画書からの逸脱または変更を行うことができる。その際には、治験責任医師は、逸脱または変更の内容及び理由を記載した文書を、直ちに実施医療機関の長に提出し、IRB の承認を得る。

14.2　治験実施計画書の改訂

(1) 治験責任医師は、本治験の進行中に治験実施計画書に改訂の必要性が生じた場合は、その内容について、必要に応じて治験調整医師と協議する。

(2) 治験責任医師は、治験実施計画書、説明文書を改訂した場合は、治験実施計画書改訂版、説明文書改訂版を速やかに実施医療機関の長に提出する。

(3) 治験責任医師は、IRB の承認を経て実施医療機関の長の承認を得た後に、当該治験実施計画書改訂版ならびに当該説明文書改訂版を遵守して本治験を行わなければならない。

15. 治験の中止・中断・終了

本治験の一部及び全体の中止・中断規定は以下のとおりとし、いずれの場合も中止時点の成績の収集及び解析を行う。なお、個々の被験者の中止基準については、「4.4 中止基準」に記載した。

15.1　治験の中止・中断

本被験機器の開発の妥当性が否定された場合及び予期しない重篤な有害事象が認められ本治験の実施が医学的・倫理的に実施不可能と判断された場合等、本治験の進行中に治験全体を中止または中断せざるを得ない理由が生じた場合、治験責任医師は治験調整医師と協議し、治験の中止・中断及びその理由の詳細を実施医療機関の長及び規制当局、治験調整医師（治験調整事務局）に速やかに通知する。実施医療機関の長は IRB にその旨を通知し、文書で詳細を説明する。治験責任医師または治験分担医師は、被験者にその旨を通知し、適切な治療及び事後処理を保証する。

15.2　治験の終了

本治験が終了した場合には、治験責任医師は実施医療機関の長にその旨を文書で通知し、治験結果の概要を文書（治験終了報告書）で報告する。実施医療機関の長は、IRB に対し速やかに終了の旨を文書で通知するとともに、治験終了報告書に基づき、治験結果の概要を報告する。

16. 効果安全性評価委員会について

効果安全性評価委員会は、治験の継続の適否または治験実施計画書の変更について審議することを目的として、治験調整医師によって設置され、「効果安全性評価委員会に関する手順書」に従い、治験の進行、安全性データ及び重要な有効性評価項目を評価するものである。

開催時期及び目的の詳細は「3.4 症例登録の制限」のとおりとする。

17. 記録等の保存

(1) 実施医療機関の長は、実施医療機関において保存すべき治験に係る文書または原資料を、次の 1)または 2)のうちいずれか遅い日まで保存する。ただし、治験責任医師または実施医療機関の長がこれよりも長期間の保存を必要とする場合には、保存期間及び保存方法について治験責任医師または実施医療機関の長と協議する。
   1) 本被験機器に係る製造販売承認日（開発が中止された場合には開発中止が決定された日から 3 年が経過した日）
   2) 本治験の中止または終了後 3 年が経過した日

(2) IRB の設置者は、業務手順書、委員名簿（各委員の資格を含む）、委員の職業及び所属のリスト、提出された文書、会議の記録、その概要及び書簡等の記録を次の 1)または 2)のうちいずれか遅い日まで保存する。ただし、治験責任医師がこれよりも長期間の保存を必要とする場合には、保存期間及び保存方法について治験責任医師と協議する。
   1) 本被験機器に係る製造販売承認日（開発が中止された場合には開発中止の連絡を受けた日から 3 年が経過した日）
   2) 本治験の中止または終了後 3 年が経過した日

(3) 自ら治験を実施する者は、保存すべき治験に係る文書または記録を、次の 1)または 2)のうちいずれか遅い日までの間保存する。
   1) 本被験機器に係る製造販売承認日（開発が中止された場合には開発中止が決定された日から 3 年が経過した日）
   2) 本治験の中止または終了後 3 年が経過した日

18. 公表に関する取決め

治験責任医師または治験分担医師は、本治験により得られた情報を学会等外部に公表しようとする場合には、あらかじめ文書により治験調整医師及び被験機器提供者の承諾を得るものとする。

19. 治験実施体制

別紙参照

20. 参考文献

1. Norgren L, Hiart WR, Dormandy JA, Nehier MR, Harris KA, Fowkes FG. Inter-Society Consensus for the Manegement of peripheral artery disease (TASC II). J Vasc Surg 2007; 45(Suppl): S5-67
2. Dua A, Lee CJ. Epidemiology of peripheral artery disease and critical limb ischemia. Tech Vasc scInteventional Rad 2016; 19: 91-5
3. Alamasri J, Adusumalli J, Asi N, Lakis S, Alsawas M, Prokop LJ et al. A systematic review and meta-analysis of revascularization outcomes of infrainguinal chronic limb-threatening ischemia. J Vasc Surg 2018; 68: 624-33
4. Bradbury AW, Adam DI, Bell J, Forbes JF, Fowkes FGR, Gillespie I, et.al. Bypass versus angioplasty in severe ischemia of the leg (BASIL) trial: an intention-to treat analysis of amputation-free and overall survival in patients randomized to a bypass-first or a balloon angioplasty-first revascularization strategy. J Vasc Surg 2010; 51: 5S-17S
5. Bradbury AW, Adam DI, Bell J, Forbes JF, Fowkes FGR, Gillespie I, et. al. Bypass versus angioplasty in severe ischemia of the leg (BASIL) trial: analysis of amputation free and overall survival by treatment received. J Vasc Surg 2010; 51: 18S-31S.
6. Iida O, Nakayama M, Yamauchi Y, Kawasaki D, Yokoi Y, Yokoi H et al. Endovascular treatment for infrainguinal vessels in patients with critical limb ischemia. OLIVE registry, a prospective, multicenter study in Japan with 12-month follow-up. Circ Cardiovasc Interv 2013; 6: 68-76
7. Iida O, Takahara M, Soga Y, et al; Three-year outcomes of surgical versus endovascular revascularization for critical limb ischemia: The SPINACH Study (Surgical Reconstruction Versus Peripheral Intervention in Patients with Critical Limb Ischemia). Circ Cardiovasc Interv 2017; 10:
8. Conte MS, Bradbury AW, Kolh P, White JV, Dick F, Fitridge R, et.al. Global vascular guidelines on the management of chronic limb-threatening ischemia. Eur J Vasc Endovasc Surg 2019; 58: S1-S109.
9. Furukoshi M, Moriwaki T, Nakayama Y. Development of an in vivo tissue-engineered vascular graft with designed wall thickness (Biotube type C) based on a novel caged mold. J artif Organs 2016; 19:54-61
10. Ishi D, Enmi J, Moriwaki T, Ishibashi-Ueda H, Kobayashi M, Iwana S et al. Development of in vivo tissue-engineered microvascular grafts with an ultra small diameter of 0.6 mm (MicroBiotubes): acute phase evaluation by optical coherence tomography and magnetic resonance angiography. J artif Organs 2016; 19: 262-9
11. Ishi D, Enmi J, Iwai R, Kurisu K, Tatsumi E, Nakayama Y. One year rat study of iBTA-induced "Microbiotube" maicrovascula grafts with an ultra-small diameter of 0.6 mm. Eur J Vasc Endovasc Surg 2018; 55: 882-7
12. Nakayama Y, Furukoshi M. Terazawa T, Iwai R. Development of long in vivo tissue-engineered "Biotube" vascular graft. Biomaterals 2018; 185: 232-9.
13. Nakayama Y, Higashita R, Shiraishi Y, Umeno T, Tajikawa T, Yamada A et. al. iBTA-induced Biotube blood vessels: 2020 update. Kidney Dial 2021; 1: 3-13.

14. Higashita R, Nakayama Y, Shiraishi Y, Iwai R, Inoue Y, Yamada A, et al. Acute phase pilot evaluation of small diameter long iBTA induced vascular graft "Biotube" in a goat model. EJVES Vasc Forum. 2022; 54: 27–35.
15. Mills Sr JL Conte MS, Armstrong DG, Pomposelli FB, Schanzer A, Sidawy AN et al. The Society for Vascular Surgery Lower Extremity Thretened Limb Classification System: risk stratification based on wound, ischemia, and foot infection (WIfI). J Vasc Surg 2014; 59: 220-34.
16. Schanzer A, Hevelone N, Owens CD, Belkin M, Bandyk DF, Clowes AW et al. Technical factors affecting autogenous vein graft failure: Observations from a large multicenter trial. J Vasc Surg 2007; 46: 1180-90.
17. Slim H, Tiwari A, Ritter JC, Rashid H. Outcome of infra-inguinal bypass grafts using conduit with less than 3 millimeters diameter in critical leg ischemia. J Vasc Surg 2011; 53: 421-5.
18. Hata Y, Iida O, Takahara M, Asai M, Masuda M, Okamoto S et al. Saphenous vein size as a surrogate marker for mortality of patients with chronic limb-threatening ischemia undergoing endovascular therapy. J Cardiol 2021; 78: 341-6.
19. Yamamoto S, Deguchi J, Hashimoto T, Suhara M, Sato O. Relationship between the controlling nutritional status score and infrainguinal bypass surgery outcomes in patients with chronic limb-threatening ischemia. Ann Vasc Dis 2021; 14: 334-6
