# Supplementary material for: Safety and efficacy of an iBTA-induced autologous Biotube vascular graft and its preparation device BTM1 in below-the-knee bypass surgery for chronic limb threatening ischemia: A protocol for an open-label, single-arm, multicenter clinical trial
Source: PLoS One. 2025 Nov 6;20(11):e0335900. doi: 10.1371/journal.pone.0335900 (PMC12591420; doi:10.1371/journal.pone.0335900)
Supplement: S3 File — (PDF) [file pone.0335900.s003.pdf]

Safety and efficacy of an iBTA-induced autologous Biotube® vascular graft and its preparation device BTM1 in below-the-knee bypass surgery for chronic limb threatening ischemia: A protocol for an open-label, single-arm, multicenter clinical trial

"Investigator-initiated clinical trial"

Clinical trial implementation plan

Clinical trial coordinating physician

Oita University Hospital

Department of Cardiovascular Surgery

Shinji Miyamoto

Clinical trial implementation plan number: B-80

Version number: 2.0

Date of creation: September 5, 2022

Table of contents

1. Development history
   - 1.1 Background of the Clinical Trial Plan
   - 1.2 Position and validity of this clinical trial
   - 1.3 Summary of non-clinical trials
   - 1.4 Test device
   - 1.5 Summary of known and potential risks and benefits to subjects
     - 1.5.1 Expected risks of using this test device
     - 1.5.2 Expected benefits of using this test device
2. Purpose of the clinical trial
3. Clinical trial design
   - 3.1 Type and design of clinical trials
     - 3.1.1 Types of clinical trials
     - 3.1.2 Clinical trial designs
   - 3.2 Evaluation items
   - 3.3 Subject participation period
   - 3.4 Clinical trial restrictions
   - 3.5 Planned period of clinical trial
4. Subject selection/exclusion, discontinuation criteria
   - 4.1 Target disease
   - 4.2 Inclusion criteria
   - 4.3 Exclusion criteria
   - 4.4 Discontinuation criteria
5. Provisions for concomitant medications, concomitant therapies, and tests
   - 5.1 Concomitant medications
   - 5.2 Concomitant therapies
   - 5.3 Testing restrictions
6. Clinical trial procedures
   - 6.1 Information acquisition
   - 6.2 Eligibility confirmation
   - 6.3 Subject enrollment
   - 6.4 Use of test device
     - 6.4.1 Clinical trial procedures
     - 6.4.2 Procedures for using the investigational device BTM1
     - 6.4.3 Matters related to biotube transplantation (bypass surgery)
     - 6.4.4 Duration of implantation of the test device and follow-up period

6.5 Observation and examination schedule
6.5.1 Observation/examination/survey contents
6.5.2 Examination and observation after discontinuation
7. Safety evaluation
7.1 Clinical test values
7.2 Adverse events
7.2.1 Definition of adverse events
7.2.2 Description of adverse events
7.2.3 Serious adverse events
7.2.4 Assessment of the severity of adverse events
7.2.5 Actions taken to treat the adverse event
7.2.6 Actions taken with the investigational device
7.2.7 Outcome of the adverse event
7.3 Malfunction
7.3.1 Malfunction
7.3.2 Recording of malfunction information
7.4 Responses to adverse events and malfunctions
7.5 Reporting of serious adverse events and malfunctions that may lead to serious adverse events
7.6 Providing new information
8. Efficacy evaluation
8.1 Primary endpoint
8.2 Secondary endpoints
9. Statistical analysis
9.1 Analysis population
9.2 Efficacy analysis
9.3 Safety analysis
9.4 Interim analysis
9.5 Setting the number of cases
10. Quality control and assurance of clinical trials
10.1 Quality management for this clinical trial
10.2 Direct viewing of source documents, etc.
10.3 Monitoring
10.4 Audit
10.5 Cooperation with monitoring and audits
11. Ethics and compliance with GCP

11.1 Ethical conduct of clinical trials
11.2 Explanation to subjects and acquisition of informed consent
11.3 Method of obtaining consent
11.4 Revision of information document
11.5 Clinical trial review committee
11.6 Matters related to protecting the human rights of subjects
12. Case report form
12.1 How to write
12.2 Source material
12.3 Identification of items in case report form that should be source documents
13. Cost sharing and compensation for clinical trials
13.1 Conflict of interest
13.2 Health damage compensation and insurance
13.3 Planned clinical trial costs and payments to subjects
14. Deviations, changes, and revisions to the clinical trial protocol
14.1 Deviations or changes to the clinical trial protocol
14.2 Revisions to the clinical trial protocol
15. Cancellation, suspension, or termination of a clinical trial
15.1 Stopping or suspending a clinical trial
15.2 Ending a clinical trial
16. About the Efficacy and Safety Evaluation Committee
17. Storage of records, etc.
18. Disclosure agreements
19. Clinical trial implementation structure
20. References

List of abbreviations, abbreviations and terms

ABI	Ankle Brachial Index
AE	Adverse Event
ALT（GPT）	Alanine Aminotransferase<br>（Glutamic Pyruvic Transaminase）
APTT	Activated Partial Thromboplastin Time
AST（GOT）	Aspartate Aminotransferase<br>（Glutamic Oxaloacetic Transaminase）
BUN	Blood Urea Nitrogen
CLTI	Chronic Limb Threatening Ischemia
CONUT	Controlling Nutritional Status
CRF	Case Report Form
CRP	C-Reactive Protein
CT	Computed Tomography Scan
GLASS	Global Limb Anatomic Staging System
GVG	Global Vascular Guideline
IRB	Institutional Review Board
LDH	Lactate Dehydrogenase
LDL	Low-density Lipoprotein
PT	Prothrombin Time
PT-INR	International Normalized Ratio of Prothrombin Time
QOL	Quality of Life
SPP	Skin Perfusion Pressure
WIfI	Wound, Ischemia, and foot Infection

Clinical trial protocol overview

Clinical trial name	Safety and efficacy of an iBTA-induced autologous Biotube® vascular graft and its reparation device BTM1 in below-the-knee bypass surgery for chronic limb threatening ischemia: A protocol for an open-label, single-arm, multicenter clinical trial (Investigator-initiated clinical trial)
Purpose	This clinical trial will target patients with critical limb ischemia who do not have an appropriate autologous vein available for bypass surgery, and will aim to evaluate the safety and efficacy of bypass surgery to the leg artery below the knee or foot artery using a biotube created subcutaneously with the test device "BTM1."
Test Equipment	BTM1
Clinical trial design	Multicenter, open-label, uncontrolled single-arm, exploratory clinical trial
Target Patients	Patients with severe lower limb ischemia who require bypass surgery with peripheral anastomosis to the calf artery or foot artery below the knee, but who do not have an appropriate autologous vein.
Inclusion criteria	1. Agreement for participating in the study and informed consent signed by the patient.<br>2. Age is over 18 years old.<br>3. Lower limb ischemia that meets grade 3 or 4 (ABI* < 0.6, SPP < 40 mmHg) in Wifi classification of CLTI.<br>4. Patients correspond to one of the following.<br>(a) GLASS classification of GVG is stage III, and corresponds to clinical stage 2, 3 or 4 of Wifi classification.<br>(b) GLASS classification is stage II, and corresponds to clinical stage 3 or 4 of Wifi classification.<br>(c) GLASS classification is stage I or II, and clinical symptoms do not improve even if intravascular treatment is performed.<br>5. Upper or lower limb veins of the optimal length and diameter required for bypass surgery does not exist.<br>6. Patients with arterial occlusion on the proximal side of the planned proximal anastomosis site for bypass surgery.<br>7. Survival of 12 months or more and followed-up for 12 weeks after bypass surgery are possible.

Exclusion criteria	1. Patients who have difficulty in securing the implantation period of the clinical trial equipment required for Biotube formation.<br>2. Patients with general condition who are difficult to tolerate surgery due to severe malnutrition and complications.<br>3. Patients with poor skin condition who cannot secure more than one implantation site for the investigational device.<br>4. Patients undergoing invasive surgery within 30 days prior to enrollment<br>5. Patients who do not have a peripheral target artery that can be bypassed or who have undergone endovascular treatment at the planned anastomosis site on the peripheral side.<br>6. Patients with arterial occlusion on the proximal side of the planned proximal anastomosis site for bypass surgery.<br>7. Patients who cannot confirm blood flow on the distal side of the planned peripheral anastomosis site for bypass surgery.<br>8. Patients with lower limb amputation proximal to the metatarsals.<br>9. History and complications of malignant tumors (excludes those with no recurrence for more than 5 years after treatment or new onset).<br>10. Patients who use immunosuppressants for autoimmune diseases and post-implantation.<br>11. Patients who have a history of allergies to stainless steel or polyolefin resin.<br>12. Pregnancy.<br>13. Participating in other clinical trials.<br>14. Patients judged by the investigator to be inappropriate due to medical conditions or safety reasons.
Discontinuation criteria	The clinical trial will be discontinued for subjects who meet the following criteria:<br>1. When the principal investigator or co-investigator determines that the clinical trial will be difficult to continue due to the occurrence of adverse events or progression of the underlying disease.<br>2. If no biotube usable for bypass surgery, even in fragments, is formed, making it impossible to continue the clinical trial thereafter

	3. If the subject withdraws consent to participate in the clinical trial<br>4. If a subject is found to not meet eligibility requirements after enrollment<br>5. If the subject becomes pregnant after implantation of the study device and before implantation of the biotube<br>6. If a significant deviation from the clinical trial protocol is discovered<br>7. If bypass surgery is not required 24 weeks after BTM1 implantation, the entire BTM1 will be removed and the clinical trial will be discontinued, but the formation ability of the biotube will be evaluated.<br>8. If the principal investigator or co-investigator determines that the clinical trial should be discontinued
Tests and observations	See separate table (observation and inspection schedule)
Efficacy endpoints	[Primary evaluation items]<br>Ability of forming biotubes using the test device<br>[Secondary evaluation item]<br>1) Procedural success of implanting and removing the test device<br>2) Procedural success of bypass surgery including distal anastomosis using biotubes formed by the test device<br>3) Patency of the biotube after implantation at 12 weeks<br>4) Improvement of symptoms (improvement of ischemic pain, improvement of wounds)<br>5) Avoidance of major amputation<br>6) Rate of additional treatment during implantation of the test device and after implantation of the biotube<br>7) QOL during implantation of the study device and after implantation of the biotube<br>8) Bypass graft diameter after bypass surgery
Safety evaluation items	1) Inflammation, tumor formation, and death that cannot be ruled out as being related to the test device during the period of subcutaneous implantation of the test device<br>2) Biocompatibility of the implanted biotube (inflammation, tumor formation, etc.)<br>3) Rupture of implanted biotubes

	4) Deaths that cannot be ruled out as being related to implanted biotubes<br>5) Deaths within 12 weeks after implantation of the biotube<br>6) Other adverse events and malfunctions
Target number of cases	12 cases
Duration of clinical trial	August 2022 to May 2026 (Registration period: August 2022 to November 2025)

[Table] Observation and examination schedule

	Pre-observation period			Test device implantation period					Post-observation period							Discontinuation	
	Visit (V) 1			V2	V3	V4	V5	V6	V7	V8	V9	V10	V11	V12	V13	Test device implantation period	Post-observation period
	Obtaining consent	Confirming eligibility	Subject enrollment	Implantation of the test device	Removal of the break drain	1 week after drain removal	6 weeks after implantation of the test device	Removal of the test device	Biotube transplant	1 day after transplant	3 days after transplant	1 week after transplant	4 weeks after transplant	8 weeks after transplant	12 weeks after transplant		
Obtaining consent	●																
Subject background		●															
Subject registration			●														
Height and weight		●															
Subjective and objective findings		●		●			●	●	●	●	●	●	●	●	●	●	●
Vital signs		●		●			●	●	●	●	●	●	●	●	●	●	●
Blood test		●		●			●	●	●	●	●	●	●	●	●	●	●
Lower limb arterial ultrasound		●					●										
Upper limb and lower limb venous ultrasound		●															
Lower limb CT angiography		▲										▲			△		
Lower limb arterial angiography		▲										▲			△		
Lower limb X-ray		●										●	●	●	●	●	●
ABI test		●										●	●	●	●	●	●
SPP test		●										●	●	●	●	●	●
Ischemia status evaluation		●										●	●	●	●	●	●
Lower limb MRI test		○															
Third-party qualification verification		●															
Implantation of test device				●													
Echo test of test device implantation site					●	●											
Break drain removal					●												
Questionnaire survey							●								●	●	●
Extraction of test device								●								●	
Biotube quality evaluation								●	●								
Biotube sample storage								●	○							●	
Biotube transplantation									●								
Concomitant medications/combined therapies																●	●
Adverse events																●	●
Malfunctions																●	

●: mandatory ○:optional or possible ▲: both or either △: both or either if possible

1. Development history

1.1 Background of the clinical trial plan

Severe limb ischemia presents with ischemic rest pain, foot ulcers, gangrene, etc., and in Japan, approximately 10,000 patients undergo amputation annually. The prognosis after amputation is extremely poor, with a 1-year mortality rate of 25% and a contralateral amputation rate of 25%, making it a life-threatening condition (References 1, 2). If revascularization is not possible, the patient will have to amputate the lower limb. Revascularization of the lower limbs is generally performed by endovascular therapy, which is a catheter treatment, or bypass surgery using autologous veins or artificial blood vessels (Reference 3). Endovascular treatment of the popliteal artery below the knee to the calf artery often results in restenosis or occlusion, and bypass using thin artificial blood vessels also has poor results (References 4-6), and bypass using autologous veins has the best results (References 7, 8). However, if autologous veins cannot be used due to reasons such as varicose veins, thin veins, or the veins having already been used for bypassing the coronary arteries or peripheral arteries, limb salvage becomes difficult.

There is a long and thin artificial blood vessel that can be used for below-knee bypass. Although artificial blood vessels made of synthetic materials such as ePTFE (expanded polytetrafluoroethylene) and PET (polyethylene terephthalate) have shown satisfactory results in terms of patency and durability for large diameters (10 mm or more), they are insufficient for medium diameters (6-8 mm), and there are no satisfactory products for small diameters (5 mm or less) used for lower limb peripheral bypass or coronary artery bypass. A practical artificial blood vessel made from biomaterials or using conventional tissue engineering has not yet been completed. By using in-Body Tissue Architecture (iBTA) developed by Nakayama, a thin and long autologous tubular tissue (biotube) that can be used for below-knee bypass can be created by subcutaneously implanting a mold for 1-2 months (References 9-13). The BioTube enables surgical revascularization for patients with severe lower limb ischemia, who were previously unable to undergo revascularization, and is expected to improve the pathology of insufficient circulation below the knee and achieve long-term patency through autologous revascularization. This test device was designated as a medical device under the Sakigake Designation System of the Ministry of Health, Labor and Welfare in fiscal year 2019, and is classified as a new medical device of class III. With support from the Medical Engineering Collaboration Project of the Japan Agency for Medical Research and Development (AMED) in fiscal year 2019, the product specifications for the mold were determined and non-clinical trial methods were prepared.

1.2 Positioning and validity of this clinical trial

This clinical trial is an exploratory investigator-initiated clinical trial with the primary objective of evaluating the biotube formation ability of the test device BTM1 based on in vivo tissue formation therapy.

In this study, non-clinical trials (efficacy, performance, and safety tests) were conducted under the AMED Translational Research Strategic Promotion Program in FY2020, based on the conceptual requirements advised in the medical device pre-development consultation with the Pharmaceuticals and Medical Devices Agency (PMDA) held in November 2019. In addition, in an implantation test using goats, the biotube formation ability of BTM1 was confirmed. Furthermore, a transplantation test of the biotube in goats was conducted (Reference 14), and it was confirmed that the biotube was replaced with vascular tissue in about 3 months and remained patency for 12 months.

Prior to evaluating the efficacy and safety of this treatment in a confirmatory clinical trial, this clinical trial will first verify the biotube formation ability of BTM1 in humans.

1.3 Summary of non-clinical studies

The test device does not actually perform any treatment or diagnosis. The biotube made using the test device is used as an implant for treatment. Therefore, the following requirements were placed on the test device and the biotube. The test device must 1) be capable of being implanted subcutaneously, 2) maintain the shape (including the gap between the core and the outer shell) and 3) strength required for biotube formation even after implantation, 4) form a biotube that can be used for surgical bypass surgery after a certain period of implantation, 5) be able to remove the device and 6) the biotube without damage, and 7) be safe as a medical device. The biotube must meet the following requirements: 8) have a shape and quality (including thickness and uniformity) required clinically, 9) have mechanical strength and flexibility, 10) be implantable, 11) after implantation, autologous cells infiltrate the biotube as a scaffold to form tissue similar to autologous blood vessels, 12) not be decomposed or absorbed for the clinically required period, 13) long-term patency is achieved without anastomotic failure at the site of use, and 14) be connectable, and 15) be preserved. Test items considered necessary to verify safety, efficacy, and stability were selected and performed for each requirement of the test device and the biotube. Tests considered necessary were also performed on the sizer, straightening rod, and soaking dish, which are related accessories. All tests were met, and it was confirmed that the test device has appropriate performance, safety, and efficacy for use in humans.

1.4 Test Equipment

1.4.1 Name and configuration of the test device

The name of this test device is "BTM1", and it consists of a "core", "outer shell" (2 types of symmetrical shapes), an "inner frame" and an "outer frame". There are two types, 40 cm and 55 cm, depending on the length of the biotube to be produced. In addition, it includes a "sizer", a "straightening rod" and an "immersion dish" as accessories.

[Test device: Photo of BTM1 (left: for 55 cm, right: for 40 cm)]

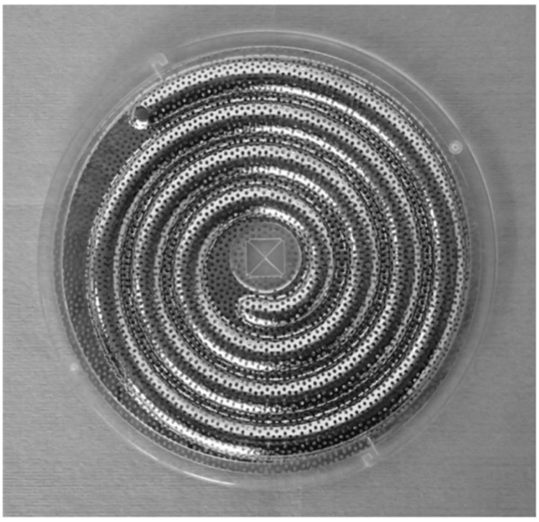

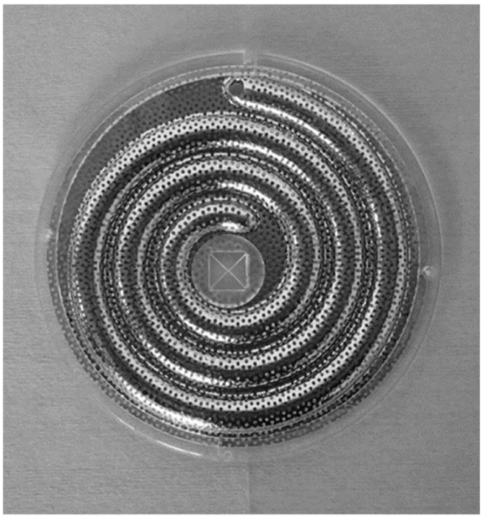

[Test device: Structure and size of BTM1 (top: for 55 cm, bottom: for 40 cm)]

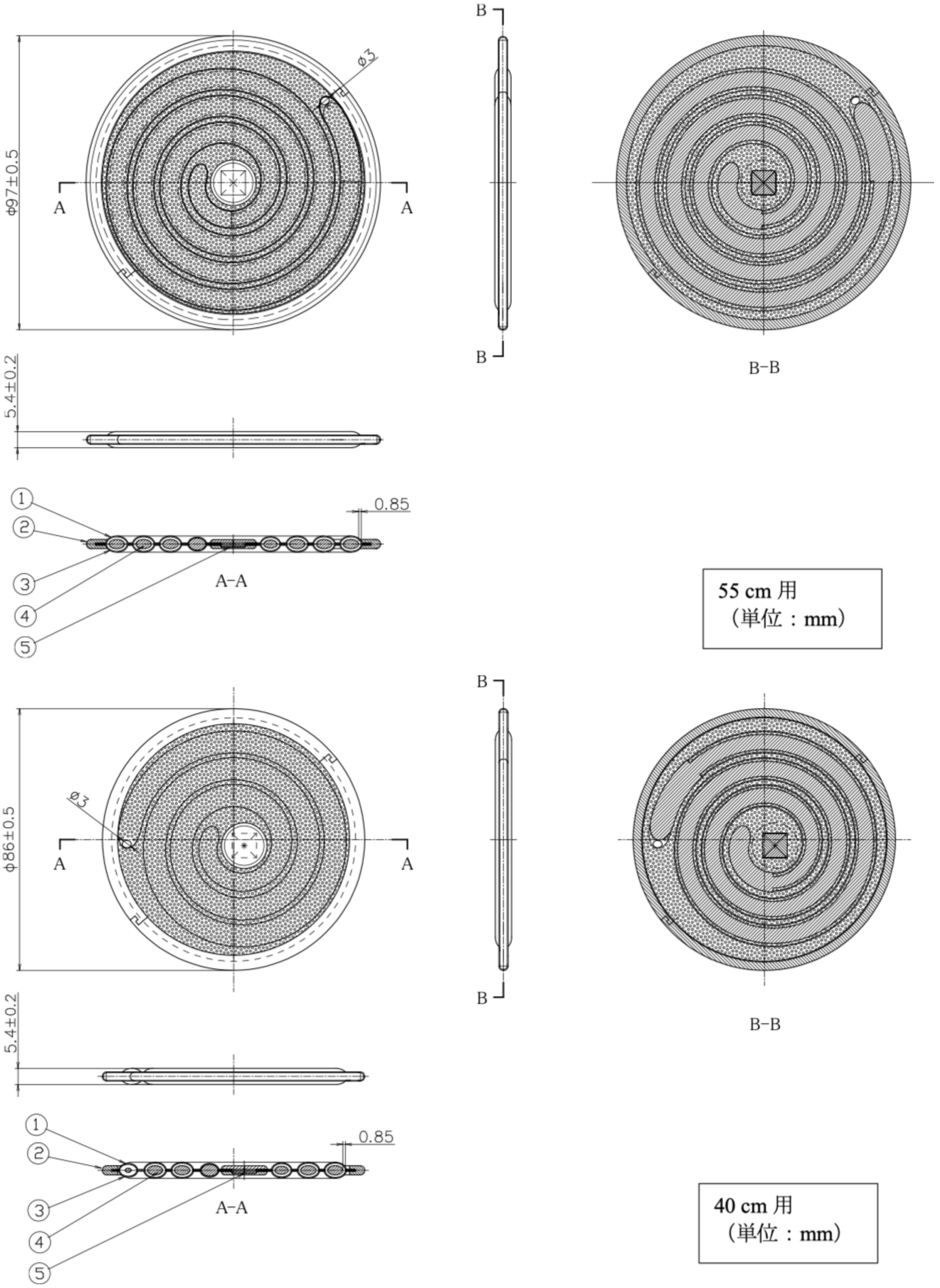

① Outer shell A ② Outer frame ③ Core ④ Outer shell B ⑤ Inner frame

[Accessories: Sizer photo (left: for 55 cm, right: for 40 cm)]

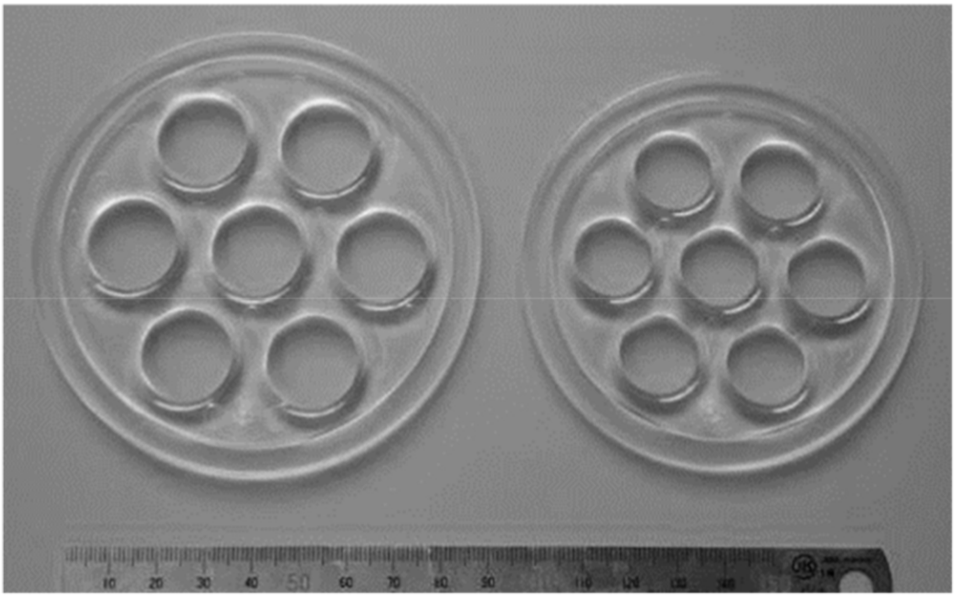

[Accessories: Sizer structure and size (left: for 55 cm, right: for 40 cm)]

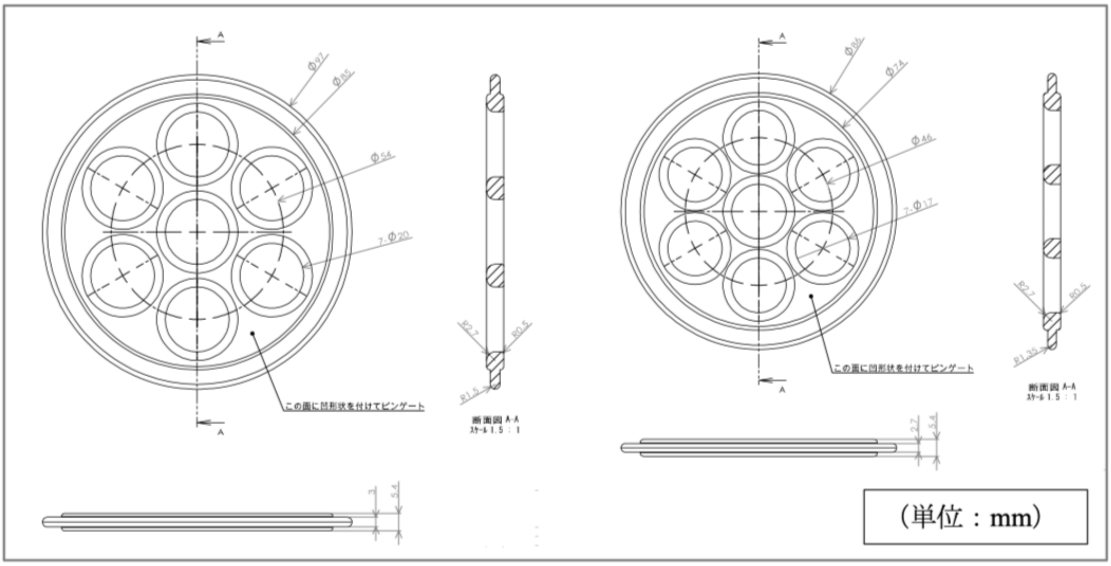

[Accessories: Photo of straightening rod]

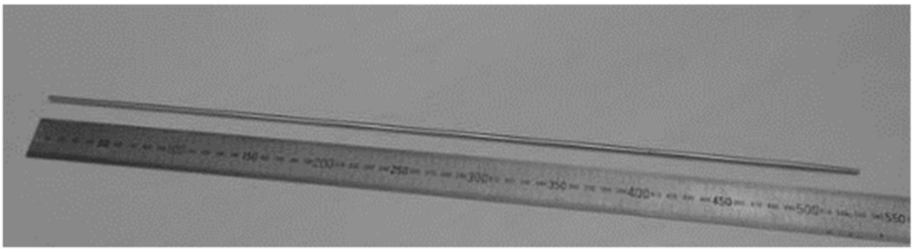

[Accessories: Straightening rod structure and size]

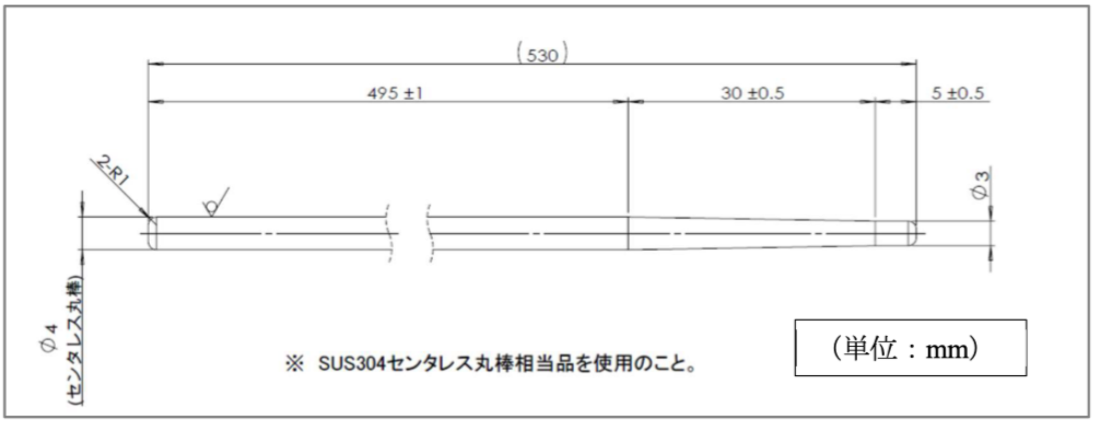

[Accessories: Photo of soaking dish]

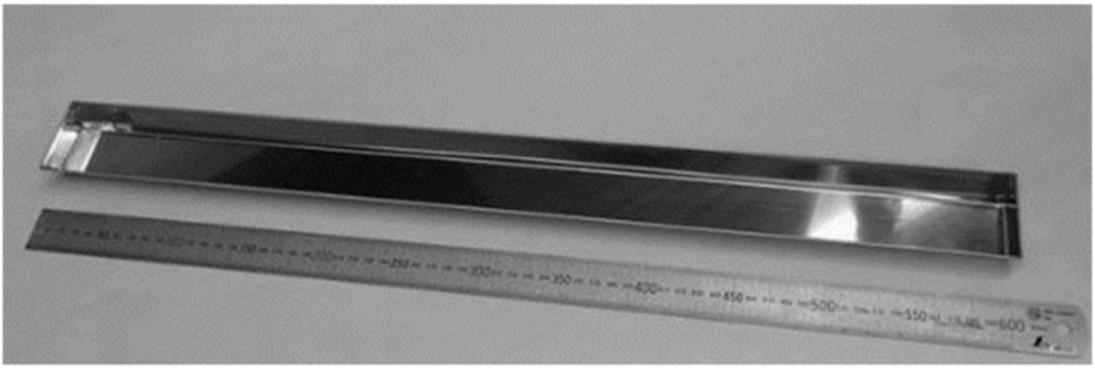

[Accessories: Structure and size of immersion dish]

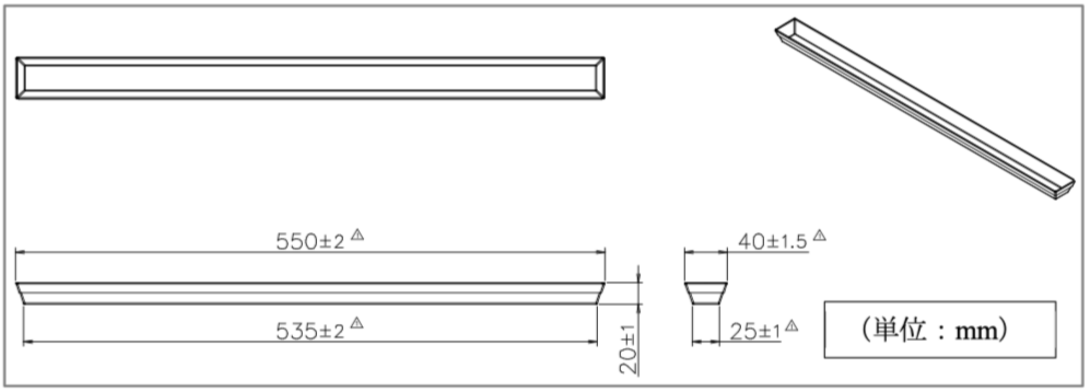

1.4.2 display

(1) Packaging form

Each box contains one sealed BTM1 in a sterile bag.

(2) Exterior labeling

The exterior packaging of the investigational device should indicate that it is for clinical trial use, the clinical trial protocol number, clinical trial identification code, size of the investigational device, manufacturing number, storage method, expiration date, and the name, affiliation, job title, and address of the clinical trial coordinating physician, as follows:

治験用　　治験実施計画書番号：B-80

BTM1

（●● cm）

治験識別記号：BTM1
製造番号　　：XXXXXXX
保管方法　　：室温保存
使用期限　　：20XX年XX月XX日

治験調整医師
氏名　　　　：宮本　伸二
所属・職名：大分大学医学部附属病院　心臓血管外科・医師
住所　　　　：大分県由布市挾間町医大ヶ丘1丁目1番地

1.4.3 Storage and management procedures

The investigational device will be stored at room temperature at each medical institution, avoiding high temperatures and humidity. An investigational device manager will be appointed to ensure the proper storage and management of the investigational device. The investigational device manager will store the investigational device appropriately in accordance with the "Procedures for Management of Investigational Devices," and will create an investigational device management list to keep track of the usage of the investigational device and the progress of the clinical trial.

1.5 A summary of known and potential risks and benefits to subjects

1.5.1 Anticipated risks associated with use of this test device

The implantation of BTM1 and the use of the biotube produced by BTM1 may cause the following malfunctions and adverse events in subjects. Malfunctions that do not directly affect the safety of subjects will be described in the Investigational Device Brochure.

(1) Malfunctions and Adverse Events (BTM1)

・ Deformation, damage, disintegration, etc. of BTM1 during subcutaneous implantation

・ Infection or allergic/anaphylactic reactions due to BTM1 during subcutaneous implantation and associated symptoms (shock, seizures, fever, nausea/vomiting, convulsions, etc.)

・ Injury due to physical damage to BTM1 during implantation, implantation, or removal (bleeding, skin injury, subcutaneous injury, perforation, pain and tenderness (head, neck), fractures, etc.)

(2) Malfunctions and adverse events (biotube)

・ Major formation (insufficient length, strength, etc.):

If the length of the fabricated biotube is insufficient for bypass surgery alone due to poor formation, revascularization may be attempted by joining together multiple pieces of the biotube, or by anastomosis with an artificial blood vessel or autologous vein, etc. Also, if no usable pieces are formed at all, bypass surgery itself may not be possible.

・ Deformation, breakage, rupture, damage, etc. during or after transplantation

・ Poor revascularization due to thrombus formation, stenosis, or blockage

(3) Other risks

If a sufficient implantation period cannot be secured or if the clinical trial needs to be terminated due to some reason, such as rapid progression of the primary disease, it may not be possible to perform bypass surgery even if BTM1 is implanted.

1.5.2 Anticipated benefits of using this test device

By using the biotube made with BTM1 for bypass surgery, it may be possible to treat patients who are difficult to treat with existing lower limb revascularization procedures. The possible benefits of performing bypass surgery using the biotube are shown below.

(1) Benefits of being able to revascularize using the biotube

Avoidance of major amputation, wound healing of ulcers, relief and elimination of pain, recovery of walking, improvement of activities of daily living, prevention of

decline in cognitive function, improvement of life prognosis (reduction in mortality rate), avoidance of contralateral limb amputation, reduction in medical costs

(2) Benefits of biotubes being autologous tissue

・ It is less likely to cause infection, and even if an infection does occur, it is easier to manage than artificial objects.

・ Even if it becomes blocked after implantation, it is naturally absorbed and does not need to be removed.

2. Clinical trial objectives

The purpose of this clinical trial is to evaluate the safety and effectiveness of bypass surgery to the leg artery below the knee or foot artery using a biotube created subcutaneously using the test device "BTM1" in patients with severe lower limb ischemia who do not have an optimal saphenous vein.

3. Clinical trial design

3.1 Types and designs of clinical trials

3.1.1 Types of clinical trials

Exploratory investigator-initiated clinical trials

3.1.2 Clinical trial design

Open-label, uncontrolled, multicenter clinical trials

[Rationale for setting]

This clinical trial was conducted as an open-label, uncontrolled study because there was no comparable medical device for patients with critical limb ischemia who do not have an optimal saphenous vein, and the purpose of the study was to exploratory investigate the efficacy and safety of using a biotube created subcutaneously using BTM1 in place of an autologous vein in bypass surgery to the leg artery or foot artery.

3.2 Evaluation items

(1) Efficacy evaluation items

1) Primary evaluation items

Ability of forming biotubes using the test device

2) Secondary evaluation item

① Procedural success of implantation and removal of the test device

② Procedural success of bypass surgery, including distal anastomosis, using a biotube formed by the test device

③ Patency of the biotube at 12 weeks after implantation

④ Improvement of symptoms (improvement of ischemic pain, improvement of wounds)

⑤ Avoidance of major amputation

⑥ Rate of additional treatment during implantation of the study device and after implantation of the biotube

⑦ QOL during implantation of the test device and after implantation of the biotube

⑧ Bypass graft diameter after bypass surgery

[Rationale for setting]

First, it is necessary to confirm that the biotube formed using the test device can be used in bypass surgery and function as a substitute blood vessel. Next, it is necessary to examine whether the treatment is effective and whether symptoms can be improved to avoid amputation. Since symptoms usually improve in 12 weeks with bypass surgery using veins, it is necessary to observe the patient for up to 12 weeks.

(2) Safety evaluation items

① The occurrence of inflammation, tumor formation, or death that cannot be ruled out as being related to the test device during the period of subcutaneous implantation of the test device

② Biocompatibility of the implanted biotube (inflammation, rejection, tumor formation, etc.)

③ Burst of implanted biotubes

④ Deaths related to implanted biotubes cannot be ruled out

⑤ Deaths within 12 weeks after implantation of biotubes

⑥ Other adverse events and malfunctions

[Rationale for setting]

Since the medical device is implanted in the body, it is necessary to carefully check whether it has any adverse effects on the body. In addition, the formed biotube is made of the patient's own tissue, but since the tissue formed subcutaneously will be used as blood vessels in another part of the body, it is

necessary to confirm that it can be safely implanted without any malfunctions and functions as a blood vessel.

3.3 Subject participation period in the clinical trial

The participation period for each subject in the clinical trial will be from the date of consent to the end of the post-observation period (12 weeks after biotube implantation), excluding follow-up periods associated with adverse events.

3.4 Restrictions on case registration

Considering that this is the first clinical trial using BTM1 in humans, and to ensure the safety of subjects, the following measures will be taken to limit case enrollment.

① One month after transplantation, a "case enrollment limit" will be set, at which point the Efficacy and Safety Evaluation Committee will decide whether to continue the clinical trial.

② The restriction on case enrollment cannot be lifted at any of the medical institutions participating in the trial until one successful case has been achieved.

③ Case registration at other participating medical institutions will not be possible until one successful case has been achieved by the third case at the first participating medical institution.

④ The clinical trial will be terminated if three consecutive cases are unsuccessful at each participating medical institution, or across all participating medical institutions.

⑤ An Efficacy and Safety Evaluation Committee will be held for all cases one month after transplantation.

⑥ If any problems arise, an Efficacy and Safety Evaluation Committee will be held each time.

3.5 Planned period of clinical trial

August 2022 to May 2026 (Registration period: August 2022 to November 2025)

4. Subject selection, exclusion, and discontinuation criteria

Patients who give written consent to participate in the clinical trial, who meet all of the "4.2 Inclusion Criteria" at the time of registration, and who do not violate any of the "4.3 Exclusion Criteria" will be considered as subjects of this clinical trial.

4.1 Target diseases

Patients with severe lower limb ischemia who require bypass surgery to connect to the calf artery or foot artery below the knee, but who do not have an appropriate autologous vein.

4.2 Selection Criteria

(1) Patients who have given written consent to participate in the clinical trial
(2) Patients who are 18 years of age or older at the time of consent
(3) Patients with severe lower limb ischemia who meet the comprehensive WIfI classification of chronic limb threatening ischemia (CLTI) severity criteria (reference 15) of ischemia grade 2 or 3 (ABI <0.6, SPP <40 mmHg)
(4) Patients who are recommended to undergo lower limb artery bypass surgery and who meet any of the following criteria:
a: Stage III limb ischemia according to the Global Anatomic Staging System (GLASS) classification of the Global Vascular Guideline (GVG) (Reference 8) and clinical stage 2, 3, or 4 according to the WIfI classification
b: GLASS stage II limb ischemia and clinical stage 3 or 4 of the WIfI classification
c: GLASS stage I or II limb ischemia in which sufficient blood flow cannot be achieved even with endovascular treatment (including clinical failure in which the wound does not heal and clinical symptoms do not improve)
(5) Patients who do not have an appropriate upper or lower limb vein (or lower limb vein in the case of dialysis patients) that meets the length and diameter (≥3 mm) required for bypass surgery (References 16 - 18) and has no abnormal findings such as aneurysm
(6) Patients who require peripheral anastomosis of a bypass to a calf artery below the knee or a foot artery
(7) Patients who are judged to be able to survive for ≥12 months and be able to be followed up for 12 weeks after bypass surgery at the time of obtaining informed consent

[Rationale for setting]

(1), (2), (7) To select appropriate patients from ethical and safety standpoints
(3) To select patients who require revascularization
(4) To select patients who are best suited to bypass surgery when selecting a treatment method
(5) To select patients who do not have a suitable autologous vein for a bypass graft
(6) Because existing artificial blood vessels cannot be used as a bypass graft in this area

4.3 Exclusion criteria

(1) Patients whose general condition makes it difficult to secure the implantation period of the test device required for biotube formation due to reasons such as the need for immediate revascularization.
(2) Patients whose general condition makes it difficult to tolerate surgery due to severe malnutrition (CONUT score 8-12) (Reference 19) or severe complications, etc.
(3) Patients who may not be able to secure two or more implantation sites for the test device due to reasons such as poor skin condition or a history of exposure to subcutaneous implants in the past.
(4) Patients who had undergone invasive surgery within 30 days prior to enrollment (excluding those undergoing treatment for leg wounds)
(5) Patients who do not have a peripheral target artery required for bypass surgery or who have a history of endovascular treatment at the site of the proposed peripheral anastomosis
(6) Patients who have arterial occlusion proximal to the planned central anastomosis site for bypass surgery
(7) Patients who cannot confirm blood circulation distal to the planned peripheral anastomosis site of bypass surgery (excluding those who can confirm collateral circulation)
(8) Patients who have had a lower limb amputation proximal to the midfoot
(9) Patients with a history of or comorbid malignant tumors (excluding those who have had no recurrence or new onset for more than 5 years after treatment)
(10) Patients who are using immunosuppressants due to comorbid autoimmune diseases or after transplantation, etc.
(11) Patients with a history of allergies to stainless steel or polyolefin resins
(12) Patients who are pregnant or may be pregnant
(13) Patients who are participating or planning to participate in other clinical trials or interventional clinical studies
(14) Patients who are judged by the investigator or co-investigator to be inappropriate for this clinical trial due to medical condition or safety reasons.

[Rationale for setting]
(1), (2), (4), (9)-(12) To ensure the safety of subjects
(3), (5)-(8) To properly evaluate the efficacy and safety of the test device
(13), (14) To conduct the clinical trial ethically and safely

4.4 Discontinuation criteria

(1) When the principal investigator or co-investigator determines that it is difficult to continue the clinical trial due to the occurrence of adverse events or aggravation of the underlying disease.
(2) When no biotube usable for bypass surgery, even fragmented ones, is formed at all, making it impossible to continue the clinical trial thereafter
(3) If a subject withdraws consent to participate in the clinical trial
(4) If it is discovered after enrollment that the subject does not meet the eligibility requirements
(5) If it is discovered that the subject is pregnant after implantation of the investigational device but before implantation of the biotube
(6) If a significant deviation from the clinical trial protocol is discovered
(7) If bypass surgery is not required 24 weeks after BTM1 implantation, the entire BTM1 will be removed and the clinical trial will be discontinued, but the formation ability of the biotube will be evaluated.
(8) Other cases in which the principal investigator or clinical investigator determines that the clinical trial should be discontinued

[Rationale for setting]

All of these were set from an ethical perspective and from the perspective of ensuring the. safety of the subjects.

5. Provisions regarding concomitant medications, concomitant therapies and testing

5.1 Concomitant medications

The following medications are prohibited from being used in combination for the specified period.

[Drugs that are prohibited from use throughout the clinical trial period after consent is obtained]

・ Drugs for chemotherapy of malignant tumors

・ Drugs with immunosuppressive effects (excluding those intended for systemic effects)

[Drugs prohibited for use before biotube transplantation]

The use of anticoagulants, except for heparin, is prohibited for the periods specified below.

・ Warfarin: From 3 days prior to transplant until the day before transplant

・ Direct-acting oral anticoagulants: The day before transplant

5.2 Combination therapy

After biotube implantation, appropriate antithrombotic therapy is performed to prevent the formation of blood clots. In principle, heparin is administered for 7 days after biotube implantation, then switched to oral anticoagulants, which are continued until 12 weeks after implantation. During heparin administration, APTT should be controlled to be within 1.5 to 2 times the preoperative level (Visit 7). Clopitogrel or cilostazol is administered alone as an antiplatelet drug, and dual antiplatelet drug combination therapy with aspirin is not performed. There are no particular regulations or restrictions on other combination therapies.

5.3 Inspection Restrictions

MRI examinations are not permitted during the BTM1 implantation period (Visit 2 to Visit 6). However, there are no restrictions on MRI examinations after BTM1 removal.

6. Clinical trial procedures

6.1 Obtaining consent

(1) When selecting subjects, the principal investigator or clinical trial co-investigator will carefully consider the appropriateness of including the patient in the clinical trial, taking into account the patient's health condition, symptoms, age, sex, ability to consent, and participation in other clinical trials, etc.

(2) The principal investigator or clinical investigator will fully explain the clinical trial to patients who are deemed appropriate as subjects of the clinical trial and obtain written consent from the patient (for details, see "11.2 Explanation to subjects and acquisition of consent" and "11.3 Method of obtaining consent").

6.2 Eligibility Verification

(1) The principal investigator or co-investigator will create a subject screening list for subjects who have given consent to participate in this clinical trial and assign a subject identification code.

(2) The investigator or clinical investigator will conduct the necessary tests and surveys from the time of consent until registration and determine eligibility to participate in this clinical trial based on the inclusion and exclusion criteria (see "7.5 Observation and Examination Schedule").

In addition to the judgment of the investigator or co-investigator, it is mandatory to hear the opinion of a third party with appropriate expertise (such as specialized staff in

vascular treatment or wound management) when confirming eligibility. The investigator will establish a system for this purpose within each medical institution.

6.3 Subject Enrollment

(1) The investigator or co-investigator shall verify the eligibility of subjects who have given consent as described in "6.1 Obtaining Consent" as described in "6.2 Eligibility Verification".

(2) The investigator or subinvestigator shall register subjects who have been confirmed as eligible for this clinical trial in accordance with a procedure to be determined separately. After confirming that registration has been completed, the investigator or subinvestigator shall implant the investigational device.

6.4 Use of the test device

6.4.1 Clinical trial procedures

The principal investigator or subinvestigator will administer the study treatment to the subjects enrolled in this study. The investigator will conduct the investigations and tests specified in "6.5 Observation and Examination Schedule" and "6.5.1 Observation, Examination, and Investigation Content" to confirm the safety of the investigational device and evaluate its effectiveness.

6.4.2 Instructions for using the test device BTM1

(1) How to make a biotube

1) The skin of the chest, abdomen, buttocks, or thigh will be incised and the subcutaneous pocket will be created by peeling off the dermis. The number and location of BTM1 implants for each subject will be

determined according to ① to ③ below.

① The number of implants will be a minimum of 2 and a maximum of 4.

② It is mandatory to implant one in the abdomen.

③ The remaining number of implants (1 to 3) excluding ② and their

implantation locations (chest, abdomen, buttocks, or thigh) will be determined based on the subject's physical build and preferences, as well as the medical judgment of the principal investigator or co-investigator (sufficient space for implantation, no skin abnormalities in the relevant area, etc.).

2) Insert the sizer into the created subcutaneous pocket and confirm that a space large enough for implantation has been secured.
3) Aseptically remove the BTM1 from the packaging bag and confirm that there is no deformation or abnormality on the outside. The size of the BTM1 to be used will be selected at the discretion of the principal investigator or clinical trial investigator depending on the subject's physique, etc.
4) Insert the BTM1 into the subcutaneous pocket and leave it subcutaneously.
5) If subcutaneous bleeding occurs, completely stop the bleeding with an electric scalpel, etc.
6) A break drain is inserted subcutaneously and secured in place.
7) Suture intradermally, secure BTM1 subcutaneously, and close the wound.
8) Commence suction of air from BTM1 through the break drain
9) Two to seven days after implantation, an ultrasound examination and visual inspection of the implantation site are performed to confirm that bleeding and exudate have subsided, and then the break drain is removed. The implantation site is also checked by ultrasound examination approximately one week after removal of the break drain. If an ultrasound examination reveals accumulation of blood or exudate around BTM1, it is removed by puncture.
10) Leave BTM1 in place for 4 to 24 weeks after implantation (generally at least 8 weeks).
11) The skin is incised again, and the subcutaneous tissue is dissected while controlling the bleeding to extract BTM1.
12) After removing the connective tissue around BTM1, the outer shell is removed and the formed biotube is extracted together with the core.
13) The biotube is removed from the core and washed with physiological saline.
14) A straightening rod is inserted into the lumen of the biotube and it is immersed in alcohol (70%) in an immersion dish for 30 minutes.
15) The biotube is washed again with saline, and visually inspected to ensure that there are no tears, defects, or significant deviations in wall thickness
16) Store in heparinized saline until use for transplantation.
17) As with bypass surgery using an autologous vein graft, load saline into the inside of the biotube with a syringe to confirm that there are no leaks,

and use a pressure monitor to check for pressure resistance (presence or absence of swelling or rupture under a pressure load of 200 mmHg). Also, use a tensile tester to measure the maximum strength and confirm that it meets the standard strength (5 N).

The biotube prepared using steps 1) to 17) above is transplanted into the subject's lower limb as a substitute blood vessel, in the same way as an autologous vein graft. If the investigational device cannot be removed and the biotube implanted on the same day due to the subject's general condition or other reasons, the prepared biotube may be temporarily stored. When temporarily storing, the biotube should be immersed in 10% alcohol while maintaining a sterile state, sealed, and stored at room temperature (if the prepared biotube is in fragments, it should be stored as is). In this case, the temporary storage period should be a maximum of 4 weeks, and the biotube implantation should be performed within 4 weeks of the investigational device removal. In addition, the investigator should make appropriate preparations before the start of the clinical trial, such as securing a storage location and creating storage procedures, to prevent loss or mix-up during temporary storage.

[Rationale for setting]

The results of non-clinical trials have confirmed that the shape and strength of the. BioTube did not change significantly for six months at room temperature. From the above, it has been confirmed that temporary storage is possible, but in order to prevent the condition from worsening, it is preferable to transplant the BioTube as soon as possible, and at the latest within four weeks after removal of the test device. Therefore, the temporary storage period was set at a maximum of four weeks (at room temperature).

(2) Strength test and histopathological evaluation of biotubes

1) Samples for strength test and pathological tissue evaluation are taken from the biotubes to be used for transplantation. The length of the sample to be taken is approximately 0.5-2 cm, but it is acceptable to adjust it appropriately according to the length of the biotube produced according to a procedure to be determined separately.
2) The strength test and histopathological evaluation are carried out using the samples stored in 1) according to a procedure to be separately determined (if

the biotube produced is in fragments, each fragment is tested). In principle, the strength test is carried out before the biotube is transplanted, as specified in 16) of (1).

[Rationale for setting]

To evaluate whether biotubes can be used for transplantation, it is necessary to examine their strength. It is also necessary to examine the tissue composition of the formed biotube and understand the differences depending on the site and subject. For these reasons, it was decided to conduct strength tests and histopathological evaluations.

(3) Timing of quality evaluation of biotubes

When the quality evaluation of the biotube is carried out according to the procedures (1) and (2), it shall be carried out at the times shown below. If the produced biotube is in fragments, in principle, the evaluation shall be carried out for each fragment.

1) When temporary storage of the biotube is not performed (BTM1 removal and biotube transplantation are performed on the same day)

Period		Items to be performed	Biotube condition		
			Can be used without connecting	Fragmented	
				Before connecting	After connecting
Visit 6	Immediately after BTM1 removal	Appearance	○	○	-
		Pressure resistance	○	○	-
		Strength	○	○	-
		Sample storage for pathological tissue evaluation	○	○	-
Visit 7	Before biotube transplantation	Appearance	-	-	-
		Pressure resistance	-	-	○
		Strength	-	-	-
		Sample storage for pathological tissue evaluation	-	-	-

2) When the biotube is temporarily stored (BTM1 removal and biotube transplantation are performed on different days)

Period of implementation		Items to be performed	Biotube condition		
			Can be used without connecting	Fragmented	
				Before connecting	After connecting
Visit 6	Immediately after BTM1 removal	Appearance	○	○	-
		Pressure resistance	○	○	-
		Strength	○	○	-
		Sample storage for pathological tissue evaluation	○	○	-
Temporary storage (only if necessary, up to 4 weeks)			○	○	-
Visit 7	Before biotube transplantation	Appearance	○	○	-
		Pressure resistance	○	○	○
		Strength	○	○	-
		Sample storage for pathological tissue evaluation	△	△	-

(4) Evaluation of biotubes that did not result in implantation

If the clinical trial is discontinued for any reason before the implantation of the biotube and the implanted test device is removed, the appearance, strength, and pathological tissue of the biotube at that time will be evaluated according to procedures (1) and (2).

[Rationale for setting]

The main purpose of this clinical trial is to evaluate the biotube formation ability, and even if the implantation is not achieved, if the test device is properly removed, it is possible to evaluate the biotube formation ability.

(5) Handling of remaining biotubes that are not used for transplantation

Biotubes that are surplus after transplantation or that are not used for transplantation for some reason will be stored in 10% alcohol with the assumption that they will be used for other research purposes.

(6) Discontinuation of test device implantation

1) If a local adverse event (infection, inflammation, exposure of the test device, etc.) occurs at the implantation site of the test device, implantation at that site will be discontinued and the device will be appropriately removed and treated. If no abnormalities are found at other implantation sites, implantation of the remaining test devices will continue.
2) If an adverse event accompanied by a deterioration of the patient's overall condition makes it difficult to continue implantation of the test device or to perform biotube transplantation according to the schedule set out in the clinical

trial protocol, in principle all test devices will be removed and the clinical trial will be discontinued.

6.4.3 Biotube transplant (bypass surgery)

(1) Performing biotube transplantation (bypass surgery)

1) Use of biotube alone

If the biotube formed by the test device meets the quality requirements for use in bypass surgery in terms of length, thickness, strength, etc., bypass surgery will be performed using only the biotube as a bypass graft. This also includes cases where the required length can be secured using only the biotube by connecting fragments of the biotube together.

2) Combination of biotube with autologous vein or artificial blood vessel

If the biotube formed by the test device does not meet the quality requirements for use in bypass surgery in terms of length, thickness, strength, etc. (including cases where the required length cannot be secured even by connecting fragmented biotubes together), bypass surgery will be performed using the biotube in combination with autologous vein or artificial blood vessel.

(2) Postponement or Cancellation of Biotube Transplantation (Bypass Surgery)

In the following cases, biotube transplantation (bypass surgery) will be canceled and the clinical trial will be discontinued. Even if the trial is discontinued, the biotube will be evaluated according to the procedure specified in "6.4.2 (4) Evaluation of Biotubes that were not Transplanted".

・ Cases in which no biotube of usable quality was formed.

・ Cases in which major amputation of the target limb was necessary due to wound infection causing the extent of necrosis to increase.

・ Cases in which the patient's general condition makes it difficult to perform biotube transplantation due to the occurrence of adverse events, etc. However, temporary postponement rather than cancellation is permitted only when the principal investigator or co-investigator determines that biotube transplantation can be considered within the allowable period specified in the clinical trial protocol.

6.4.4 Implantation period of the test device and follow-up period

Implantation period of the test device: 4 to 24 weeks (in principle, 8 weeks or more)
Post-observation period: up to 12 weeks after implantation of the biotube

In addition, this period includes the pre-observation period from consent acquisition to registration (up to 8 days) and the follow-up period for adverse events (if adverse events that require follow-up occur).

[Rationale for setting]

The longer the implantation period, the more the tissue formation of the biotube. progresses, but to avoid excessive worsening of the symptoms of lower limb ischemia, it is desirable to perform bypass surgery as soon as possible. Therefore, although it depends on the symptoms, the implantation period was set to about 2 months (8 weeks) in principle to ensure the formation of the biotube. In addition, since it was confirmed in non-clinical studies that the biotube could be formed after one month of implantation, it was considered acceptable to perform removal and bypass surgery even if the implantation period was between four and eight weeks if significant progression of ischemia was observed after implantation, and therefore the shortest implantation period was set at four weeks.

In addition, since symptoms usually improve around three months after implantation when bypass surgery is performed using an autologous vein, and since the results of non-clinical studies suggest that the implanted biotube is generally replaced by autologous tissue around three months after implantation, the observation period after implantation of the biotube was set at 12 weeks.

6.5 Observation and inspection schedule

Observations and inspections will be carried out according to Table 6.5 below.

Table 6.5 Observation and inspection schedule

	Pre-observation period			Test device implantation period					Post-observation period							Discontinuation	
	Visit (V) 1			V2	V3	V4	V5	V6	V7	V8	V9	V10	V11	V12	V13		
	Obtaining consent	Confirming eligibility	Subject enrollment	Implantation of the test device	Removal of the break drain	1 week after drain removal	6 weeks after implantation of the test device	Removal of the test device	Biotube transplant	1 day after transplant	3 days after transplant	1 week after transplant	4 weeks after transplant	8 weeks after transplant	12 weeks after transplant	Test device implantation period	Post-observation period
Obtaining consent	●																
Subject background		●															
Subject registration			●														
Height and weight		●															
Subjective and objective findings		●		●			●	●	●	●	●	●	●	●	●	●	●
Vital signs		●		●			●	●	●	●	●	●	●	●	●	●	●
Blood test		●		●			●	●	●	●	●	●	●	●	●	●	●
Lower limb arterial ultrasound		●					●										
Upper limb and lower limb venous ultrasound		●															
Lower limb CT angiography		▲										▲			△		
Lower limb arterial angiography		▲										▲			△		
Lower limb X-ray		●										●	●	●	●	●	●
ABI test		●										●	●	●	●	●	●
SPP test		●										●	●	●	●	●	●
Ischemia status evaluation		●										●	●	●	●	●	●
Lower limb MRI test		○															
Third-party qualification verification		●															
Implantation of test device				●													
Echo test of test device implantation site					●	●											
Break drain removal					●												
Questionnaire survey							●								●	●	●
Extraction of test device								●								●	
Biotube quality evaluation								●	●								
Biotube sample storage								●	○							●	
Biotube transplantation									●								
Concomitant medications/combined therapies																●	●
Adverse events																●	●
Malfunctions																●	

●: mandatory ○:optional or possible ▲: both or either △: both or either if possible

6.5.1 Observation, inspection and investigation details

The principal investigator or clinical trial co-investigator will investigate and record the following items.

(1) Subject background

[Investigation items]

Subject identification code, date of consent, sex, date of birth, age, height, weight, primary disease (disease causing lower limb ischemia), lower limb targeted for biotube transplantation (hereafter referred to as the "target lower limb"), evaluation of the target lower limb, medical history (those cured at the time of eligibility confirmation) and presence or absence of complications (those not cured at the time of eligibility confirmation), and their details, pregnancy test, nutritional status, and status of the site where the test device is to be implanted.

[Survey period]

At the time of confirming eligibility

[Survey method]

The survey will be conducted through interviews and examinations. If it is difficult to measure height using a height gauge due to the subject being unable to maintain an upright position, simple measurements using a tape measure will be accepted. If the subject is a woman of childbearing potential, a pregnancy test will be conducted to confirm that she is not pregnant (the method of testing is not required).

[CRF contents]

Subject identification code, consent acquisition date, sex, date of birth, age (at the time of consent acquisition), date of height measurement, height [cm] (integer: rounded to the nearest tenth), date of weight measurement, weight [kg] (to the nearest tenth: rounded to the nearest tenth), primary disease, target limb (right or left), evaluation of target limb (presence or absence of history of revascularization including endovascular treatment and details thereof, presence or absence of amputation and details thereof, length of biotube required for bypass surgery), medical history, presence or absence of complications and disease name, pregnancy test (whether or not it was performed, date of test, result of test, reason for not performing it), nutritional status (CONUT score), status of planned site for implantation of the study device (planned site, planned site for implantation, details of abnormality including skin abnormality)

(2) Subjective and objective findings

[Survey items]

Subjective and objective findings (findings associated with severe limb ischemia: pain, numbness, heat, tenderness, swelling, wounds, exudate, ulcers/erosions, fever, and other notable findings)
[Survey period]
At the time of eligibility confirmation, Visit 2 (before implantation of the test device), Visit 5, Visit 6 (before removal of the test device), Visit 7 (before implantation of the biotube), Visit 8, Visit 9, Visit 10, Visit 11, Visit 12, Visit 13, and at the time of discontinuation
[Survey method]
Confirm by examining the patient. Also, take a photograph of any wounds caused by the primary disease.
[CRF contents]
Presence or absence of subjective and objective findings and abnormalities associated with severe lower limb ischemia: presence or absence of pain, numbness, heat sensation, tenderness, swelling, wounds, exudate, ulcer/erosion formation, fever (37.5°C or higher within 24 hours), and other notable findings) and their details [including size (length and width) for wounds)], presence or absence of photograph of wound site, date of photograph, photograph of wound site (if there is a wound)

(3) Vital signs
[Survey items]
Temperature, blood pressure, pulse rate
[Survey period]
At the time of eligibility confirmation, Visit 2 (before implantation of the test device), Visit 5, Visit 6 (before removal of the test device), Visit 7 (before implantation of the biotube), Visit 8, Visit 9, Visit 10, Visit 11, Visit 12, Visit 13, at the time of discontinuation
[CRF contents]
Temperature [ºC] (to one decimal place), blood pressure (systolic and diastolic) [mm Hg] (integer), pulse rate [beats/min]

(4) Blood tests (hematological tests, blood biochemistry tests, blood coagulation tests)
[Survey items]

1. Hematological tests: white blood cell count (differential), red blood cell count, hemoglobin content, hematocrit value, platelet count, total lymphocyte count (only at the time of eligibility confirmation)
2. Blood biochemistry tests: total protein, albumin, fasting blood glucose, AST (GOT), A triglyceride, LDL cholesterol, LDH, BUN, creatinine, Na, K, Cl, CRP, total cholesterol (only at the time of eligibility confirmation)
3. Blood coagulation tests: PT, PT-INR, APTT

[Survey period]

At the time of eligibility confirmation, Visit 2 (before implantation of the test device), Visit 5, Visit 6 (before removal of the test device), Visit 7 (before implantation of the biotube), Visit 8, Visit 9, Visit 10, Visit 11, Visit 12, Visit 13, at the time of discontinuation

[CRF contents]

Each test value

(5) Lower limb arterial echocardiography

[Survey items]

Lower limb arterial echocardiography

[Survey period]

At the time of eligibility confirmation, Visit 5, Visit 10, Visit 11, Visit 12, Visit 13, and at the time of discontinuation

[CRF contents]

Arterial echocardiography findings of the target lower limb [presence or absence of infarction or stenosis in the artery of the target lower limb, presence or absence of new infarction or stenosis in the artery of the target lower limb (findings, presence or absence of abnormal findings)], diameter and presence or absence of stenosis of bypass graft after transplantation (findings, presence or absence of abnormal findings)

(6) Upper and lower limb venous ultrasound examination

[Survey items]

Upper and lower limb venous ultrasound examination

[Survey period]

At the time of eligibility confirmation (for dialysis patients, upper limb examination is not necessary)

[CRF contents]

Upper and lower limb venous ultrasound examination findings (location, length and presence or absence of varicose veins, presence or absence of autologous veins that can be used for arterial bypass surgery)

(7) Lower limb CT angiography, lower limb arteriography
[Survey items]
Lower limb CT angiography, lower limb arteriography
[Survey period]
At the time of eligibility confirmation, Visit 10, Visit 13 (performed if possible)
[Survey method]
Perform lower limb CT angiography and/or lower limb arteriography for the target lower limb.
[CRF contents]
Test method, presence or absence of findings related to ischemia in the target lower limb, and findings

(8) Lower limb X-ray examination
[Survey items]
Lower limb X-ray examination
[Survey period]
At the time of eligibility confirmation, Visit 10, Visit 11, Visit 12, Visit 13, and at the time of discontinuation
[CRF contents]
Presence or absence of X-ray findings of the target lower limb, findings, and presence or absence of osteomyelitis

(9) ABI (Ankle Brachial Index) Test
[Survey items]
ABI Test
[Survey period]
At the time of eligibility confirmation, Visit 10, Visit 11, Visit 12, Visit 13, and at the time of discontinuation
[CRF contents]
ABI measurement values (right, left)

(10) SPP (skin perfusion pressure) test

[Survey items]
SPP test
[Survey period]
At time of eligibility, Visit 10, Visit 11, Visit 12, Visit 13, and at time of discontinuation
[CRF contents]
SPP of target limb [mm Hg] (integer)

(11) Ischemic state evaluation
[Survey items]
Scores in each category of WIfI and GLASS classification
[Survey period]
WIfI classification: At the time of eligibility confirmation, Visit 10, Visit 11, Visit 12, Visit 13, at the time of discontinuation
GLASS classification: At the time of eligibility confirmation
[CRF content]
Scores in each category of WIfI and GLASS classification for the target limb

(12) Lower limb MRI examination
[Survey item]
Lower limb MRI examination
[Survey time]
At the time of eligibility confirmation (performed if osteomyelitis is suspected in. the target lower limb and examination is possible)
[CRF content]
Presence or absence of MRI examination of the target lower limb and findings, presence or absence of osteomyelitis

(13) Eligibility check by a third party
[Survey item]
Status of eligibility check by a third party
[Survey period]
At the time of eligibility confirmation
[CRF contents]
Whether or not a third-party eligibility check was conducted and the details (including the date and person who checked)

(14) Implantation of the test device

[Survey item]

Compliance with the implantation procedure of the test device

[Survey period]

Visit 2

[Survey method]

Investigate compliance with "6.4.2 Procedures for using the test device BTM1".

[CRF content]

Compliance with the implantation procedure of the test device (number of implants and location (including left and right), size)

(15) Echo examination of the test device implantation site (including break drain removal)

[Survey item]

Echo examination of the test device implantation site

[Survey period]

Visit 3 (before break drain removal), Visit 4

[Survey method]

Conduct an ultrasound examination before and approximately one week after the break drain removal to confirm the presence or absence of blood or exudate at the test device implantation site.

[CRF content]

Echo examination findings at the test device implantation site (presence or absence of blood or exudate, BTM1 number with findings, including the date of break drain removal)

(16) Questionnaire survey

[Survey items]

Details regarding implantation of the test device and QOL after biotube implantation

[Survey period]

Visit 5, Visit 13, and at the time of discontinuation

[Survey method]

A questionnaire survey was conducted on subjects after removal of the test device and 12 weeks after implantation of the biotube.

[CRF contents]

Before implantation of the test device (anxiety and expectations regarding the treatment of implanting the test device, about creating one's own blood vessels in one's own body, other opinions), after implantation of the test device (discomfort and anxiety at the time of implantation, presence or absence of pain such as pain and itching, impact on daily life and inconvenience, difference from imagination before implantation, change in feelings about the device during the implantation period, discomfort and anxiety after removal, other opinions), after implantation (condition of the implanted area, pain and itching at the implanted area, wound pain and improvement in walking, impact on daily life, other opinions)

(17) Removal of the test device

[Survey item]

Compliance with the removal procedure of the test device

[Survey period] Visit 6

[Survey method]

Investigate compliance with "6.4.2 Procedure for using the test device BTM1".

[CRF description]

Compliance with the removal procedure of the test device (formation of subcutaneous hematoma, accumulation of subcutaneous exudate, excessive adhesion, infection, rust, disintegration, damage of BTM1, deterioration and damage of the internal core)

(18) Quality evaluation of biotubes (including preservation of samples for histopathological evaluation of biotubes)

[Survey items]

Information on quality evaluation of biotubes and preservation of samples for histopathological evaluation

[Survey period] Visit 6, Visit 7 (performed if necessary) (see "6.4.2 Procedures for using the test device BTM1")

For Visit 7, if the biotube is not transplanted on the same day as Visit 6 and the biotube is temporarily stored, it will be performed immediately before transplantation of the biotube. (For preservation of samples for histopathological evaluation, it will be performed if the amount of sample is sufficient)

[CRF description contents]

Total length, length, presence or absence of tears or holes (if present, length), presence or absence of thickness/extremely thin parts (if present, length), presence or absence of uneven parts (if present, length), presence or absence of leakage, tensile strength, pressure resistance, presence or absence of preservation of samples for histopathological evaluation, usable length, presence or absence of preservation of biotubes, evaluation date, presence or absence of abnormalities in appearance (decomposition, decay, abnormal odor, discoloration, etc.), presence or absence of preservation of samples for bacterial testing

(19) Biotube transplantation

[Survey items]

Information on biotube transplantation

[Survey period]

Visit 7

[CRF contents]

Whether or not biotube transplantation was performed, transplant date, confirmation date of transplant postponement, transplant date of transplant postponement, graft used, combination method, graft (biotube number), length of biotube used, autologous vein (site, length), artificial blood vessel (product name, length), pressure resistance, reason for transplant discontinuation, whether or not biotube was connected, date of connection, length of biotube after connection, whether or not it was pressure resistant (if not, reason), whether or not biotube was repaired

(20) Concomitant medications and concomitant therapies

Concomitant medications and concomitant therapies refer to all medications used or all treatments and procedures performed after obtaining consent. However, implantation of a test device and biotube implantation are excluded.

[Survey items]

Target medications, treatments and procedures

[Survey period]

From confirmation of eligibility to Visit 13 or discontinuation.

[CRF content]

Presence or absence of concomitant medications (if there are concomitant medications, the name of the drug (brand name or generic name), route of administration, single dose, frequency of administration, duration of administration, reason for administration), presence or absence of concomitant therapy (if there are

concomitant therapies, the name of the therapy, start date, end date, purpose of treatment)

(21) Adverse events

[Survey items]

Whether or not an adverse event occurred, and if so, information related to the adverse event

[Survey period]

From implantation of the study device until Visit 13 or discontinuation.

[CRF content]

See "7. Safety evaluation"

(22) Malfunctions

[Investigation items]

Whether or not a malfunction occurred, and if so, information related to the malfunction.

[Investigation period]

From Visit 2 (when the test device is implanted) to Visit 13 or cancellation. Although the biotube is not a test device, in consideration of the characteristics of this clinical trial, malfunctions of the biotube will also be investigated. The investigation period for the biotube will be from Visit 6 (when the test device is removed) to Visit 13 or cancellation.

[CRF content]

See "7. Safety evaluation".

6.5.2 Examinations and observations after cancellation

If the study is discontinued for any reason after registration, it will be treated as a discontinued case. If the study is discontinued after implantation of the test device, the examinations and observations listed in "6.5 Observation and Examination Schedule" and "6.5.1 Observation, Investigation, and Examination Details" will be carried out. In addition, if follow-up is required due to an adverse event, the patient will be followed up until recovery or improvement in principle.

7. Safety assessment

7.1 Clinical test values

The investigator or subinvestigator will compare the test values and measurements taken after implantation of the investigational device with the results of tests taken after obtaining consent, and determine whether there are any abnormal changes. When determining whether there are any abnormal changes in clinical test values, the National Cancer Institute Common Terminology Criteria for Adverse Events (NCI CTCAE v5.0) should be used as a reference, and if there is no corresponding item, the Grade (severity) should be evaluated with reference to Table 7.1.

If abnormalities in clinical test values are observed, the investigator or subinvestigator will determine whether the abnormality should be treated as an adverse event with reference to the following items.

(1) When clinical trial treatment is discontinued due to abnormal laboratory test values
(2) When drug or surgical intervention is performed to improve abnormal laboratory test values
(3) When a correlation with clinical symptoms is found
(4) When a correlation with a serious adverse event is found
(5) When the principal investigator or co-investigator determines that an adverse event should be treated as such, other than the above (1) to (4)

Table 7.1 Grade Classification (Severity) Criteria for Adverse Events

Grade	severity
1	Mild. Asymptomatic or mild symptoms. Clinical or laboratory findings only. No treatment required.
2	Moderate. Minimal/local/non-invasive treatment required. Age-appropriate limitation in ADL other than self-care.
3	Severe or medically significant, but not immediately life-threatening. Hospitalization or prolonged hospitalization required. Disabling. Limitation in self-care ADL.
4	Life-threatening. Urgent intervention required.
5	Death due to adverse event (AE).

7.2 Adverse Events

7.2.1 Definition of Adverse Events

Adverse events are any undesirable or unintended disease or disorder or its symptoms (including abnormalities in clinical test results) that occur in subjects treated with the investigational device, regardless of whether they are causally related to the investigational device. Adverse events will be collected from the time of treatment with

the investigational device until the end of the observation period or discontinuation of each subject (excluding the follow-up period), and the severity will be determined according to the National Cancer Institute Common Terminology Criteria for Adverse Events (NCI CTCAE v5.0). If there is no applicable item, the grade will be determined with reference to Table 7.1.

7.2.2 Description of adverse events

In principle, the name of the adverse event is recorded in the case report form as the diagnosis or disease name. If the diagnosis or disease name cannot be specified or if the investigator or co-investigator determines that it is appropriate not to use the diagnosis or disease name, the clinical symptoms or signs (including abnormal laboratory test values) are recorded as the adverse event name in the case report form.

For all adverse events that occurred, the investigator or co-investigator should record the name of the adverse event, the date of onset, the date of outcome, the severity, seriousness, the reason for determining that the adverse event was serious (see 7.2.3 Serious Adverse Events (1) to (7)), the causal relationship with the investigational device, the causal relationship with the biotube, the outcome (recovered, remitted, with aftereffects, not recovered, death), the treatment taken for the investigational device, the treatment taken for the biotube, the treatment taken to treat the adverse event, and any comments in the adverse event section of the case report form.

7.2.3 Serious adverse events

Adverse events that fall under any of the following categories will be treated as serious adverse events.

(1) Death
(2) Potential for death
(3) Permanent or significant disability or dysfunction
(4) Potential for disability
(5) Requires hospitalization or extended hospitalization for treatment
(6) Causes congenital abnormality
(7) Other serious medical conditions

However, hospitalizations for the sole purpose of carrying out treatment or examinations that were planned before participation in the clinical trial (before consent was obtained) during the clinical trial (scheduled surgery, examinations, etc.), hospitalizations for purposes other than treatment or examinations associated with an

adverse event (health checkups, etc.), and no undesirable medical events will be treated as serious adverse events.

7.2.4 Determination of the severity of adverse events

The severity of adverse events is classified as follows:

(1) Serious

(2) Non- serious

7.2.5 Actions taken to treat adverse events

Actions taken on subjects to treat adverse events are classified as follows:

(1) Yes (description: drug therapy, other (details))

*If yes, provide detailed information in the section on concomitant medications and therapies in the case report.

(2) No

7.2.6 Actions taken on the test device

If an adverse event occurs, actions taken on the test device are classified as follows:

(1) No

When the use of the test device was not discontinued

(2) Discontinued

When the use of the test device was discontinued (the test device was removed)

7.2.7 Outcome of adverse events

The outcomes of adverse events are classified as follows:

(1) Recovered: The adverse event has disappeared or returned to its original state

(2) Relieved: The adverse event has not completely recovered, but has almost disappeared or returned to its original state

(3) Sequelae: The adverse event has recovered to its original state, but sequelae remain

(4) Unrecovered: The adverse event is ongoing

(5) Died: The subject died as a result of the adverse event

For adverse events that have not yet recovered as of the end of the observation period for each subject, follow-up will be conducted until recovery or remission occurs as far as possible. However, if the principal investigator or sub-investigator determines that follow-up is not necessary, such as in the case of irreversible events, follow-up may end on the end of the observation period for the subject. In that case,

the reason for determining that follow-up is not necessary should be stated in the comments section of the case report form.

In addition, the causal relationship of adverse events to the test device will be determined on a three-level scale according to the criteria in Table 7.2.7 below, and recorded in the case report form. Based on Table 7.2.7, events that fall into either the category of "Relationship cannot be denied" or "Related" in terms of relevance to the test device are considered to be events for which a causal relationship with the test device cannot be denied. If the event falls into the category of "Not related," the reason for this judgment should be written in the comments section of the case report.

Table 7.2.7 Criteria for determining causal relationship with the test device

Not related	If the relationship with the test device can be clearly denied
The relationship cannot be denied	If the relationship with the test device is suspected
Related	If the relationship with the test device is clearly recognized

7.3 Malfunctions

7.3.1 Malfunctions

Malfunctions refer to problems with the specifications of the subject device, defects or malfunctions of the subject device, or problems with the procedure. Information on malfunctions that occur during the clinical trial will be collected as needed. In addition, considering the nature of this clinical trial, we will continue to investigate any malfunctions of the biotube even after the test device is removed.

7.3.2 Recording of Malfunction Information

If a malfunction occurs in the study device/biotube, the principal investigator or co-investigator will record the details of the malfunction in the case report. The safety of the malfunction will be determined based on Table 7.3.2.

Table 7.3.2 Fault safety level

Safety level 1	Affects subjects and surgeons, etc.
Safety level 2	May affect subjects and surgeons, etc.
Safety level 3	Does not affect subjects and surgeons, etc.

7.4 Responses to Adverse Events and Malfunctions

(1) If the investigator or co-investigator becomes aware that treatment for an adverse event is necessary, he/she shall inform the subject of the event.

(2) In the event of an adverse event, the investigator or co-investigator shall take appropriate measures, pay attention to ensuring the safety of the subject, and endeavor to determine the cause.

(3) In principle, the investigator or co-investigator shall continue to monitor the symptoms or abnormal changes in clinical test values as much as possible and confirm the outcome until the event has disappeared or returned to the state before the start of the clinical trial, or until it is determined that the event is not a clinical problem.

7.5 Reporting of serious adverse events and malfunctions that may lead to serious adverse events

If an adverse event occurs in a subject during a clinical trial and the investigator or co-investigator judges the adverse event to be serious, the adverse event will be handled according to the following procedures.

(1) Report from the investigator to the head of the clinical trial institution and the coordinating investigator

The investigator shall report the adverse event information to the head of the clinical trial institution as soon as possible, regardless of causality, and shall also report it to the coordinating investigator.

(2) Notification by the coordinating investigator to the investigators and investigational device providers of each medical institution

The coordinating investigator shall confirm the contents of the adverse event report received from the investigator and notify the investigators and investigational device providers of each other medical institution of the adverse event information.

(3) Discussion between the Principal Investigator and the Coordinating Investigator

The Principal Investigator at each medical institution shall review the report received from the Coordinating Investigator, consult with the Principal Investigator as necessary, and report his/her opinion as the Principal Investigator (including the need to report to the Minister of Health, Labour and Welfare) to the Coordinating Investigator.

(4) Convening an Efficacy and Safety Evaluation Committee

If the Coordinating Investigator determines that the adverse event is subject to reporting under Article 273 of the Enforcement Regulations of the Pharmaceuticals and Medical Devices Act, he/she shall convene an Efficacy and Safety Evaluation Committee and request a decision on whether or not to continue the clinical trial.

If the Efficacy and Safety Evaluation Committee determines whether or not to continue the clinical trial, it shall notify the Coordinating Investigator, who shall notify the Principal Investigator.

(5) Report to the Minister of Health, Labour and Welfare
If the principal investigator determines that the defect or other problem should be reported to the Minister of Health, Labour and Welfare as stipulated in the "Act on Ensuring Quality, Efficacy and Safety of Pharmaceuticals, Medical Devices, etc." and its Enforcement Regulations, the coordinating investigator will prepare a report in the designated format and submit it to the PMDA.

(6) Report to the head of the medical institution conducting the study
When a serious adverse event that occurred at another medical institution is reported to the Minister of Health, Labour and Welfare, the principal investigator shall report the contents of the report in (5) obtained from the coordinating investigator to the head of the medical institution as soon as possible.

(7) Response when additional information is obtained
When the principal investigator of the medical institution where the adverse event occurred obtains additional information on the adverse event, he/she shall report it to the head of the medical institution as soon as possible and also report it to the coordinating investigator and the investigational device provider. The handling of the additional information shall conform to the procedures in (1) to (6) above, and shall report it to the PMDA if necessary.

7.6 Provision of new information

If the investigator obtains any information that may adversely affect the safety of the subjects, affect the conduct of the clinical trial, or change the approval of the IRB or other such organization regarding the continuation of the clinical trial, he/she shall promptly report this in writing to the head of the medical institution conducting the trial, and shall promptly revise the explanatory document if necessary. The investigator or co-investigator shall notify the subject of this and ensure appropriate treatment and follow-up procedures.

8. Efficacy evaluation

8.1 Primary evaluation item

Ability of forming biotubes by the test device

When the biotube is removed from the core and inserted into the straightening rod, the presence or absence of abnormalities such as inflammation is checked and the formation

state is evaluated visually. After soaking in alcohol, the total length of the biotube is measured while it is inserted into the straightening rod. If the biotube is formed in segments, the length of each segment and the total length are measured. In addition, the thickness and uniformity of the tissue are visually examined. The straightening rod is removed, and the lumen is pressurized with saline using a syringe to check for damage and leakage. A portion is taken and the breaking strength is measured using a tensile tester. Histopathological observation is performed at a later date to examine the thickness, inflammation, and collagen structure. Note that this evaluation item is intended to evaluate the quality of the formed biotube from multiple perspectives, but in each case, if bypass surgery can be performed using only the biotube as a bypass graft (including cases where fragments of the biotube are used together), this evaluation item is considered to be "achieved."

8.2 Secondary Evaluation Items

The following evaluation items (1) to (8) will be evaluated secondary to the above.

(1) Procedural success regarding implantation and removal of the test device
At the time of implantation, the subcutaneous pocket will be visually evaluated to ensure that it is large enough to create a sufficient size subcutaneous pocket, that the test device can be inserted without damaging or disintegrating the device, and that the wound can be closed while controlling subcutaneous bleeding. During the implantation period, the skin will be visually evaluated to ensure that there are no abnormalities such as hematoma formation or accumulation of exudate. At the time of removal, the degree of adhesion between the test device and the surrounding tissue will be confirmed, and it will be visually evaluated that the test device can be removed without damaging or disintegrating the device while controlling bleeding.

(2) Procedural success of bypass surgery including peripheral anastomosis using a biotube formed by the test device It will be visually evaluated whether the biotube can be sutured without damage or cutting using the same technique as a bypass surgery using a normal vein. It will be visually evaluated whether bleeding from the anastomosis site can be controlled, and there is no blood leakage, tearing, or swelling from the biotube wall. After releasing the vascular clamp, pulsatile blood flow is confirmed and the flow rate is measured with a flowmeter to evaluate peripheral revascularization. Note that "technical success" in this evaluation item refers to cases where bypass surgery was performed using only the biotube as a bypass graft (including cases where fragments of the biotube were joined together), and does not include cases where bypass surgery was performed by joining an autologous vein or an artificial blood vessel.

(3) Patency of the biotube at 12 weeks after implantation

During the follow-up period, blood flow in the biotube is evaluated by lower limb arterial echocardiography. If possible, the shape of the biotube is observed by lower limb CT angiography or lower limb arteriography to evaluate the presence or absence of abnormalities such as stenosis or swelling.

(4) Improvement of symptoms (improvement of ischemic pain, improvement of wounds)

The pain improvement rate, wound improvement rate, wound area reduction rate, and granulation formation rate are evaluated at the time of eligibility confirmation, at the time of biotube implantation, and 1, 4, 8, and 12 weeks after biotube implantation. In addition, the WIfI score is also evaluated at the time of eligibility confirmation, and 1, 4, 8, and 12 weeks after biotube implantation.

(5) Avoidance of major amputation

The avoidance rate of major amputation of the target lower limb during the follow-up period will be examined.

(6) Implementation rate of additional treatment during implantation of the study device and after biotube implantation

The rate of revascularization other than bypass surgery using the biotube will be evaluated from implantation of the study device to the end of the follow-up period.

(7) QOL during implantation of the study device and after implantation of the biotube

The results of a questionnaire conducted approximately 6 weeks after implantation of the study device will be used to evaluate the presence or absence of discomfort at the implantation site of the study device and the presence or absence of impact on daily life. In addition, the results of a questionnaire conducted at the end of the follow-up period will be used to evaluate the QOL after biotube implantation.

(8) Bypass graft diameter after bypass surgery

During the follow-up period, the diameter of the biotube used in the bypass surgery (including artificial blood vessels or autologous veins, if used) will be investigated and evaluated over time by lower limb arterial echocardiography.

9. Statistical Analysis

The main analysis policy is shown below, but more technical and detailed analysis items and methods will be described in the statistical analysis plan. The statistical analysis plan will be prepared before data fixation, and the analysis will be performed after data fixation. The handling of individual cases and data will be decided at the case review meeting before data fixation.

9.1 Analysis population

(1) Efficacy analysis subjects

1) FAS (Full Analysis Set)

The analysis population consisting of registered subjects excluding the following subjects will be the FAS:

- Subjects who were not critical limb ischemia patients targeted by this clinical trial
- Subjects who have never used the investigational device
- Subjects with no efficacy data after registration

2) PPS (Per Protocol Set)

The PPS will be the analysis set consisting of subjects from the FAS, excluding the following subjects:

- Subjects who do not meet the inclusion criteria
- Subjects who meet the exclusion criteria
- Subjects who meet other regulations in the clinical trial protocol that may affect the efficacy evaluation.

The case review committee will also discuss how to handle discontinued cases.

(2) Safety Analysis Set

The analysis set consisting of the subjects who were enrolled, excluding the following subjects, will be the Safety Analysis Set (SAS):

- Subjects who have never used the test device
- Subjects who have no safety data after enrollment

9.2 Analysis of efficacy

The primary endpoint will be the ability of the test device to form a biotube. As this is an exploratory clinical trial, it will be evaluated comprehensively and from multiple angles, including the procedures and conditions related to the implantation and removal of the test device, and the performance of the biotube formed by the test device.

9.3 Safety Analysis

To evaluate safety, the following will be evaluated and the incidence of adverse events and malfunctions will be analyzed.

(1) Occurrence of inflammation, tumor formation, and death whose relationship to the test device cannot be denied during the period of subcutaneous implantation of the test device

(2) Biocompatibility of the implanted biotube (inflammation, tumor formation, etc.)

(3) Rupture of the implanted biotube
(4) Occurrence of death whose relationship to the implanted biotube cannot be denied
(5) Occurrence of death within 12 weeks after implantation of the biotube
(6) Occurrence of other adverse events and malfunctions

9.4 Interim Analysis

An interim analysis will be conducted when the number of cases 12 weeks after biotube implantation reaches 6.

The interim analysis will evaluate the primary endpoint of biotube formation ability by the test device and the secondary endpoint of patency of the biotube at 12 weeks after implantation.

9.5 Setting the number of cases

The target number of cases to be enrolled was set at 12 cases (no control group) based on the following criteria.

[Rationale for setting]

The test device is available in two sizes, large and small, and the implantation location is selected from four locations: chest, abdomen, buttocks, or thigh. The implantation location and number are determined by the principal investigator or clinical investigator based on the conditions deemed most suitable for the subject, so the number of implants per implantation location cannot be distributed evenly. However, in order to examine the same location, size, and other conditions in multiple cases, a minimum of 12 cases are necessary. This clinical trial is an exploratory study, and taking into consideration feasibility and interim analysis, the target number of registered cases was set at 12.

10. Quality control and quality assurance of clinical trials

To ensure the quality of this clinical trial, the investigator and the medical institution will carry out quality control of the clinical trial in accordance with standard operating procedures.

10.1 Quality management in this clinical trial

In clinical trials, quality control and quality assurance play an important role in ensuring the quality of the clinical trial. In order to more effectively utilize quality control and quality assurance to ensure the quality of the clinical trial, this clinical trial will establish an appropriate system based on the Notification of the Director of the Pharmaceutical Evaluation and Control Division, Pharmaceutical and Food Safety Bureau, Ministry of

Health, Labour and Welfare, dated July 5, 2019 (PSEHB/PDA Notification No. 0705-5), "Basic principles of quality management in clinical trials," and will implement quality management for the clinical trial.

10.2 Direct Inspection of Source Documents, etc.

The investigator and the head of the medical institution conducting the clinical trial must accept monitoring, audits, and investigations by the IRB and domestic and international regulatory authorities, and must provide direct inspection (including copying) of all clinical trial-related records, such as source documents. Details are in accordance with the standard operating procedures. Note that subjects agree to direct inspection by signing the consent form.

10.3 Monitoring

The monitoring personnel designated by the coordinating investigator will follow the separately stipulated "Monitoring Procedures" and periodically monitor the medical institution to confirm that the clinical trial is being conducted in accordance with all of the contents of this clinical trial protocol and the "Ministry of Health, Labor and Welfare Ordinance on Standards for the Conduct of Clinical Trials of Medical Devices (Medical Device GCP)" (Ministry of Health, Labor and Welfare Ordinance No. 36 of 2005: Medical Device GCP Ordinance).

10.4 Audits

The auditor designated by the coordinating investigator will conduct audits at the medical institution conducting the study and the contracted development institution in accordance with the separately stipulated "Procedures for Conducting Audits."

10.5 Cooperation with Monitoring and Audits

The principal investigator, the head of the medical institution conducting the study, and the person in charge of the contracted development institution must cooperate with monitoring and audits.

11. Ethics and Compliance with GCP

11.1 Ethical Conduct of the Clinical Trial

This clinical trial will be conducted in accordance with the spirit of the Declaration of Helsinki. It will also be conducted in compliance with the standards set forth in Article 14, Paragraph 3 and Article 80-2 of the Pharmaceuticals and Medical Devices Act, relevant

laws and regulations such as the Medical Device GCP Ministerial Ordinance, and this clinical trial protocol.

11.2 Explanation to subjects and obtaining consent

(1) The investigator shall prepare an explanatory document to be used to obtain consent to participate in the clinical trial from the subject, and shall revise it if necessary.
(2) The prepared or revised document shall be approved in advance by the IRB.
(3) The explanatory document shall not contain any statement that may cause the subject to waive his/her rights, or any statement that may cause the subject to avoid legal responsibility of the investigator or sub-investigator, the medical institution, or the person conducting the clinical trial.
(4) The explanatory document shall include the following items:
   1) That the clinical trial involves research.
   2) The purpose of the clinical trial.
   3) The name and contact information of the investigator.
   4) The method of the clinical trial.
   5) Anticipated clinical benefits and risks or inconveniences.
   6) The expected duration of the subject's participation in the clinical trial.
   7) Availability of other treatment methods for the target patient, and the important anticipated benefits and risks of those treatment methods.
   8) Participation in this clinical trial is at the subject's own voluntary discretion, and the subject may refuse or withdraw from participation in this clinical trial at any time. Furthermore, the subject will not be disadvantaged by refusal or withdrawal, and will not lose any benefits that would be available to the subject if he or she did not participate in this clinical trial.
   9) The monitors, auditors, IRBs, and regulatory authorities shall have access to the medical source documents. In doing so, the subjects' confidentiality shall be maintained. The subjects' signature on the consent form shall constitute permission for access.
   10) The subjects' confidentiality shall be maintained even if the results of the clinical trial are made public.
   11) A consultation desk at the medical institution where the subjects should inquire or contact if they wish to obtain further information regarding the clinical trial and their rights, or if a health injury related to the clinical trial occurs.
   12) The compensation and treatment that the subjects will be entitled to in the event of a health injury related to the clinical trial.

13) The number of subjects expected to participate in the clinical trial.
14) Any information that may affect the subjects' willingness to continue participating in the clinical trial shall be promptly communicated to the subjects.
15) The conditions or reasons for discontinuing participation in the clinical trial.
16) The details of any expenses that the subjects will be required to pay.
17) If any money or other payments will be made to the subjects, the details of such payments (e.g., agreements for calculating payment amounts).
18) Matters that subjects must observe.
19) The type of IRB that will investigate and deliberate on the appropriateness of the clinical trial, matters to be investigated and deliberated by the IRB, and other IRB-related matters related to the clinical trial.
20) Information such as the IRB procedure manual, list of committee members, and minutes of committee meetings will be made public and available for viewing.
21) Other matters necessary for protecting the human rights of subjects.

11.3 Method of obtaining consent

(1) Prior to participation in the clinical trial, the investigator or co-investigator shall provide the subject with a sufficient explanation using an explanatory document approved by the IRB, and then obtain the subject's voluntary written consent to participate in the clinical trial.
(2) When providing the explanation, the investigator or co-investigator shall give the subject an opportunity to ask questions and the time necessary to decide whether or not to participate in the clinical trial before obtaining consent. Furthermore, at that time, the investigator, co-investigator, or clinical collaborator acting as a supplementary explanation provider shall answer all questions to the subject's satisfaction.
(3) The consent document shall be signed and dated by the investigator or co-investigator who provided the explanation and the subject. If a clinical collaborator provides supplementary explanation, the clinical collaborator shall also sign and date the document.
(4) In principle, the principal investigator or co-investigator will provide the subject with a signed and dated copy of the consent form and an explanatory document before the subject participates in the clinical trial.
(5) The original of the consent form will be kept at the medical institution conducting the clinical trial.

11.4 Revision of the Information Document

(1) If new important information that may be relevant to the subject's or proxy's intention to consent (usually information that requires revision of the information document) is obtained, the investigator shall promptly revise the information document based on that information and obtain approval from the IRB.
(2) The investigator or co-investigator shall use the revised informed consent document to provide further explanation to the subject and obtain the subject's voluntary written consent to continue participating in the clinical trial.
(3) The investigator or co-investigator shall provide the subject with a newly signed and dated copy of the informed consent document and the newly dated informed consent document. The original of the informed consent document shall be kept at the medical institution conducting the clinical trial.

11.5 Institutional Review Board

Prior to the implementation of this clinical trial, approval of the IRB will be obtained regarding the appropriateness of conducting this clinical trial from the standpoints of ethical, scientific, and medical appropriateness.

11.6 Matters concerning the protection of human rights of subjects

When selecting subjects, the principal investigator or clinical investigator shall carefully consider the appropriateness of requesting them to participate in the clinical trial, taking into consideration the subject's health condition, symptoms, age, ability to consent, dependency on the principal investigator or clinical investigator, and participation in other clinical trials, based on the inclusion and exclusion criteria from the perspective of human rights protection. Subjects shall be identified using subject identification codes when registering subjects, submitting samples to external parties, and creating case report forms. At the medical institution conducting the clinical trial, a correspondence table shall be created between personal identification information and subject identification codes, and the correspondence table shall be appropriately managed at each medical institution. Sufficient consideration shall be given to protecting the privacy of subjects' names, diseases, etc. when directly viewing source documents related to the implementation of the clinical trial and consent documents of subjects, and when publishing the results of the clinical trial.

12. Case report form

12.1 How to write

(1) The principal investigator or co-investigator is responsible for the contents and corrections of the case report form. After each observation and examination of each

subject is completed, the case report form should be written within two weeks. For the method of writing and correcting the case report form, follow the guide for writing case report forms.

(2) If source documents exist and their objectivity can be guaranteed, the clinical trial collaborator may transcribe the source documents into the case report form.

(3) The principal investigator shall check and confirm the contents of the case report form before signing it.

(4) The principal investigator shall submit the case report form in accordance with the prescribed procedure.

(5) If there is a discrepancy between the case report form and the source documents, the principal investigator shall prepare a record explaining the reason and submit it in accordance with the prescribed procedure.

(6) With regard to the data described in the case report form, with regard to the items listed in "12.3 Identification of items for which the contents of the case report form should be used as source documents," if the principal investigator or subinvestigator directly enters the data in the case report form, the presence or absence of source documents is not an issue.

12.2 Source documents

Source documents refer to the documents that are the basis for the data described in the case report as a result of the clinical trial.

(1) Records regarding the subject's consent and provision of information

(2) Records that are the basis for creating the case report, such as medical records (including test slips and imaging test data), nursing records, etc.

(3) Records regarding the use of the test device

12.3 Identification of items for which the contents of the case report form should be used as source data

Among the data described in the case report form, the contents of the case report form shall be used as source data for the following items. However, if primary data such as medical records or worksheets exist, the primary data shall be used as source data.

・ Presence or absence of complications

・ Reasons for use of concomitant medications

・ Efficacy evaluation, comments on efficacy

・ Comments on safety

・ Presence or absence of concomitant medications or concomitant therapies

・ Presence or absence of discontinuation, reasons, and comments

・ Presence or absence of adverse events, seriousness, severity, measures taken for the test device, measures taken to treat adverse events, measures taken for the biotube, outcome date, outcome, presence or absence of causal relationship with the test device, causal relationship with the biotube, and comments

・ Presence or absence of malfunctions, type, safety, presence or absence of adverse events associated with malfunctions, measures taken for the test device, measures taken for the biotube, presence or absence of causal relationship with the test device, causal relationship with the biotube, outcome date, outcome, and comments

13. Clinical trial costs and compensation

13.1 Conflict of interest

This clinical trial will be conducted with research funding from AMED.
The test device BTM1 used in this clinical trial will be provided free of charge by Biotube Co., Ltd. under a joint research agreement. The World Medical Association Declaration of Helsinki requires that the subjects be fully informed of funding, sponsors, and conflicts of interest and that they be included in the research protocol, and therefore the conflicts of interest in this clinical trial will be described in the clinical trial implementation protocol. As a relevant matter, the principal investigator at Yokohama General Hospital, the medical institution where the clinical trial will be conducted, is an officer of Biotube Co., Ltd. and owns shares, but will be reviewed by the Oita University School of Medicine Clinical Research Conflict of Interest Management Committee and will appropriately manage conflicts of interest when conducting this clinical trial. In addition, each medical institution will also appropriately manage conflicts of interest. The method of conflict of interest management will be left to the standards of each medical institution.

13.2 Compensation for Health Damage and Insurance

If a subject suffers health damage resulting from the implementation of this clinical trial, the medical institution conducting the trial shall provide treatment and other necessary

measures, and the principal investigator shall provide appropriate compensation, except in cases where the damage is caused by the intentional or gross negligence of the medical institution conducting the trial or the subject. In addition, if liability for compensation arises due to health damage resulting from this clinical trial, the parties that caused the cause (the principal investigator, the medical institution conducting the trial, etc.) shall share the amount of compensation in proportion to their respective shares.

The coordinating investigator and the principal investigator shall take insurance and other necessary measures to fulfill their liability for compensation and indemnification.

13.3 Planned clinical trial costs and payments to subjects

Medical expenses during the clinical trial will be borne by the subject, except for the costs of the investigational device and treatments and examinations that the principal investigator has agreed to cover at the medical institution conducting the trial.

14. Deviations, changes, and revisions to the clinical trial protocol

When conducting this clinical trial, the investigator must submit the clinical trial protocol to the IRB via the head of the medical institution, and obtain approval from the head of the medical institution after obtaining prior approval from the IRB. This clinical trial will be conducted in compliance with this clinical trial protocol with the agreement between the investigator and the head of the medical institution.

14.1 Deviations or changes to the clinical trial protocol

(1) The investigator or co-investigator shall not make any deviations or changes to the clinical trial protocol without obtaining written approval based on prior review by the IRB.
(2) The investigator or co-investigator may make deviations or changes to the clinical trial protocol without obtaining written approval based on prior review by the IRB in medically unavoidable circumstances, such as to avoid an immediate danger to the subject. In such cases, the investigator shall immediately submit a document outlining the content and reasons for the deviation or change to the head of the medical institution conducting the study, and obtain approval from the IRB.

14.2 Revision of the Clinical Trial Protocol

(1) If the need arises for revision of the clinical trial protocol during the progress of this clinical trial, the investigator shall discuss the content of the revision with the coordinating investigator as necessary.

(2) If the investigator revises the clinical trial protocol or explanatory document, he/she shall promptly submit the revised clinical trial protocol and explanatory document to the head of the medical institution conducting the clinical trial.
(3) After obtaining approval from the IRB and the head of the medical institution conducting the clinical trial, the investigator shall conduct this clinical trial in compliance with the revised clinical trial protocol and the revised explanatory document.

15. Termination, Suspension, and Termination of Clinical Trials

The rules for partial and complete termination of this clinical trial are as follows. In either case, the results at the time of termination will be collected and analyzed. The termination criteria for individual subjects are described in "4.4 Termination Criteria."

15.1 Termination or Suspension of Clinical Trial

If a reason arises during the progress of this clinical trial that requires the entire clinical trial to be terminated or suspended, such as when the validity of the development of the investigational device is denied or when an unexpected serious adverse event is observed and the implementation of this clinical trial is judged to be medically or ethically impossible, the principal investigator shall consult with the coordinating investigator and promptly notify the head of the medical institution conducting the clinical trial, the regulatory authority, and the coordinating investigator (clinical trial coordinating office) of the termination or suspension of the clinical trial and details of the reason for the termination or suspension. The head of the medical institution conducting the clinical trial shall notify the IRB of the termination or suspension and provide a detailed explanation in writing. The principal investigator or coordinating investigator shall notify the subject of the termination and ensure appropriate treatment and follow-up.

15.2 Completion of the Clinical Trial

When the clinical trial is completed, the investigator shall notify the head of the medical institution in writing and report a summary of the results of the clinical trial in writing (clinical trial completion report). The head of the medical institution shall promptly notify the IRB in writing of the completion of the trial and report a summary of the results of the clinical trial based on the clinical trial completion report.

16. About the Efficacy and Safety Evaluation Committee

The Efficacy and Safety Evaluation Committee is established by the coordinating investigator for the purpose of discussing whether to continue the clinical trial or to change

the clinical trial protocol. In accordance with the "Procedures for the Efficacy and Safety Evaluation Committee," the committee evaluates the progress of the clinical trial, safety data, and important efficacy evaluation items.
Details of the timing and purpose of the meeting are as described in "3.4 Restrictions on Case Registration."

17. Retention of Records, etc.
    (1) The head of the medical institution shall retain documents or source materials related to the clinical trial that should be retained at the medical institution until the later of the following 1) or 2). However, if the investigator or the head of the medical institution requires longer retention, the retention period and method shall be discussed with the investigator or the head of the medical institution.
        1) The date of manufacturing and marketing approval for the investigational device (if development is discontinued, the date three years have passed since the date on which the decision to discontinue development was made)
        2) The date three years have passed since the discontinuation or completion of the clinical trial

    (2) The person who establishes the IRB shall retain the operating procedure manual, list of committee members (including the qualifications of each committee member), list of committee members' occupations and affiliations, submitted documents, meeting records, their summaries, letters, etc. until the later of the following 1) or 2). However, if the investigator requires longer retention, the retention period and method shall be discussed with the investigator.
        1) The date of marketing approval for the test device (if development is discontinued, the date three years have passed since the date of notification of the discontinuation of development)
        2) The date three years have passed since the discontinuation or completion of this clinical trial

    (3) The person who conducts the clinical trial himself/herself shall preserve documents or records related to the clinical trial that should be preserved until the later of the following 1) or 2).
        1) The date of marketing approval for the test device (if development is discontinued, the date three years have passed since the date of decision to discontinue development)

2) The date three years have passed since the discontinuation or completion of this clinical trial

18. Disclosure Arrangements

If the principal investigator or co-investigator intends to disclose information obtained from this clinical trial to an external organization such as an academic society, he/she must obtain prior written consent from the coordinating investigator and the provider of the subject device.

19. Clinical trial implementation structure

See attached sheet

20. References

1. Norgren L, Hiart WR, Dormandy JA, Nehier MR, Harris KA, Fowkes FG. Inter-Society Consensus for the Manegement of peripheral artery disease (TASC II). J Vasc Surg 2007; 45(Suppl): S5-67
2. Dua A, Lee CJ. Epidemiology of peripheral artery disease and critical limb ischemia. Tech Vasc scInteventional Rad 2016; 19: 91-5
3. Alamasri J, Adusumalli J, Asi N, Lakis S, Alsawas M, Prokop LJ et al. A systematic review and meta-analysis of revascularization outcomes of infrainguinal chronic limb-threatening ischemia. J Vasc Surg 2018; 68: 624-33
4. Bradbury AW, Adam DI, Bell J, Forbes JF, Fowkes FGR, Gillespie I, et.al. Bypass versus angioplasty in severe ischemia of the leg (BASIL) trial: an intention-to treat analysis of amputation-free and overall survival in patients randomized to a bypass-first or a balloon angioplasty-first revascularization strategy. J Vasc Surg 2010; 51: 5S-17S
5. Bradbury AW, Adam DI, Bell J, Forbes JF, Fowkes FGR, Gillespie I, et. al. Bypass versus angioplasty in severe ischemia of the leg (BASIL) trial: analysis of amputation free and overall survival by treatment received. J Vasc Surg 2010; 51: 18S-31S.
6. Iida O, Nakayama M, Yamauchi Y, Kawasaki D, Yokoi Y, Yokoi H et al. Endovascular treatment for infrainguinal vessels in patients with critical limb ischemia. OLIVE registry, a prospective, multicenter study in Japan with 12-month follow-up. Circ Cardiovasc Interv 2013; 6: 68-76
7. Iida O, Takahara M, Soga Y, et al; Three-year outcomes of surgical versus endovascular revascularization for critical limb ischemia: The SPINACH Study (Surgical Reconstruction Versus Peripheral Intervention in Patients with Critical Limb Ischemia). Circ Cardiovasc Interv 2017; 10:
8. Conte MS, Bradbury AW, Kolh P, White JV, Dick F, Fitridge R, et.al. Global vascular guidelines on the management of chronic limb-threatening ischemia. Eur J Vasc Endovasc Surg 2019; 58: S1-S109.
9. Furukoshi M, Moriwaki T, Nakayama Y. Development of an in vivo tissue-engineered vascular graft with designed wall thickness (Biotube type C) based on a novel caged mold. J artif Organs 2016; 19:54-61
10. Ishi D, Enmi J, Moriwaki T, Ishibashi-Ueda H, Kobayashi M, Iwana S et al. Development of in vivo tissue-engineered microvascular grafts with an ultra small diameter of 0.6 mm (MicroBiotubes): acute phase evaluation by optical coherence tomography and magnetic resonance angiography. J artif Organs 2016; 19: 262-9
11. Ishi D, Enmi J, Iwai R, Kurisu K, Tatsumi E, Nakayama Y. One year rat study of iBTA-induced “Microbiotube” maicrovascula grafts with an ultra-small diameter of 0.6 mm. Eur J Vasc Endovasc Surg 2018; 55: 882-7
12. Nakayama Y, Furukoshi M. Terazawa T, Iwai R. Development of long in vivo tissue-engineered “Biotube” vascular graft. Biomaterals 2018; 185: 232-9.
13. Nakayama Y, Higashita R, Shiraishi Y, Umeno T, Tajikawa T, Yamada A et. al. iBTA-induced Biotube blood vessels: 2020 update. Kidney Dial 2021; 1: 3-13.

14. Higashita R, Nakayama Y, Shiraishi Y, Iwai R, Inoue Y, Yamada A, et al. Acute phase pilot evaluation of small diameter long iBTA induced vascular graft "Biotube" in a goat model. EJVES Vasc Forum. 2022; 54: 27–35.
15. Mills Sr JL Conte MS, Armstrong DG, Pomposelli FB, Schanzer A, Sidawy AN et al. The Society for Vascular Surgery Lower Extremity Thretened Limb Classification System: risk stratification based on wound, ischemia, and foot infection (WIfI). J Vasc Surg 2014; 59: 220-34.
16. Schanzer A, Hevelone N, Owens CD, Belkin M, Bandyk DF, Clowes AW et al. Technical factors affecting autogenous vein graft failure: Observations from a large multicenter trial. J Vasc Surg 2007; 46: 1180-90.
17. Slim H, Tiwari A, Ritter JC, Rashid H. Outcome of infra-inguinal bypass grafts using conduit with less than 3 millimeters diameter in critical leg ischemia. J Vasc Surg 2011; 53: 421-5.
18. Hata Y, Iida O, Takahara M, Asai M, Masuda M, Okamoto S et al. Saphenous vein size as a surrogate marker for mortality of patients with chronic limb-threatening ischemia undergoing endovascular therapy. J Cardiol 2021; 78: 341-6.
19. Yamamoto S, Deguchi J, Hashimoto T, Suhara M, Sato O. Relationship between the controlling nutritional status score and infrainguinal bypass surgery outcomes in patients with chronic limb-threatening ischemia. Ann Vasc Dis 2021; 14: 334-6
