# Supplementary material for: Safety and efficacy of an iBTA-induced autologous Biotube vascular graft and its preparation device BTM1 in below-the-knee bypass surgery for chronic limb threatening ischemia: A protocol for an open-label, single-arm, multicenter clinical trial
Source: PLoS One. 2025 Nov 6;20(11):e0335900. doi: 10.1371/journal.pone.0335900 (PMC12591420; doi:10.1371/journal.pone.0335900)
Supplement: Attachment — Submitted filename: response to reviewer.docx [file pone.0335900.s005.docx]

**Response to reviewers**

**Comment 1**: Please ensure that your manuscript meets PLOS ONE's style requirements, including those for file naming.

**Response**: The manuscript has been revised to conform to PLOS ONE’s style requirements.

**Comment 2**: a. Please provide an amended Funding Statement declaring this commercial affiliation, as well as a statement regarding the Role of Funders in your study. If the funding organization did not play a role in the study design, data collection and analysis, decision to publish, or preparation of the manuscript and only provided financial support in the form of authors' salaries and/or research materials, please review your statements relating to the author contributions, and ensure you have specifically and accurately indicated the role(s) that these authors had in your study. You can update author roles in the Author Contributions section of the online submission form. Please also include the following statement within your amended Funding Statement.
“The funder provided support in the form of salaries for authors [insert relevant initials], but did not have any additional role in the study design, data collection and analysis, decision to publish, or preparation of the manuscript. The specific roles of these authors are articulated in the ‘author contributions’ section.”
If your commercial affiliation did play a role in your study, please state and explain this role within your updated Funding Statement.

b. Please also provide an updated Competing Interests Statement declaring this commercial affiliation along with any other relevant declarations relating to employment, consultancy, patents, products in development, or marketed products, etc. Please include both an updated Funding Statement and Competing Interests Statement in your cover letter. We will change the online submission form on your behalf.

**Response**: Thank you for your detailed instructions regarding the Funding Statement and Competing Interests Statement. In accordance with your request, we have revised these sections as follows and included them in the cover letter.

**Funding Statement (amended):**
This study is supported by the Japan Agency for Medical Research and Development (AMED). The study devices were produced and provided free of charge by Biotube Co., Ltd. This is an investigator-initiated clinical trial. The funders had no role in the study design; collection, management, analysis, or interpretation of data; writing of the manuscript; or the decision to submit the report for publication. The specific roles of the authors affiliated with Biotube Co., Ltd. are articulated in the “Author Contributions” section.

**Competing Interests Statement (amended):**
YN (Yasuhide Nakayama) and RH (Ryuji Higashita) are employees and stockholders of Biotube Co., Ltd. The other authors have declared that no competing interests exist. This does not alter our adherence to PLOS ONE policies on sharing data and materials.

We trust that these revisions meet the journal’s requirements.

**Comment 3,4**:  Please provide a complete Data Availability Statement in the submission form, ensuring you include all necessary access information or a reason for why you are unable to make your data freely accessible. If your research concerns only data provided within your submission, please write "All data are in the manuscript and/or supporting information files" as your Data Availability Statement. When completing the data availability statement of the submission form, you indicated that you will make your data available on acceptance. We strongly recommend all authors decide on a data sharing plan before acceptance, as the process can be lengthy and hold up publication timelines. Please note that, though access restrictions are acceptable now, your entire data will need to be made freely accessible if your manuscript is accepted for publication. This policy applies to all data except where public deposition would breach compliance with the protocol approved by your research ethics board. If you are unable to adhere to our open data policy, please kindly revise your statement to explain your reasoning and we will seek the editor's input on an exemption. Please be assured that, once you have provided your new statement, the assessment of your exemption will not hold up the peer review process.

**Response**: Thank you for your important comment. As this manuscript is a study protocol, no datasets have yet been generated or analyzed. We fully understand and agree with PLOS ONE’s open data policy. Accordingly, we have revised our Data Availability Statement as follows:

“*No datasets were generated or analyzed during the current study. Upon completion of the trial, all relevant anonymized data will be made publicly available in an appropriate public repository, in accordance with ethical and legal requirements. “*

We believe this revision clarifies our data sharing plan and ensures compliance with the journal’s policy.

**Comment 5**: If the reviewer comments include a recommendation to cite specific previously published works, please review and evaluate these publications to determine whether they are relevant and should be cited. There is no requirement to cite these works unless the editor has indicated otherwise.

**Response**: We note that no specific previously published works were suggested for citation in the reviewer comments. Therefore, no additional references were added. We have ensured that the current reference list adequately reflects the most relevant literature for the study.

**Comment 6**: Please review your reference list to ensure that it is complete and correct. If you have cited papers that have been retracted, please include the rationale for doing so in the manuscript text, or remove these references and replace them with relevant current references. Any changes to the reference list should be mentioned in the rebuttal letter that accompanies your revised manuscript. If you need to cite a retracted article, indicate the article’s retracted status in the References list and also include a citation and full reference for the retraction notice.

**Response**: We have carefully reviewed our reference list and confirm that all cited publications are complete and correct. None of the references cited in our manuscript are retracted articles. Therefore, no changes were made to the reference list.

**Comments to the Author**

**Comment 1**: Does the manuscript provide a valid rationale for the proposed study, with clearly identified and justified research questions?

Reviewer #1: Yes

Reviewer #2: Yes

Reviewer #3: Yes

**Response**: We sincerely thank all reviewers for their positive evaluation. We are pleased that the rationale and research questions of our study were recognized as clear and valid.

**Comment 2**:  Is the protocol technically sound and planned in a manner that will lead to a meaningful outcome and allow testing the stated hypotheses?

Reviewer #1: Yes

Reviewer #2: Yes

Reviewer #3: Yes

**Response**: Response: We greatly appreciate the reviewers’ positive evaluations. We are pleased that the study protocol was recognized as technically sound and appropriately designed to allow meaningful outcomes and proper testing of the stated hypotheses.

**Comment 3**: Is the methodology feasible and described in sufficient detail to allow the work to be replicable?

Reviewer #1: Yes

Reviewer #2: Yes

Reviewer #3: Yes

**Response**: We sincerely thank all reviewers for their positive assessments. We are encouraged that the methodology was considered feasible and described in sufficient detail to allow replication of the study.

**Comment 4**: Have the authors described where all data underlying the findings will be made available when the study is complete?

Reviewer #1: No

Reviewer #2: Yes

Reviewer #3: Yes

**Response**: We thank the reviewers for their comments regarding the Data Availability Statement. As this manuscript is a study protocol, no datasets have yet been generated or analyzed. To clarify our data sharing plan in line with PLOS ONE’s data policy, we have revised the Data Availability Statement to read as follows:

“No datasets were generated or analyzed during the current study. Upon completion of the trial, all relevant anonymized data will be made publicly available in an appropriate public repository, in accordance with ethical and legal requirements.”

We believe this revision addresses the concern and provides sufficient detail about where and how the data will be made available once the study is complete.

**Comment 5**: Is the manuscript presented in an intelligible fashion and written in standard English?

Reviewer #1: Yes

Reviewer #2: Yes

Reviewer #3: Yes

**Response**: We sincerely thank the reviewers for their positive evaluation. We are pleased that the manuscript was considered to be intelligible and written in standard English. We will carefully check the revised version again to correct any minor typographical or grammatical errors.

**Comment 6**: Review Comments to the Author

Reviewer #1:

**Comment**: I cannot accept this article because it didn't present any results; it only described the protocol of the clinical trial.

**Response**: We thank the reviewer for this comment. We respectfully note that this manuscript is submitted as a **study protocol**, in accordance with the journal’s scope and guidelines. As such, it is intended to describe the study rationale, objectives, and methodology in detail prior to the completion of the trial, and it does not include study results at this stage. We believe that the protocol format is appropriate and valuable for ensuring transparency, reproducibility, and scientific rigor in the planned clinical trial.

Reviewer #2:

**Comment 1**: Abstract: In the methods section, I would like to highlight some key findings from the study.

**Response**: Thank you for your helpful comment. We have revised the Abstract accordingly. In the Methods section, we now specify the primary endpoint (successful formation of an implantable Biotube following subcutaneous embedding of the BTM1) and the key secondary endpoints (intraoperative usability, patency and biocompatibility of the Biotube, wound healing, relief of rest pain, limb salvage, and procedure-related mortality). These revisions clarify the planned outcomes to be assessed in this exploratory trial, in line with your suggestion.

**Comment 2**: Methods: The problem is the time it takes for the tube to form; patients with severe critical ischemia cannot always wait three months. What postoperative therapy should be used?

**Response**: We thank the reviewer for this important comment. We fully agree that patients with severe critical ischemia who require immediate revascularization cannot wait for the three-month subcutaneous embedding period necessary for Biotube formation. To address this, our exclusion criteria clearly state that patients “in whom it is difficult to ensure the implantation period required for Biotube formation due to systemic conditions, such as the need for urgent revascularization,” are excluded from the study.

With regard to postoperative management, appropriate antithrombotic therapy is provided to prevent thrombosis of the implanted Biotube. Specifically, heparin is administered for 7 days after Biotube implantation, followed by a switch to oral anticoagulants, which are continued until 12 weeks after implantation. This therapeutic regimen is described in the Intervention section of the protocol.

We believe these criteria and postoperative management adequately address the reviewer’s concerns regarding patient selection and therapy after Biotube implantation.

**Comment 3**: What would have been the alternative technique for the patients who will be enrolled?

**Response**: We thank the reviewer for this important comment. The patients to be enrolled in this study are those with chronic limb-threatening ischemia (CLTI) who require distal bypass surgery to tibial or pedal arteries but have no suitable autologous venous conduit. For such patients, there is currently no established pharmacological therapy with proven efficacy, and no suitable alternative medical device is available. If revascularization cannot be adequately performed, the only remaining option is major limb amputation.

As stated in the “Explanation for the choice of comparators” section, “There is no suitable alternative medical device for CLTI patients who require bypass surgery with saphenous veins. The fate of the affected limb if revascularization is not adequately performed is also clear.” Therefore, patients eligible for this trial essentially have no alternative to bypass surgery with a Biotube graft, aside from amputation.

**Comment 4**: With what other biomaterials will you compare the results obtained?

**Response**: We thank the reviewer for this question. In this trial, no comparator biomaterial will be used. The efficacy of autologous vein grafts for below-the-knee bypass is already well established in the literature, and patients enrolled in this study are those who lack suitable autologous veins. Therefore, there is no alternative biomaterial available for comparison, and the present study is designed as a single-arm exploratory trial to evaluate the feasibility, safety, and efficacy of the Biotube graft.

Reviewer #3:

**Comment 1**: Page 4 lines 97-100. Please revise the description since prosthetic graft with Miller cuff may be also performed.

**Response**: We thank the reviewer for this helpful comment. In the revised manuscript, we have clarified that although small-diameter prosthetic grafts are generally associated with poor outcomes, modifications such as the Miller cuff have been attempted. However, autologous venous conduits remain the gold standard for below-the-knee bypass surgery, and their use is limited by the availability of suitable veins. The revised text now reads as follows:

*“Bypass with small-diameter prosthetic grafts has generally been associated with poor outcomes. Although modifications such as the use of a Miller cuff have been attempted to improve patency, autologous venous conduits remain the gold standard, particularly for patients requiring below-the-knee bypass surgery. However, their use is limited by the lack of suitable veins. Consequently, in the absence of proper veins, major amputation is often unavoidable. Amputation significantly worsens life prognosis. In other words, the inability to perform bypass surgery directly threatens patient survival.”*

**Comment 2**: Page 4 line 100. Amputation significantly worsens the life prognosis. Please include references.

**Response**: We thank the reviewer for this helpful suggestion. In accordance with the comment, we have added the following reference to support the statement that amputation significantly worsens life prognosis:

Leinweber ME, Greistorfer E, Rettig J, Taher F, Kliewer M, Assadian A et al. Quantification of the survival disadvantage associated with major amputation in patients with peripheral arterial disease. J Clin Med. 2025;14:104.

This citation has been incorporated into the revised manuscript.

**Comment 3**: Table 1, Inclusion criteria 3. Ischemia grades range from 0 to 3, and WIfI is a stage, not a grade.

**Response**: We thank the reviewer for pointing this out. You are correct that in the SVS WIfI classification, each component (Wound, Ischemia, and foot Infection) is graded from 0 to 3, and there is no grade 4. In our original text, we mistakenly wrote “grade 3 or 4” for ischemia. We have revised this to correctly read “Lower limb ischemia that meets grade 2 or 3 (ABI < 0.6, SPP < 40 mmHg) in WIfI classification of CLTI”. We appreciate the reviewer’s careful observation, which has improved the accuracy of our description.

**Comment 4**: Page 9 lines 230-231. Will the graft harvesting and bypass be performed on the same day? What circumstances would be expected if the same-day implementation is not possible?

**Response**: We thank the reviewer for this important question. In principle, removal of the Biotube and bypass surgery are performed on the same day under general anesthesia. However, if the patient’s general condition is poor and prolonged anesthesia would present an excessive burden, the bypass procedure may be performed on a different day. In such cases, implantation of the Biotube must be carried out within 4 weeks of its removal.

As described in the “Intervention description” section, if removal of the investigational device and Biotube implantation cannot be performed on the same day for reasons on the part of the subject, temporary storage of the Biotube is permitted. The Biotube is kept sterile, soaked in a 10% alcohol solution, sealed, and stored at room temperature. The maximum storage period is 4 weeks, and Biotube implantation must be performed within this period.

We believe this approach ensures both patient safety and the feasibility of the procedure in cases where same-day surgery is not possible.

**Comment 7**: PLOS authors have the option to publish the peer review history of their article ([what does this mean?](https://track.editorialmanager.com/CL0/https:%2F%2Fjournals.plos.org%2Fplosone%2Fs%2Feditorial-and-peer-review-process%23loc-peer-review-history/1/010f019912f5fbb3-ade56a25-fe8f-4a4d-aede-b76822a1e2de-000000/cbeRTfg5R6VlluRMxsZVy9frBeMmi7FFlIkyrFqu0vc=227)). If published, this will include your full peer review and any attached files. If you choose “no”, your identity will remain anonymous but your review may still be made public. **Do you want your identity to be public for this peer review?**

Reviewer #1: No

Reviewer #2: **Yes:** Francesco Stilo

Reviewer #3: **Yes:** Koichi Morisaki

**Response**: We would like to opt in to publish the peer review history of our article. We believe that making the peer review process publicly available will enhance transparency and contribute to the principles of open science.
