# Supplementary material for: Safety and efficacy of an iBTA-induced autologous Biotube vascular graft and its preparation device BTM1 in below-the-knee bypass surgery for chronic limb threatening ischemia: A protocol for an open-label, single-arm, multicenter clinical trial
Source: PLoS One. 2025 Nov 6;20(11):e0335900. doi: 10.1371/journal.pone.0335900 (PMC12591420; doi:10.1371/journal.pone.0335900)
Supplement: Attachment — Submitted filename: response to reviewer(2).docx [file pone.0335900.s006.docx]

**Response to reviewer**

**Comment 6**: Review Comments to the Author

Reviewer #4:

**Comment 1**: The justification of the sample size is unclear. With two device types and four implant locations, there are up to eight possible combinations. With only 12 subjects, these combinations will not be fully represented.

**Response**: We thank the reviewer for this important comment regarding the justification of the sample size. We agree that there are two device types and four possible implantation sites, resulting in up to eight potential combinations. However, because the choice of device size and implantation site is determined by the investigators according to the condition most suitable for each subject, it is not feasible to equally distribute the cases across all combinations in this exploratory study. The purpose of this trial is not to statistically compare all possible combinations, but rather to evaluate feasibility and safety under practical clinical conditions. In order to allow for the assessment of each condition (device type and implantation site) in more than one subject and to maintain feasibility of trial conduct, we determined that a minimum of 12 cases would be required. Based on these considerations and the inclusion of an interim analysis, we set the target sample size at 12 subjects. We have revised the text in the “Sample size” section accordingly.

**Comment 2**: Outcomes: The definition of the primary endpoint is too generic and not consistent with its statistical definition.

**Response**: We thank the reviewer for pointing out that the definition of the primary endpoint was too generic and not consistent with its statistical definition. We have revised the “Outcomes” section to provide a more precise definition of the primary endpoint as “the successful formation of an implantable Biotube, defined as retrieval of a structurally intact Biotube of sufficient length and quality judged suitable for use in bypass surgery by the investigators.”

Furthermore, we have revised the “Statistical Methods” section to clarify that the primary endpoint will be analyzed as the proportion of successful cases with a 95% confidence interval. Secondary and safety endpoints will be summarized descriptively. We believe that these revisions provide a clearer and statistically consistent definition of the endpoints and their planned analyses.

**Comment 3**: The protocol should clearly specify the statistical method planned for analyzing the primary endpoint. If no hypothesis testing is not planned for the exploratory study, it needs to state that clearly. Descriptive measures such as success rates can be reported.

**Response**: We thank the reviewer for pointing out the need to more clearly specify the statistical method for analyzing the primary endpoint. We agree with this suggestion. In the revised manuscript, we have clarified that the primary endpoint will be analyzed as the success rate, defined as the proportion of subjects from whom a Biotube suitable for implantation can be retrieved, and that this proportion will be presented together with a 95% confidence interval. We have also stated explicitly that, given the exploratory nature of this trial, no formal hypothesis testing is planned and that all analyses will be descriptive in nature. We believe that these revisions provide a clearer and statistically consistent description of the planned analyses.

**Comment 4**: The purpose of the interim analysis is also unclear. Is it intended for safety monitoring or a futility check? With only half of the planned sample size, the analysis may not have sufficient power to draw meaningful conclusions.

**Response**: We thank the reviewer for raising this important point regarding the purpose of the interim analysis. We agree that with a small number of subjects, the interim analysis cannot provide statistically powered conclusions. We have therefore clarified in the revised manuscript that the interim analysis is intended for **safety monitoring and feasibility assessment only**, and not for hypothesis testing or a futility check. At the time when 6 subjects have reached 12 weeks after Biotube implantation, we will descriptively summarize the Biotube-forming ability and short-term patency to ensure that no unexpected safety issues arise and that the procedure remains feasible. The results of the interim analysis will not be used for formal statistical inference but only to support the safe continuation of the study.
